# A Blood‐Brain Barrier‐Penetrant Ag(III) Corrole Compound Rescues Alzheimer's Disease Pathology by Targeting Aβ42‐Induced Oxidative Stress

**DOI:** 10.1002/advs.202515462

**Published:** 2026-01-08

**Authors:** Arup Tarai, Tuhina Mitra, Tanmoy Pain, Jyotiprakash Mallick, Rwiddhi Chakraborty, Kallam Tejaswi, Swagata Ghatak, Sanjib Kar

**Affiliations:** ^1^ School of Chemical Sciences National Institute of Science Education and Research (NISER) Bhubaneswar 752050 India; ^2^ An OCC of HBNI Training School Complex Anushakti Nagar Mumbai 400 094 India; ^3^ Department of Chemistry Mahila Mahavidyalaya (MMV) Banaras Hindu University (BHU) Varanasi 221005 India; ^4^ School of Biological Sciences National Institute of Science Education and Research (NISER) Bhubaneswar 752050 India

**Keywords:** alzheimer's disease, amyloid‐beta peptide, central nervous system disorder, reactive oxygen species, silver (III) corrole

## Abstract

Alzheimer's disease (AD) lacks disease‐modifying therapies, partly due to the blood‐brain barrier (BBB) limiting drug delivery and the multifaceted toxicity of amyloid‐β (Aβ42) oligomers. Here, this work reports the design, synthesis, and comprehensive characterization of a rationally designed silver(III) corrole complex, **(Mor‐Cor)Ag(III)** featuring a morpholino substituent at the periphery of the macrocycle, engineered to overcome these challenges through: (1) a morpholino moiety enabling BBB penetration (validated by mass spectrometry and fluorescence imaging), and (2) a redox‐active Ag(III) center that scavenges Aβ42‐induced reactive oxygen species (ROS). Detailed structural, spectroscopic, and density functional theory (DFT) analyses are performed to elucidate the electronic features of the complex. Further through multimodal biological validation in in vitro and in vivo transgenic AD models, **(Mor‐Cor)Ag(III)** is shown to disrupt Aβ42 aggregation and decrease ROS levels leading to decrease in dystrophic neurites, neuronal hyperactivity and neuronal death. **(Mor‐Cor)Ag(III)** significantly outperformed its unsubstituted analog, **(Cor)Ag(III)** in its ability to provide neuroprotection. This work establishes morpholine‐appended metallocorroles as a new class of neuroprotective drugs to address unmet therapeutic needs in AD.

## Graphical abstract

The newly designed metallo‐corrole **(Mor‐Cor)Ag(III)** with a morpholino group exhibited promising therapeutic potential for Alzheimer's disease. It significantly reduces reactive oxygen species (ROS), decreases GFAP expression, increases neurite length, and decreases cell death compared to other similar metallo‐corroles.

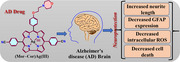

## 1 Introduction

**Figure 1:**
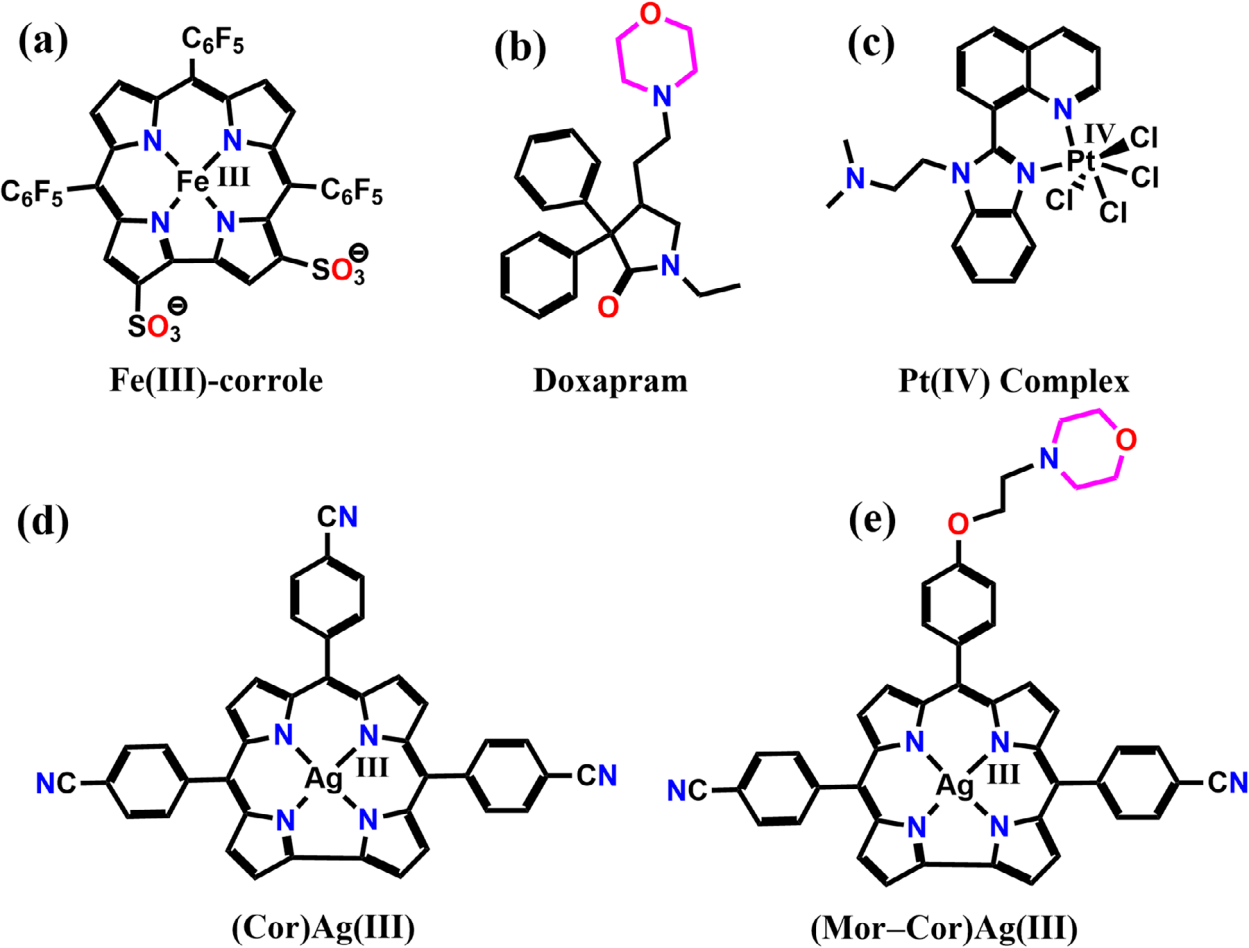
The chemical structure of a) **Fe(III)‐corrole**,^[^ ^28^ ^]^ b) **Doxapram**,^[^ ^38^ ^]^ c) **Pt(IV) complex,** ^[^ ^55^ ^]^ d) 5,10,15‐Tris(4‐cyanophenyl)corrolato silver(III), **(Cor)Ag(III)** ^[^ ^71^ ^]^ and e) [10‐{4‐(2‐morpholinoethoxy)phenyl}‐5,15‐bis‐(4‐cyanophenyl)‐corrolatosilver(III), **(Mor‐Cor)Ag(III)**.

Given the aging global population, developing treatments for central nervous system (CNS) disorders such as Alzheimer's is crucial.^[^ ^1^, ^2^, ^3^, ^4^, ^5^, ^6^ ^]^ In Alzheimer's disease (AD) there is an increased accumulation of Aβ oligomers and an increased ratio of Aβ42/Aβ40.^[^ ^7^, ^8^, ^9^, ^10^, ^11^ ^]^ Aβ42 oligomers cause increased accumulation of ROS and intracellular calcium.^[^ ^12^, ^13^, ^14^ ^]^ Among the various factors that have been linked with the cause and progression of AD, excessive generation of ROS is one of the prominent ones.^[^ ^14^ ^]^ Accumulation of Aβ40 and Aβ42 peptides in Alzheimer's disease is linked to enhanced oxidative damage to proteins, lipids, and nucleic acids in the hippocampus and cortex.^[^ ^13^ ^]^ Increased concentrations of metal ions like copper and iron coordinated to Aβ can lead to excess ROS production like H_2_O_2_ and HO^•^.^[^ ^13^ ^]^ Additionally, Aβ is known to cause mitochondrial toxicity, promoting leakage of ROS.^[^ ^14^, ^15^ ^]^ ROS are involved in cellular signalling and play a crucial role in mitochondrial function and the immune response to pathogens.^[^ ^16^, ^17^, ^18^ ^]^ However, excess ROS damages cellular macromolecules leading to cell death.^[^ ^19^, ^20^ ^]^ An imbalance between ROS generation and decomposition is detrimental to the cells, triggering pro‐inflammatory signalling, necrosis, and apoptosis.^[^ ^18^, ^20^ ^]^ The higher lipid content and rapid metabolic rate make the brain more susceptible to the detrimental effects of ROS.^[^ ^21^ ^]^ In AD, ROS accumulates in the hippocampus and cortex, with the entorhinal cortex and CA1 region of the hippocampus being the major susceptible regions.^[^ ^22^ ^]^ Superoxide anion, hydrogen peroxide, hydroxyl radical, and nitric oxide are the major ROS that have been implicated in oxidative stress‐mediated neurodegeneration in AD.^[^ ^17^, ^23^ ^]^ Activation of microglia in response to degenerated neurons and neuronal lesions generates ROS and causes eventual oxidative stress.^[^ ^24^ ^]^ Additionally, mitochondrial autophagy also acts as a major source of ROS production.^[^ ^15^ ^]^ The resulting increased oxidative stress in neurons leads to abnormal energy metabolism, synaptic dysfunction, degeneration of its dendrites and axons and subsequently death of the neurons.^[^ ^23^, ^25^ ^]^ Metallocorroles can decompose a variety of ROS and have been shown to be neuroprotective in optic neuropathies.^[^ ^26^, ^27^ ^]^ Metallocorroles like Fe(III) complex of the amphipolar 2,17‐bis‐sulfonato‐5,10,15‐tris(pentafluorophenyl)corrole (**Figure** **1**a) has been previously shown to bind to Aβ and to scavenge ROS.^[^ ^28^ ^]^ On the other hand, morpholine in CNS‐active compounds is used to increase the efficacy of the compounds through molecular interactions, by acting as scaffolds and by modulating pharmacological characteristics of the compound.^[^ ^29^ ^]^ For example, in case of AD, morpholines acted as important molecular scaffolds leading to better BACE‐1 (an enzyme that cleaves the extracellular domain of the amyloid precursor protein in combination with γ‐secretase leading to the production of pathogenic neurotoxic peptides Aβ40 and Aβ42) inhibition.^[^ ^29^ ^]^ Interestingly, morpholine containing compounds have been shown to decrease the formation of H_2_O_2_ and other ROS.^[^ ^30^, ^31^ ^]^ To enhance a drug's ability to cross the blood–brain barrier (BBB), it is essential to balance molecular size reduction with increased lipophilicity, thereby improving permeability and bioavailability.^[^ ^32^, ^33^ ^]^ Structural modifications that optimize these parameters are critical for developing therapeutics targeting central nervous system (CNS) disorders. Morpholine, a heterocyclic moiety frequently incorporated into CNS‐active drugs, allows for precise modulation of pharmacokinetic and pharmacodynamic (PK/PD) properties.^[^ ^34^, ^35^, ^36^, ^37^ ^]^ Its inclusion often leads to improved metabolic stability, solubility, and BBB penetration. Notably, several clinically approved morpholine‐containing compounds—such as doxapram (Figure 1b), phendimetrazine, moclobemide, reboxetine, and aprepitant—demonstrate the efficacy of this strategy across diverse CNS indications.^[^ ^38^ ^]^ Metal complexes of Pt,^[^ ^39^, ^40^, ^41^, ^42^ ^]^ Ru,^[^ ^43^, ^44^ ^]^ Co,^[^ ^45^, ^46^, ^47^ ^]^ Rh,^[^ ^48^ ^]^ Ir,^[^ ^49^, ^50^ ^]^ Mn,^[^ ^51^, ^52^ ^]^ Re,^[^ ^53^ ^]^ and V^[^ ^54^ ^]^ have shown promise as therapeutics for Alzheimer's disease (AD) by modulating the aggregation of the amyloid‐beta (Aβ) peptide. An orally administered platinum(IV) complex (Figure 1c) cuts Alzheimer's amyloid burden by 26% in transgenic mice, confirmed by brain tissue analysis.^[^ ^55^ ^]^ A new Ag(III) corrole with a morpholino group on the corrole periphery (Figure 1e) has been synthesized to evaluate its potential as a drug candidate for Alzheimer's disease (AD). Generally, transition‐metallocorroles including Ag(III)‐corrole complexes (Figure 1d), have undergone detailed investigation by several research groups and exhibit stability against hydrolysis under normal conditions.^[^ ^56^, ^57^, ^58^, ^59^, ^60^, ^61^, ^62^, ^63^, ^64^ ^]^ Silver has a long history of medical use, dating back to ancient times. Over time, silver salts have been employed to treat various conditions, including infections, brain disorders, mental illness, and more.^[^ ^65^, ^66^, ^67^, ^68^ ^]^ Additionally, they have been effective in treating specific ailments such as corneal ulcers, keratitis, blepharitis, and cystitis.^[^ ^69^, ^70^ ^]^ We examined the effect of **(Mor‐Cor)Ag(III)** on Aβ42 oligomer‐treated rat primary neuron‐astrocyte cultures and compared it to the effect of 5,10,15‐tris(4‐cyanophenyl)corrolato silver(III); namely **(Cor)Ag(III)** {an Ag(III)‐corrole lacking a morpholino group}^[^ ^71^ ^]^ treatment. We developed a rationale for the advantageous molecular design of **(Mor‐Cor)Ag(III)** over **(Cor)Ag(III)** and analyzed its impact on Aβ42 oligomer toxicity. Additionally, we compared the radical scavenging ability of **(Mor‐Cor)Ag(III)** to that of **(Cor)Ag(III)**.

## 2 Results and Discussion

### 2.1 Synthesis and Characterization

The free base (FB) corrole, 10‐{4‐(2‐morpholinoethoxy)phenyl}‐5,15‐bis‐(4‐cyanophenyl)corrole **H_3_** (**Mor‐Cor**), was synthesized following the general methodology for corrole synthesis developed by Gryko et al.^[^ ^72^ ^]^ The starting materials, namely 4‐(2‐morpholinoethoxy)benzaldehyde^[^ ^73^ ^]^ and 4‐cyanophenyldipyrromethane,^[^ ^72^ ^]^ were also prepared, according to earlier reports. The reported silver (III) complex **(Cor)Ag(III)** (without morpholine unit) and newly synthesized silver (III) complex, **(Mor‐Cor)Ag(III)** were achieved with slight modifications based on a previous procedure.^[^ ^63^ ^]^ The FB corrole, **H_3_** (**Mor‐Cor**) and silver (III) complex **(Mor‐Cor)Ag(III)** were fully characterized by various spectroscopic techniques such as IR, UV–vis, Fluorescence, and NMR, and the structure of the complex **(Mor‐Cor)Ag(III)** was obtained by single crystal structure analysis (Figures S1–S9, Supporting Information). The elemental analysis of **H_3_** (**Mor‐Cor**) and **(Mor‐Cor)Ag(III)** showed satisfactory results (see Experimental Section). In the FT‐IR spectroscopic studies, the N‐H stretching vibration of **H_3_** (**Mor‐Cor**) was observed at 3392 cm^−1^, but on complexation with Ag(III) metal ion, this N‐H stretching vibration disappeared. Similarly, on complexation, the C═N bonds stretching vibration of corrole ring in **H_3_** (**Mor‐Cor**) was shifted from 1597 to 1589 cm^−1^ in **(Mor‐Cor)Ag(III)**. However, the other bonds stretching vibration, such as C≡N and C‐H of **H_3_** (**Mor‐Cor**), remained nearly unchanged on complexation with Ag(III) metal ion (Figures S1 and S2, Supporting Information). The electrospray mass spectrometry analysis of **H_3_** (**Mor‐Cor**) in acetonitrile showed a molecular ion peak at *m/z* = 706.2935 corresponding to [**M**+H] ^+^ (Figure S3, Supporting Information). A similar analysis for **(Mor‐Cor)Ag(III)**, obtained a molecular ion peak at *m/z* = 810.1763, corresponding to [**M**+H] ^+^ (Figure S4, Supporting Information). Furthermore, the bulk purity and identity of newly synthesized FB corrole, **H_3_** (**Mor‐Cor**) and its Ag(III) analog, **(Mor‐Cor)Ag(III)** can be confirmed from the NMR spectroscopy. Both the compounds showed 20 aromatic peaks in the region δ, ≈8.99–7.30 ppm, and 12 aliphatic peaks in the region δ, ≈4.45–2.74 ppm (Figures S7 and S8, Supporting Information). A slight shift was observed in the ^1^H NMR spectra in the aromatic regions of **(Mor‐Cor)Ag(III)** compared to FB corrole **H_3_** (**Mor‐Cor**), attributed to the metalation process. Furthermore, the FB corrole **{H_3_** (**Mor‐Cor**)**}** and silver (III) complex**, (Mor‐Cor)Ag(III)** are structurally optimized by DFT calculation and presented in Figures S10 and S11, Supporting Information. The small distortion of the N4‐containing pyrrole ring in **H_3_** (**Mor‐Cor**) and **(Mor‐Cor)Ag(III)** was witnessed in the non‐planar distortions graph (Figures S10 and S11, Supporting Information).

### 2.2 Crystal Structure

**Figure 2:**
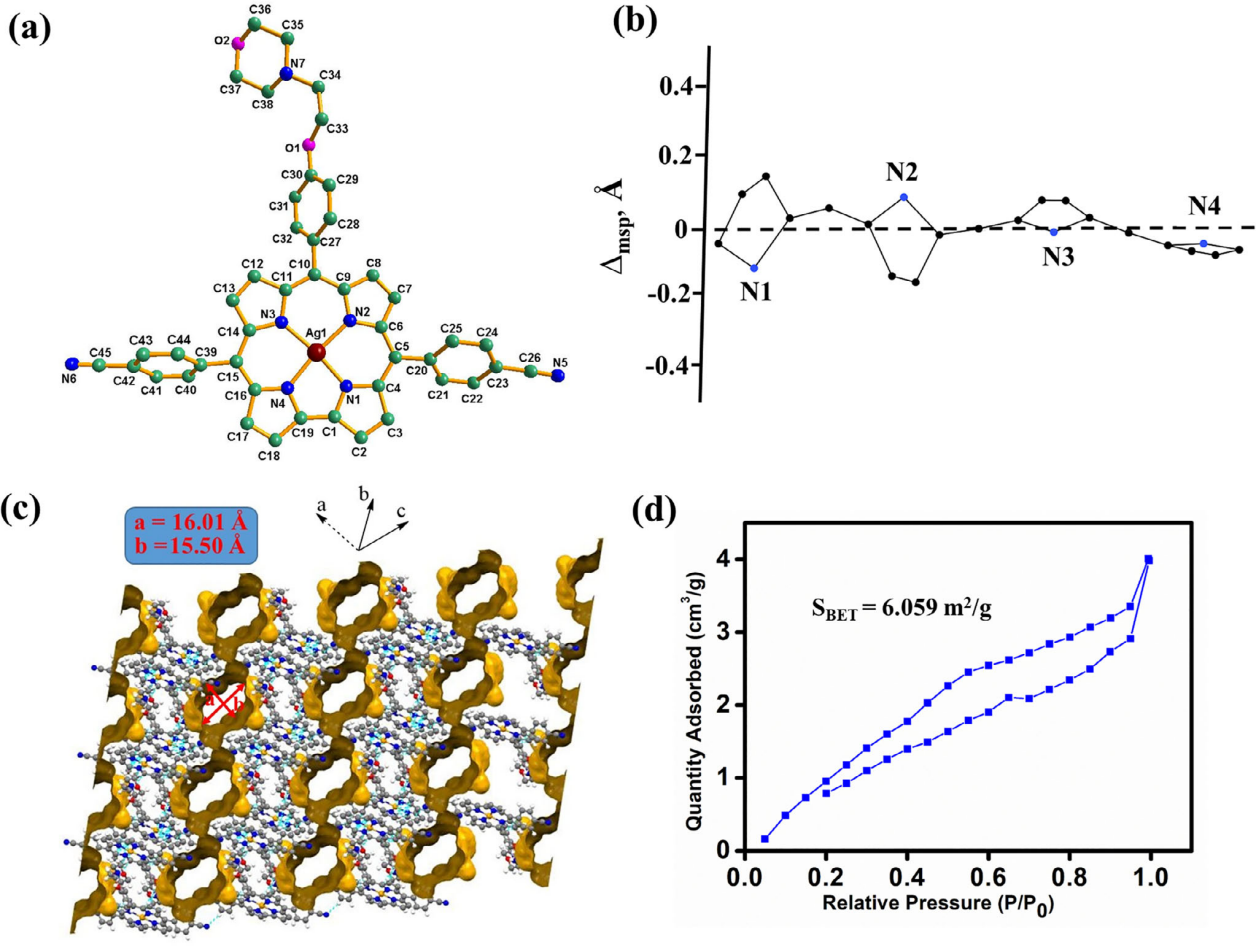
a) Single‐crystal X‐ray structure of **(Mor‐Cor)Ag(III)** (hydrogen atoms are removed for clarity), b) Linear display of non‐planar distortions, c) Extended hydrogen‐bonded porous supramolecular assembly of **(Mor‐Cor)Ag(III)** and d) Nitrogen adsorption‐desorption isotherms of **(Mor‐Cor)Ag(III)** at 77 K for BET surface area calculation.

The deep brown color block‐type crystals of **(Mor‐Cor)Ag(III)** were obtained from CH_2_Cl_2_‐hexane solvent mixtures on slow evaporation at ambient conditions. The complex **(Mor‐Cor)Ag(III)** crystallized in the P‐1 space group, displaying a square planar geometry. Key crystallographic parameters for this structure are provided in Table S1, Supporting Information. Refer to **Figure** **2**a, where the crystal structure is depicted for a visual representation. A *PorphyStruct* ^[^ ^74^, ^75^ ^]^ analysis of **(Mor‐Cor)Ag(III)** revealed various structural distortions (Figure 2b). The overall distortion (D_oop_) was calculated to be 0.4499 Å. Key distortions and their respective contributions to the total distortion include: doming distortion is −0.0428 Å (5.94% of the total distortion), saddling distortion is 0.6326 Å (50.27% of the total distortion), ruffling distortion is −0.0711 Å (9.86% of the total distortion), wavingX distortion is 0.1871 Å (25.95% of the total distortion) and wavingY distortion is 0.0280 Å (3.88% of the total distortion). The important bond distances of **(Mor‐Cor)Ag(III)** from the crystal structure and optimized structure are presented in Figure S12, Supporting Information and the Ag‐N bond distances are mentioned here; Ag‐N1, Ag‐N2, Ag‐N3 and Ag‐N4 are 1.936 (14) (DFT: 1.977 Å), 1.955 (15) (DFT: 1.987 Å), 1.956 (14) (DFT: 1.989 Å), and 1.934 (16) (DFT: 1.971 Å), respectively. The bite angles of N1‐Ag‐N2, N2‐Ag‐N3, N3‐Ag‐N4, and N4‐Ag‐N1 are 91.98 (6)° (DFT: 92.62°), 95.94 (6)° (DFT: 96.29°), 91.32 (6)° (DFT: 91.83°), and 80.92 (6)° (DFT: 80.05°), respectively. Interestingly, the observed Ag‐N bond distances were well‐matched with the previously reported corrolato‐Ag(III) metal complexes.^[^ ^63^ ^]^ The details of crystal structure elucidation reveal that the **(Mor‐Cor)Ag(III)** consists of various non‐covalent interactions, including C‐H···N and C‐H···π hydrogen bonds (Figures S13 and S14, Supporting Information). These interactions form an extended hydrogen‐bonding assembly resulting in a 2D porous supramolecular structure (Figure 2c).^[^ ^76^ ^]^ The important hydrogen bond parameters are tabulated in Table S2, Supporting Information. The porous assembly contains solvent‐accessible voids with a volume of 202.91 Å^3^, occupying 9.1% of the total unit cell volume. The dimensions of the voids are 16.01 × 15.50 Å, extending along the crystallographic a‐axis. These voids are empty and do not contain any solvent molecules, as confirmed by thermogravimetric analysis (TG). The TG analysis plot of **(Mor‐Cor)Ag(III)** is presented in Figure S15, Supporting Information, demonstrating that no weight loss associated with solvent molecules occurs up to 180 °C thereby confirming the absence of solvent molecules.

The porosity of **(Mor‐Cor)Ag(III)** was examined through Brunauer‐Emmett‐Teller (BET) analysis using nitrogen adsorption‐desorption isotherms at 77 K. The nitrogen adsorption‐desorption plots for **(Mor‐Cor)Ag(III)** is depicted in Figure 2d, revealing a BET surface area of 6.059 m^2^g^−1^. The very low surface area is likely attributed to the collapse of the hydrogen bonded porous structure during analysis.^[^ ^77^ ^]^

### 2.3 Absorption and Emission Spectroscopy

**Figure 3:**
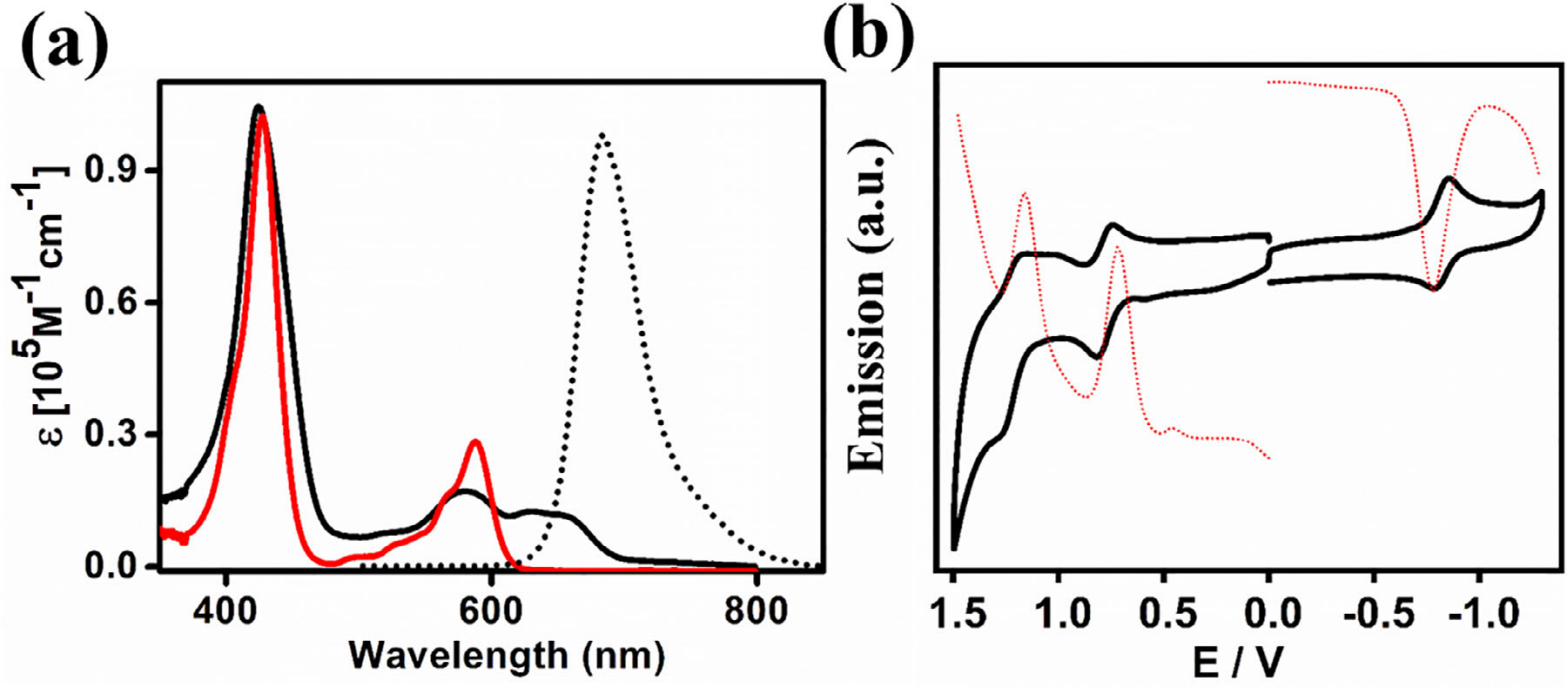
a) Absorption spectra of **H_3_** (**Mor‐Cor**) (black solid line), and **(Mor‐Cor)Ag(III)** (red solid line) and emission spectra of **H_3_** (**Mor‐Cor**) (black dotted line) in CH_2_Cl_2_ solution at 298 K, b) Cyclic voltammograms and differential pulse voltammogram of **(Mor‐Cor)Ag(III)** in CH_2_Cl_2_.

The electronic spectral data for the compounds in CH_2_Cl_2_ are shown in **Figure** **3** and summarized in Table S3, Supporting Information. The silver complex exhibits Soret bands ≈427 nm, consistent with values reported for similar compounds in the literature.^[^ ^63^ ^]^ To better understand the absorption characteristics of these compounds, TD‐DFT calculations were performed on **H_3_(Mor‐Cor)** and **(Mor‐Cor)Ag(III)**. For **H_3_** (**Mor‐Cor**), the primary transitions involve ligand‐based frontier orbitals. The most intense absorption band at 424 nm is attributed to a combination of transitions involving the HOMO‐1 to LUMO+1, HOMO to LUMO+2, and HOMO to LUMO+3 orbitals, typical for a Soret band of this intensity. In contrast, **(Mor‐Cor)Ag(III)**, which has a donor substituent on the corrolato ring, shows its most intense band at 427 nm.

This band results from transitions involving the HOMO‐1 to LUMO+1, HOMO to LUMO+2, HOMO‐5 to LUMO, and HOMO to LUMO+3 orbitals (Figures S16–S19 and Tables S4 and S5, Supporting Information). Additionally, transitions in the visible region for **(Mor‐Cor)Ag(III)** are partly due to intra‐ligand charge transfer (ILCT) or ligand‐to‐metal charge transfer (LMCT), influenced by the electron‐rich substituent on the corrolato ring. The emission spectrum of **H_3_** (**Mor‐Cor**) in CH_2_Cl_2_ is shown in Figure 3a (dotted line), with detailed data in Table S3, Supporting Information. When excited at the Soret band, **H_3_** (**Mor‐Cor**) exhibits strong emission, peaking at 683 nm.

### 2.4 Redox Properties

The redox properties of **(Mor‐Cor)Ag(III)** were examined in CH_2_Cl_2_/0.1 M TBAP using cyclic voltammetry and differential pulse voltammetry (Figure 3b and Table S3, Supporting Information). The oxidation and reduction processes on both the positive and negative sides of the Ag‐AgCl reference electrode were recorded using a platinum working electrode. The silver complex exhibits one reversible oxidation and one reversible reduction versus Ag‐AgCl. The oxidation peak was observed at E°_298_, V (ΔEp, mV): +0.77 (80), while the reduction peak was observed at E°_298_, V (ΔEp, mV): −0.83 (80) versus Ag‐AgCl. These redox potential data align well with previous literature.^[^ ^71^ ^]^ Based on this analogy, we have assigned the oxidation peak to corrole‐based oxidation, [(corrolato^•^^2−^)Ag(III)]^•+^/Ag(III), and the reduction peak to metal‐based reduction, Ag(III)/Ag(II).^[^ ^71^ ^]^

### 2.5 Neuroprotection Against Aβ42‐Related Neurotoxicity

**Figure 4:**
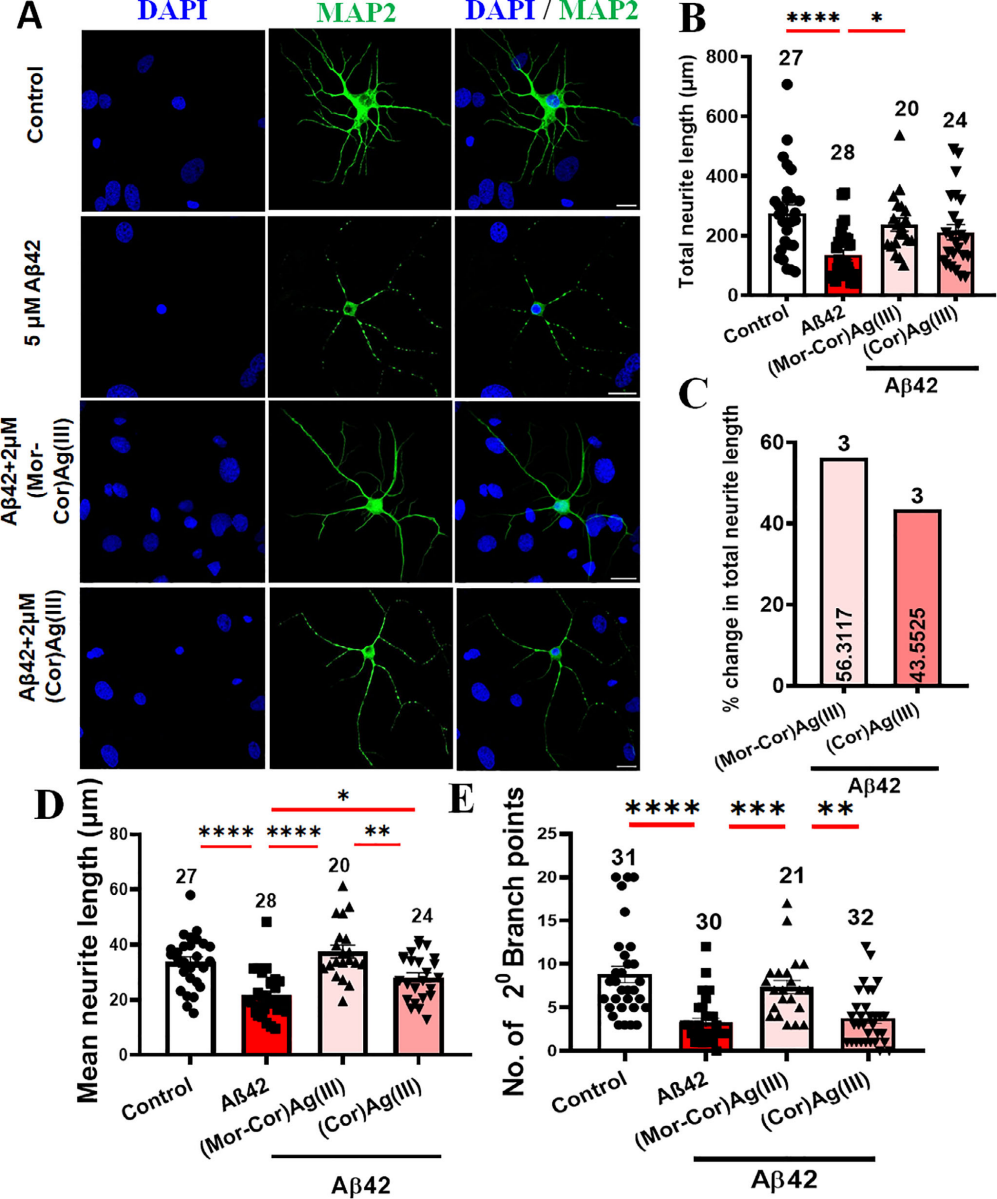
Aβ42‐ exposed neurons show increased neurite length and branches in the presence of **(Mor‐Cor)Ag(III)**. A) Representative images of neurons expressing MAP2. B) Quantification of the total neurite length. C) Quantification of percentage change in total neurite length. D) Quantification of mean neurite length. E) Quantification of total number of secondary neurite branchpoints. Scale bar: 20 µm. Data are mean ± SEM. Random fields selected for analysis. Data collected from 4 to 5 animals per group. Statistical significance analyzed by ANOVA with Sidak's multiple comparison test between selected pairs. Sample size listed above bar graphs (*p < 0.05; **p < 0.01; ***p < 0.001; **** *p* < 0.0001).

Aβ42 oligomers have been shown to be highly toxic and manifest greater neurotoxicity than protofibrils, fibrils, and plaques.^[^ ^78^, ^79^ ^]^ Here, we treated primary rat neuron‐astrocyte cultures with soluble Aβ42 oligomers and found that within 24 h, we observed increased neurite fragmentation similar to that observed in Alzheimer's disease (AD). Toward unravelling the therapeutic potential of **(Mor‐Cor)Ag(III)**, we first treated HT‐22 mouse hippocampal cells with **(Mor‐Cor)Ag(III)** at various concentrations and found that the compound did not show any toxicity at 6 µM and below (Figure S20, Supporting Information). We then went on to observe the protective potential of **(Mor‐Cor)Ag(III)** at 3 different concentrations (1, 2, and 6 µM) and found that 1–2 µM were optimal concentrations where we get maximum protection with 2 µM showing an improvement over 1 µM (Figure S20, Supporting Information). Thereafter, we used 2 µM of the compounds for treatment in a more physiological system of neuron‐astrocyte cultures freshly prepared from rat brains to study their effects. We observed that 2 µM treatment with both **(Mor‐Cor)Ag(III)** and **(Cor)Ag(III)** {without morpholine moiety} caused a decrease in the fragmentation (**Figures** **4**A,B,D and S21, Supporting Information).

**Figure 5:**
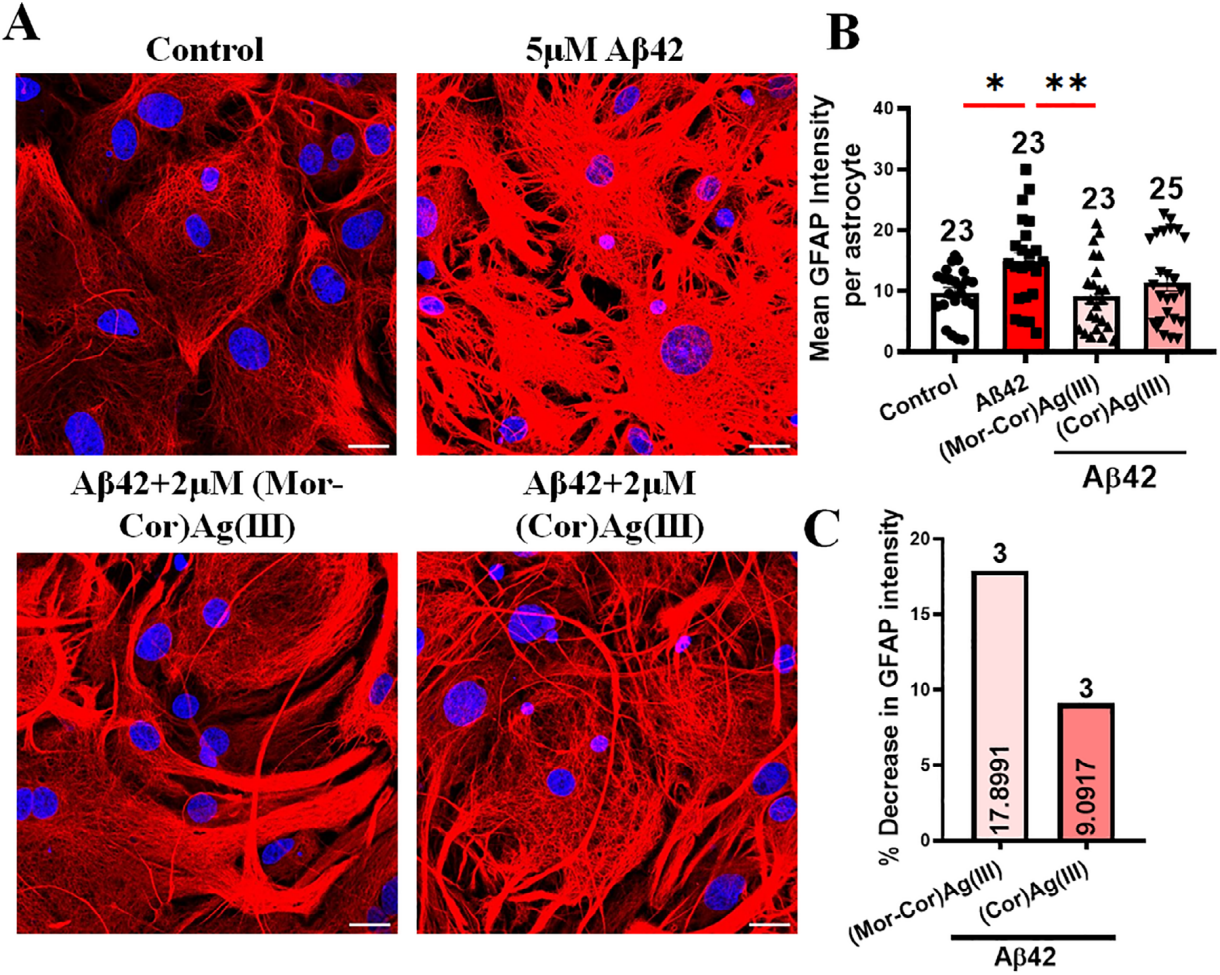
Aβ42‐ exposed cortical astrocytes show decreased GFAP expression in the presence of **(Mor‐Cor)Ag(III)**; A) Representative images of cells expressing the astrocyte marker GFAP. B) Quantification of the mean GFAP intensity per astrocyte. C) Quantification of percentage decrease in GFAP intensity in between two compound treated groups. Scale bar: 20 µm. Data are mean ± SEM. Random fields selected for analysis. Data collected from 4 to 5 animals per group. Statistical significance analyzed by ANOVA with Sidak's multiple comparison test between selected pairs. Sample size listed above bar graphs (*p < 0.05; **p < 0.01; ***p < 0.001; **** *p* < 0.0001).

However, **(Mor‐Cor)Ag(III)** was significantly more effective in decreasing the neurite fragmentation as indicated by the increased neurite length (Figure 4A,C,D and Figure S22, Supporting Information) and branchpoints (Figure 4A,E). Neither **(Mor‐Cor)Ag(III)** nor **(Cor)Ag(III)** exhibited any significant effect on neurite length or GFAP intensity in the absence of Aβ42 exposure (Figures S22 and S23, Supporting Information). In line with previous literature, astrocytes in neuron‐astrocyte cultures exposed to Aβ42 oligomers showed hypertrophy and increased GFAP expression^[^ ^80^ ^]^ (**Figure** **5**A,B). The GFAP expression decreased significantly in the presence of 2 µM **(Mor‐Cor)Ag(III)**, in contrast with the equimolar concentration of **(Cor)Ag(III)**, which did not have a significant effect (Figure 5A–C, Figure S21, Supporting Information). This suggests **(Mor‐Cor)Ag(III)** could prevent Aβ42‐mediated reactive gliosis.

### 2.6 **(Mor‐Cor)Ag(III)** Mitigates Aβ42‐Induced Neurotoxicity by Suppressing Hyperexcitability, Cell Death, and Oxidative Stress

**Figure 6:**
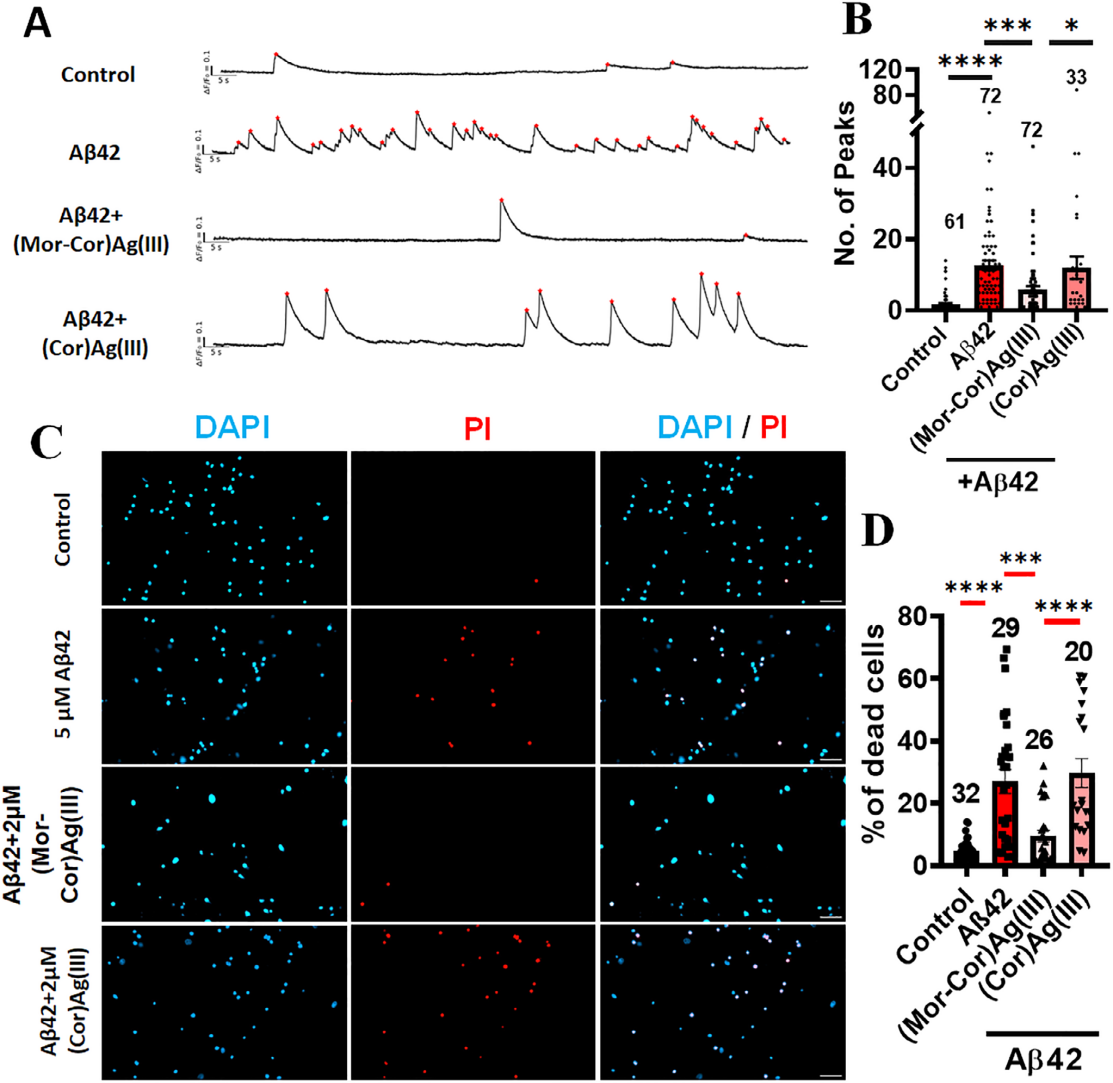
**(Mor‐Cor)Ag(III)** attenuates neuronal hyperexcitability and reduces cell death in Aβ42‐exposed neuron‐astrocyte cultures: A) Representative traces showing calcium transients using calcium imaging under each treatment condition; B) Quantitative analysis of calcium transients, illustrating a significant reduction in hyperexcitability in the compound‐treated group compared to the Aβ42‐treated condition. Data are presented as mean ± SEM. Statistical significance was analyzed using ANOVA followed by Sidak's multiple comparisons test between selected pairs (*p < 0.05; ***p < 0.001; ****p < 0.0001); C) Representative propidium iodide (PI) and DAPI‐stained fluorescence images of dissociated cortical neuron astrocyte cultures under 20× magnification; D) Quantification of the percentage of dead cells. Scale bar: 50 µm. Data are mean ± SEM. Random fields selected for analysis. Data collected from 4 to 5 animals per group. Statistical significance analyzed by Kruskal‐Wallis test with posthoc test, Dunn's multiple comparison test between selected pairs. Sample size listed above bar graphs (*p < 0.05; **p < 0.01; ***p < 0.001; ****p < 0.0001).

Primary rat cortical neurons exhibited significant hyperexcitability using calcium imaging upon treatment with Aβ1–42, as compared to the 0.1% DMSO vehicle‐treated controls (**Figure** **6**A,B, Videos S1–S4, Supporting Information), confirming the excitatory effect of Aβ1–42 on neuronal networks. Co‐treatment with **(Mor‐Cor)Ag(III)** (2 µM) notably attenuated this hyperactivity, while the parent complex **(Cor)Ag(III)** (2 µM) had no such effect (Figure 6A,B, Videos S1–S4, Supporting Information). These findings highlight the critical role of the morpholino substitution in mitigating Aβ1–42‐induced neuronal hyperexcitability, suggesting that the morpholine moiety may enhance compound‐cell interactions, facilitate target engagement, or modulate downstream signaling pathways.

Additionally, **(Mor‐Cor)Ag(III)** was also able to decrease neuronal death significantly compared to **(Cor)Ag(III)** treated cultures exposed to Aβ42 oligomers as observed by propidium iodide (PI) staining (Figure 6C,D). Next, we went on to investigate the mechanism by which **(Mor‐Cor)Ag(III)** was able to provide better neuroprotection.

**Figure 7:**
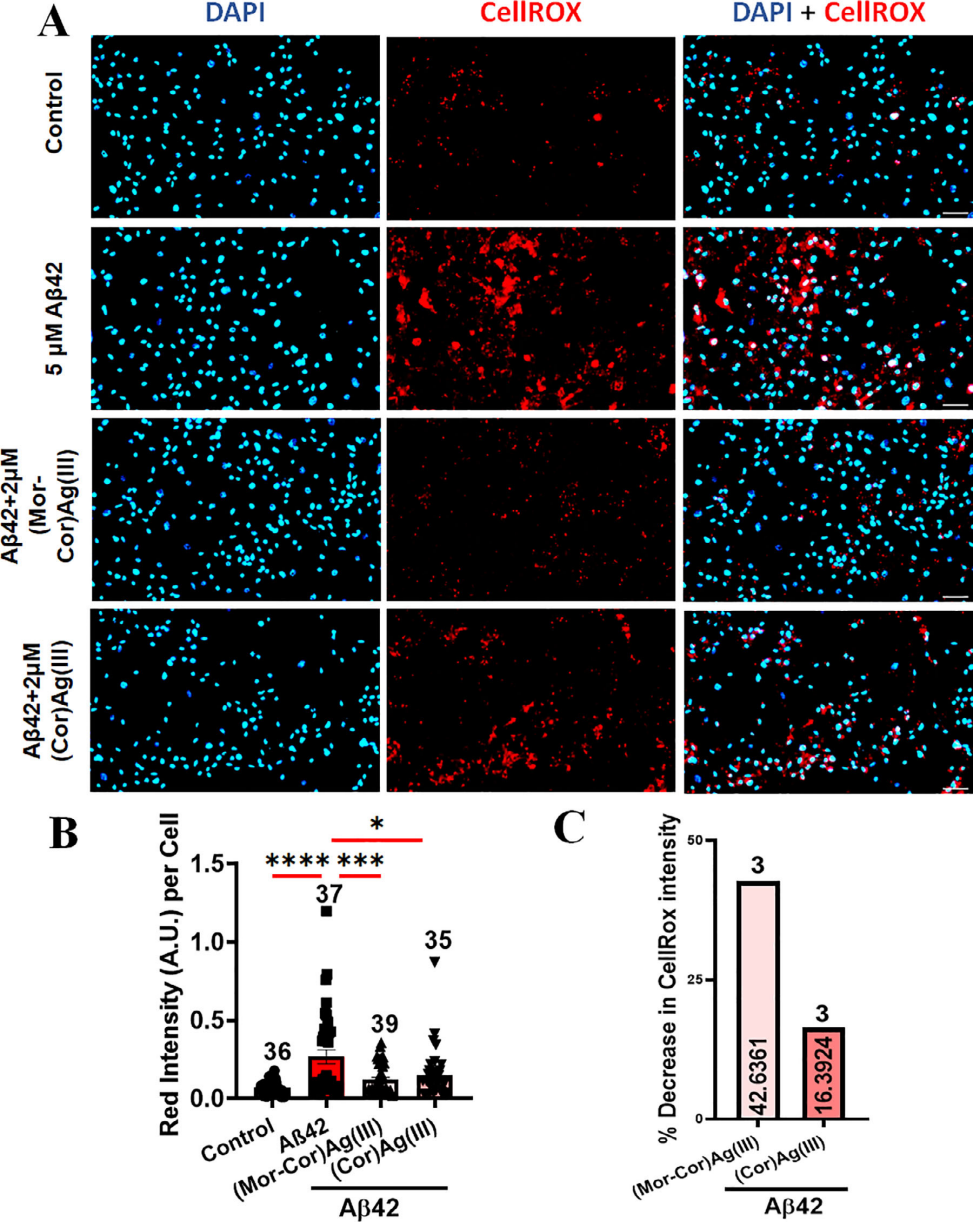
Aβ42‐ exposed neuron astrocyte culture showed decreased intracellular ROS levels in presence of **(Mor‐Cor)Ag(III)**: A) Representative images of cortical neuron astrocyte culture labeled with CellROX (deep red) for cytoplasmic ROS; B) The mean intensity of CellROX fluorescence in four different conditions were quantified; C) Quantification of percentage change in CellROX intensity in between two compounds. Scale bar: 50 µm. Data are mean ± SEM. Statistical significance analyzed by ANOVA with Sidak's multiple comparison test between selected pairs. Sample size listed above bar graphs (*p < 0.05; **p < 0.01; ***p < 0.001; **** *p* < 0.0001).

We detected the ROS levels using CellROX, fluorogenic probe for measuring cellular oxidative stress (**Figure** **7**A,B). We found that **(Mor‐Cor)Ag(III)** was able to decrease the ROS levels which were significantly increased in the Aβ42 oligomer‐treated neurons, to a greater extent than **(Cor)Ag(III)** (Figure 7A,C). Since ROS is detrimental to neuronal physiology and considered a mediator of Aβ42‐related neurotoxicity, the reduction in the ROS levels would lead to an improvement in neuronal health. This improvement can be observed in terms of decreased cell death, increased neurite length and branching, and decreased gliosis as described before (Figures 4, 5, 6).

In this study we have shown the increased efficiency of **(Mor‐Cor)Ag(III)** in providing neuroprotection compared to **(Cor)Ag(III)** against Aβ42‐related neurotoxicity. It was previously known that metallocorroles provide neuroprotection via decomposing ROS.

Fe‐ and Mn‐corroles were able to scavenge neuronal superoxide after optic nerve transection.^[^ ^26^ ^]^ Metal complexes containing manganese and iron, especially those with macrocyclic, salen, and porphyrin ligands, have been shown to catalyze the dismutation of superoxide (SO).^[^ ^81^ ^]^ The iron(III) and manganese(III) complexes of 3,17‐bis‐sulfonated corrole display superoxide dismutase (SOD) activity, which is linked to their M(IV)/M(III) redox potentials.^[^ ^28^, ^81^ ^]^

Research on their structure‐activity relationships has highlighted metal‐center redox potentials as key factors influencing this reactivity. Additionally, recent findings indicate that SOD activity can also arise from a redox‐active ligand, exemplified by the redox‐inactive metal zinc(II) when complexed with a hexadentate ligand.^[^ ^82^ ^]^ The silver(III) complexes discussed here have redox potentials similar to those of the iron(III) and manganese(III) corrole complexes, suggesting a possible correlation with their [(corrolato^•^^2−^)Ag(III)]^•+^/Ag(III) redox potential. We also observed a decrease in ROS levels upon treatment with **(Cor)Ag(III)**, an Ag‐corrole. However, we found that the addition of morpholine moiety to metallocorrole, **(Cor)Ag(III)** could enhance its ability to decrease abnormal ROS levels and, therefore, provide significantly better neuroprotection. The addition of morpholine moiety could increase the effectiveness of **(Cor)Ag(III)** by increasing its ability to decrease ROS via molecular interactions or by changing the pharmacological properties of the metallocorrole.^[^ ^29^ ^]^

### 2.7 BBB Permeability of **(Mor‐Cor)Ag(III)**

**Figure 8:**
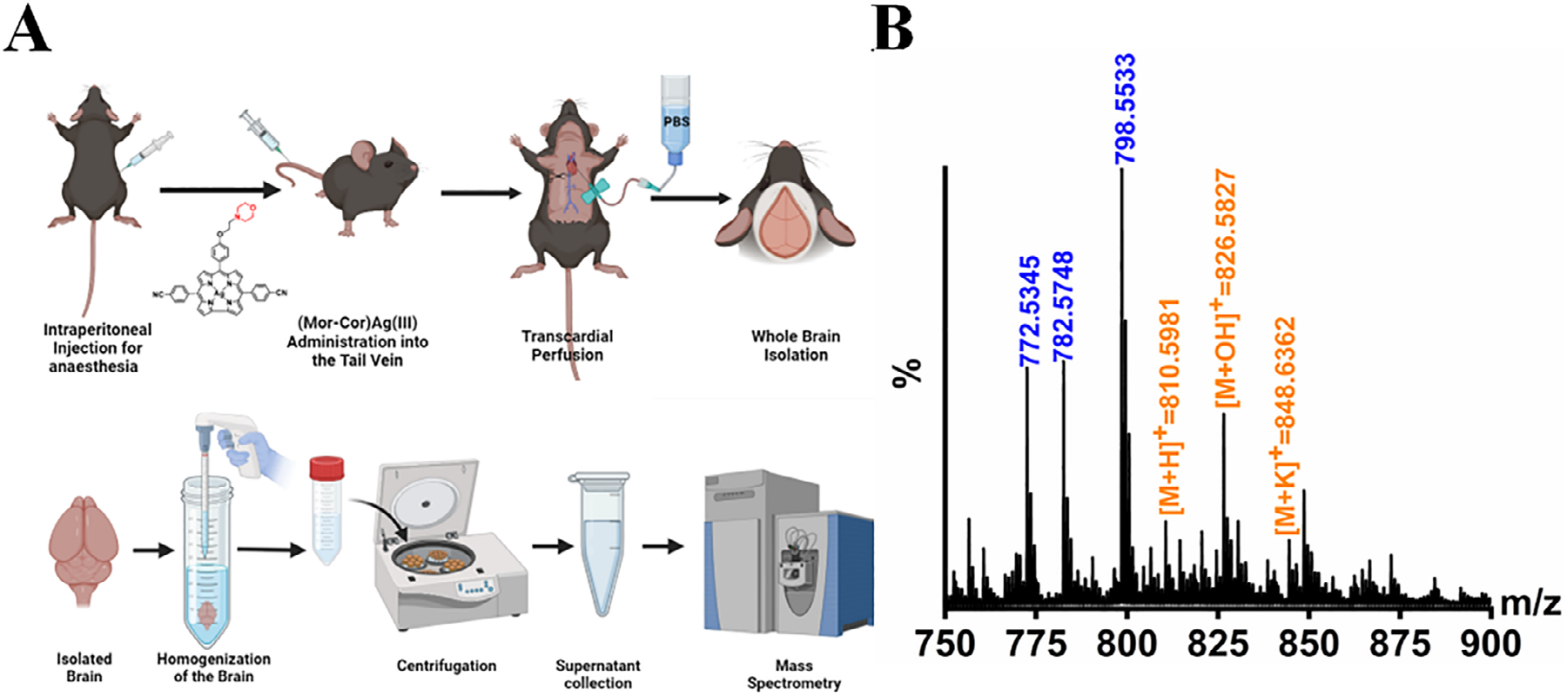
Mass spectrometry analysis of the murine brain after tail vein injection of **(Mor‐Cor)Ag(III)** indicates the ability of the compound to cross the blood‐brain barrier: A) Schematic illustration of the process of intravenous administration of the compound via tail vein injection, followed by brain isolation and subsequent processing to isolate the compound from the brain, demonstrating rapid brain targeting; B) The ESI‐MS spectrum of the sample in CH_3_CN displays the measured spectrum of the selected region, observed after intravenous administration of **(Mor‐Cor)Ag(III)** into the mouse via tail vein injection and subsequent brain isolation.

**Figure 9:**
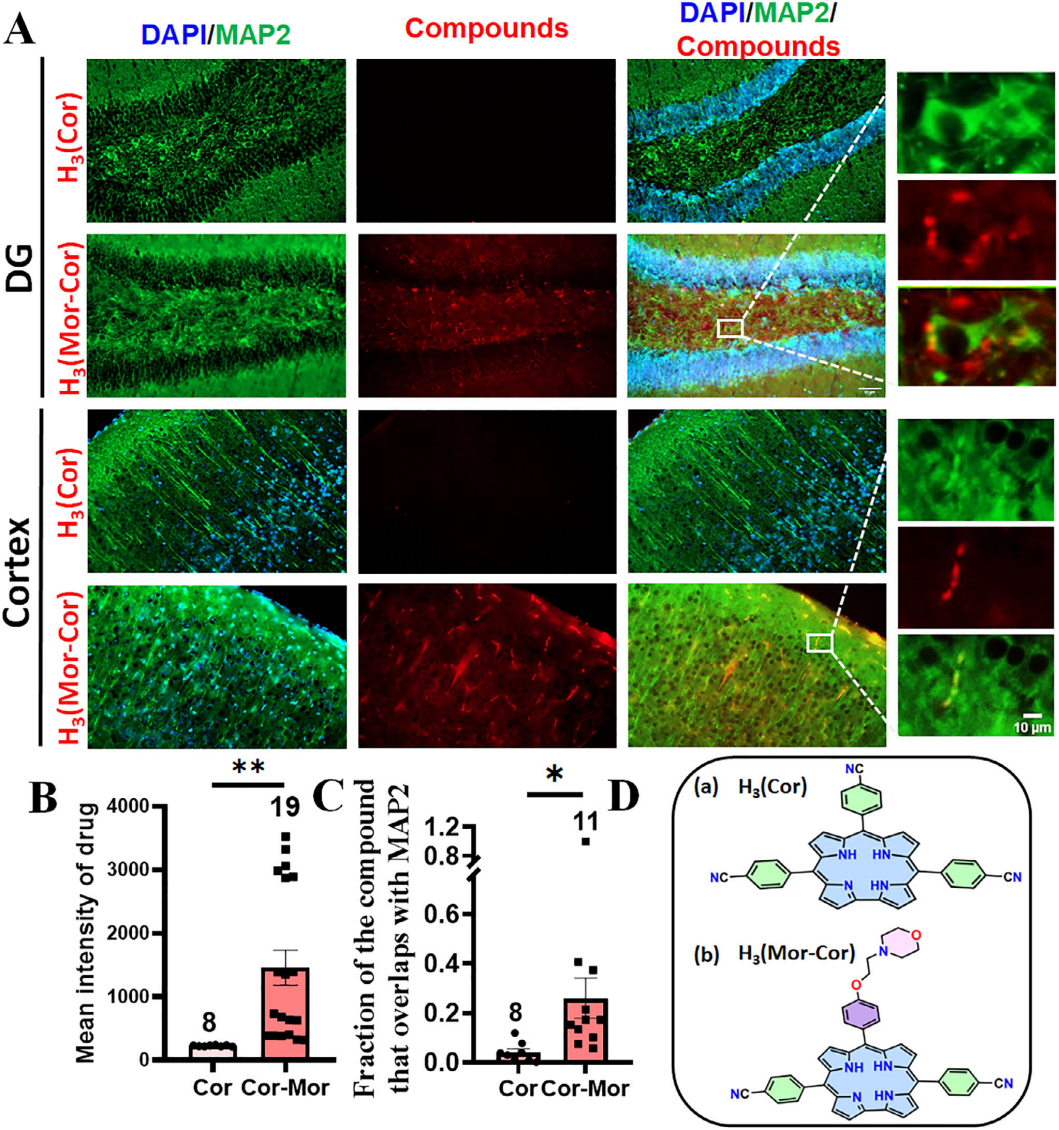
Fluorescence imaging reveals that the fluorescent compound conjugated with morpholino **{H_3_(Mor‐Cor)},** administered through tail vein injection, successfully crosses the blood‐brain barrier (BBB): A) The compound accumulates throughout the brain, including the cortex and hippocampus, confirming efficient BBB penetration and widespread distribution. Fluorescent compound without morpholino adduct {**H_3_(Cor)**} did not manifest any fluorescence in the brain suggesting its inability to cross the BBB (Scale bar = 50 µm); B) Quantification of fluorescence intensity reveals that {**H_3_(Mor‐Cor)**} efficiently crosses the blood‐brain barrier (BBB) compared to {**H_3_(Cor)**}; C) Colocalization of the compound with MAP2 confirms neuronal uptake of {**H_3_(Mor‐Cor)**} following successful penetration of the BBB; D) Chemical structure of a) 5,10,15‐Tris(4‐cyanophenyl)corrole, **H_3_(Cor)** and b) [10‐{4‐(2‐morpholinoethoxy)phenyl}‐5,15‐bis‐(4‐cyanophenyl)‐corrole, **H_3_(Mor‐Cor)**. Data are mean ± SEM. Statistical significance analyzed by unpaired *t*‐test (*p < 0.05; **p < 0.01). Data was acquired from 3 mice per groups.

In addition to studying the in vitro effects of **(Mor‐Cor)Ag(III)**, we also investigated its ability to cross the blood‐brain barrier (BBB). Since the morpholine moiety has been shown to increase the BBB crossing ability of several drugs tested in the CNS,^[^ ^29^, ^83^ ^]^ we injected **(Mor‐Cor)Ag(III)** via the tail vein in mice to study its ability to cross the BBB. Following a standardized protocol, we performed mass spectrometry (Figures S25 and S26, Supporting Information) on the supernatant extracted from the brain lysate of the mice (**Figure** **8**A). The presence of the compound was confirmed by analysing the mass spectrometry spectrum (Figure 8B, Figures S24–S26, Supporting Information). The examined compounds **(Mor‐Cor)Ag(III)** and **(Cor)Ag(III)** are non‐fluorescent, but their demetallated forms (free base, FB) {**H_3_(Mor‐Cor)**} and {**H_3_(Cor)**} are highly emissive. Therefore, to investigate the BBB crossing ability of **(Mor‐Cor)Ag(III)** and **(Cor)Ag(III)**, we injected a fluorescent alternative of the compound with {**H_3_(Mor‐Cor)**} and without morpholino {**H_3_(Cor)**} via the tail vein and analyzed the presence of fluorescence in brain sections of mice. We found that {**H_3_(Mor‐Cor)}** could cross the BBB as indicated by the presence of red fluorescence while absence of fluorescence suggested that {**H_3_(Cor)**} was unable to cross the BBB (**Figure** **9**A,B).

In addition, colocalization of the compound with MAP2 was quantitatively assessed and is presented in Figure 9C. These results suggest that the morpholine moiety aids in transporting {**H_3_(Mor‐Cor)**} across the BBB to improve the efficacy of the metallocorrole, as earlier studies have shown that metallocorroles are unable to cross the BBB.^[^ ^84^ ^]^

### 2.8 Neuroprotection in AD Transgenic Mice

**Figure 10:**
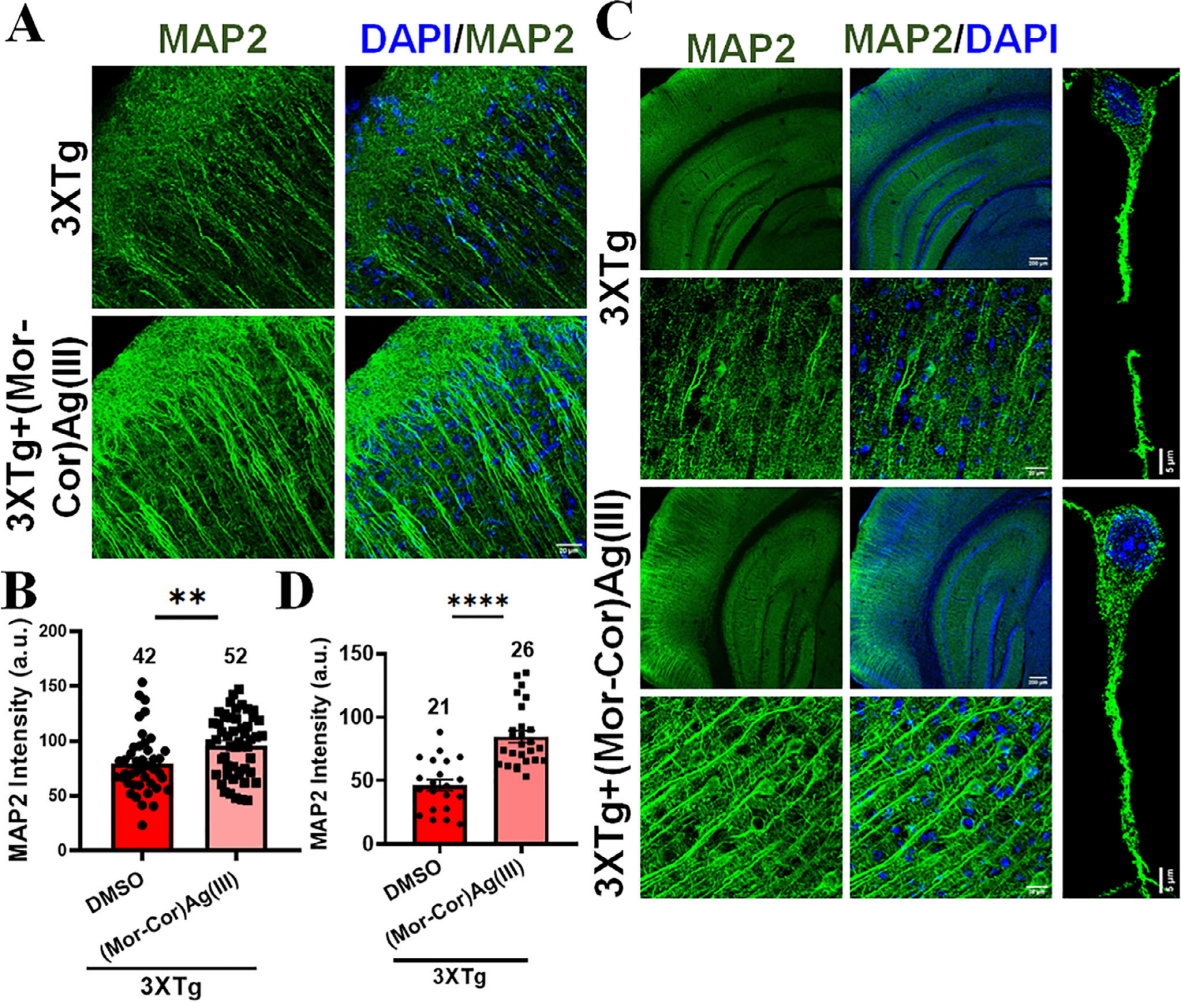
**(Mor‐Cor)Ag(III)** treatment enhances MAP2 immunofluorescence intensity in 3×Tg mice, indicating improved neuronal health in both short‐ and long‐term treatments: A) Representative images of brain sections from 3×Tg mice after short‐term (24 h) treatment with **(Mor‐Cor)Ag(III)** via tail vein injection, showing increased MAP2 immunostaining compared to control. Enhanced immunoreactivity suggests improved neuronal microtubule stability; B) Quantification of MAP2 fluorescence intensity confirms a significant increase in the compound‐treated group after 24 h (**p < 0.01); C) Representative images from the 7‐day treatment cohort, illustrating sustained enhancement of MAP2 immunostaining; D) Quantitative analysis shows a significant increase in MAP2 fluorescence intensity following chronic (7‐day) administration of **(Mor‐Cor)Ag(III)** compared to treated controls (*p < 0.05). Data are expressed as mean ± SEM. Statistical significance was determined by unpaired t‐test. Data acquired from 3 animals per group.

To test further the effect of **(Mor‐Cor)Ag(III)** we treated the triple transgenic (3×Tg) AD mouse model of 3–6 months age for 24 h. This transgenic mouse model of AD shows early neurodegeneration in the presence of abnormal soluble Aβ and tau proteins before the development of amyloid plaques and tau tangles.^[^ ^85^ ^]^ Therefore, this mouse model will help us in analysing the effect of soluble oligomeric Aβ, similar to our in vitro conditions. By quantifying the microtubule‐associated protein 2 (MAP2) intensity by confocal immunofluorescence microscopy in cortex of the treated mice, we observed a significant increase in dendritic density in **(Mor‐Cor)Ag(III)** treated mice compared to vehicle suggesting that **(Mor‐Cor)Ag(III)** has a neuroprotective effect and can alleviate AD pathogenesis (**Figure** **10**A,B). Chronic daily intravenous administration of **(Mor‐Cor)Ag(III)** for seven consecutive days significantly enhanced MAP2 immunoreactivity in the cortex of 6‐month‐old 3×Tg‐AD mice relative to DMSO vehicle‐treated controls (Figure 10C,D). These in vivo results suggest an improvement in neuronal cytoarchitecture and a reduction in cortical neurodegenerative alterations associated with Alzheimer's disease following **(Mor‐Cor)Ag(III)** treatment. Our ex vivo studies and studies conducted on 3×Tg mice indicate that **(Mor‐Cor)Ag(III)** could be developed as a therapeutic agent to treat AD‐related neurodegeneration. With only a few FDA (Food and Drug Administration) approved drugs for AD treatment, and given their limited efficacy,^[^ ^86^ ^]^ there is a pressing need for novel drug candidates.

### 2.9 **(Mor‐Cor)Ag(III)** Reduces Aβ42 Deposition in 3×Tg‐AD Mice

**Figure 11:**
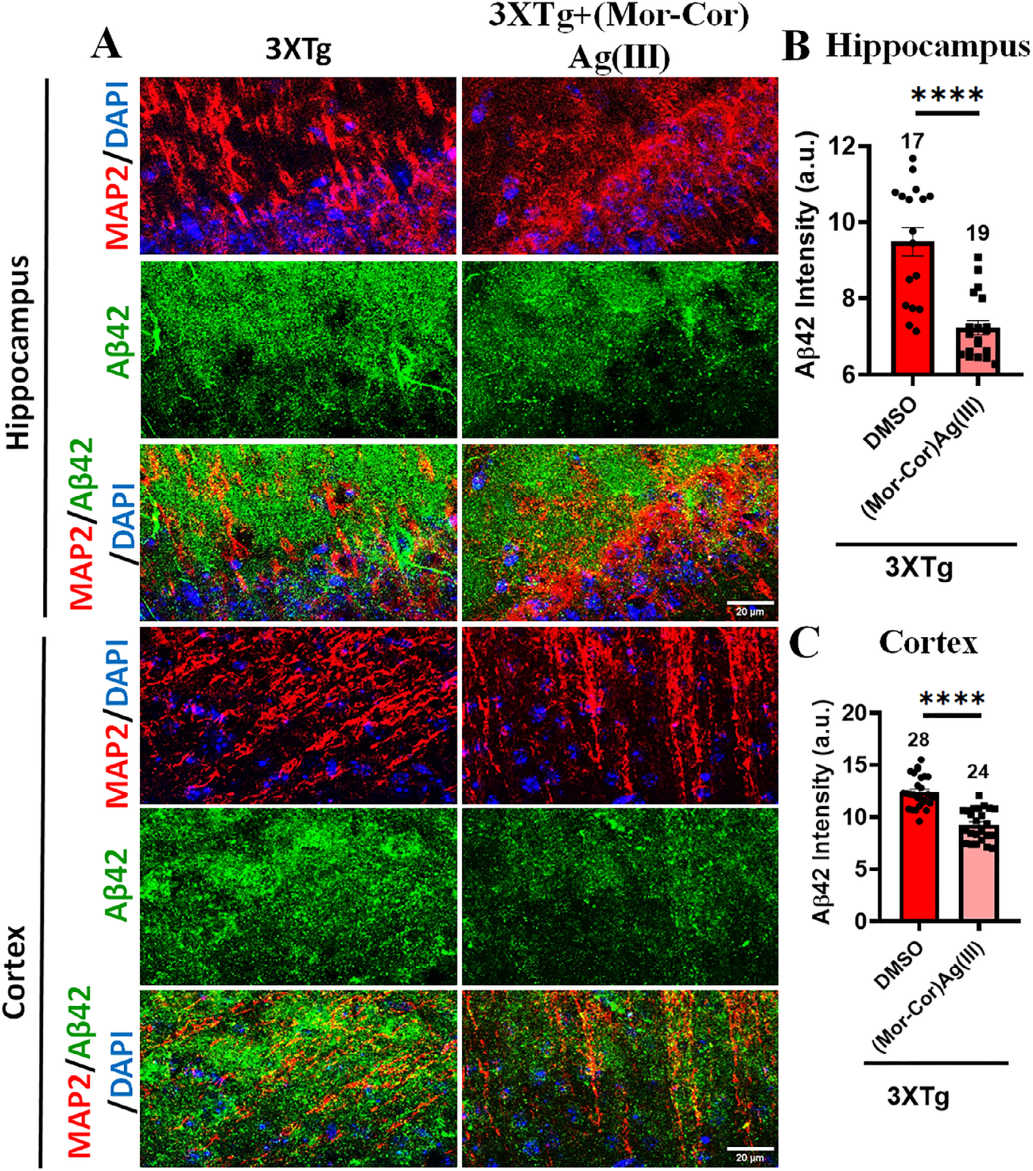
Chronic **(Mor‐Cor)Ag(III)** treatment reduces Aβ42 accumulation in 3×Tg‐AD mouse brain. A) Representative immunofluorescence images showing reduced Aβ_42_ immunostaining in both cortical and hippocampal regions following 7‐day daily tail vein injections, compared to vehicle‐treated controls. B) Quantitative analysis confirms a significant decrease in Aβ42 signal intensity in the hippocampus of treated animals, indicating enhanced Aβ42 clearance. C) Similar analysis reveals a significant reduction in cortical Aβ42 levels in the treated group. Data represent mean ± SEM; statistical significance was determined by unpaired *t*‐test (****p < 0.0001). Data were obtained from n = 3 animals per group.

Chronic systemic administration of (**Mor‐Cor)Ag(III)** (daily tail vein injections for 7 days) resulted in a significant reduction in Aβ42 aggregation, as determined by immunostaining in both the hippocampus and cortex of 6‐month‐old 3×Tg‐AD mice (**Figure** **11**A–C). This observation is consistent with established therapeutic approaches targeting amyloid pathology, wherein metal‐chelating agents and anti‐aggregation compounds inhibit Aβ fibrillization and mitigate associated neurotoxicity.^[^ ^87^ ^]^ The rapid decline in Aβ42 deposition observed within 1 week may be attributed to a reduction in reactive oxygen species (ROS) levels, as ROS have been previously implicated in promoting Aβ production and aggregation.^[^ ^88^, ^89^ ^]^ Accordingly, the therapeutic effect of (**Mor‐Cor)Ag(III)** may be mediated by its capacity to suppress ROS, thereby disrupting Aβ42 accumulation.

### 2.10 Plasma Pharmacokinetics of **(Mor‐Cor)Ag(III)** Following Tail Vein Injection

Quantitative analysis of **(Mor‐Cor)Ag(III)** concentrations in plasma was conducted using ultra‐high‐performance liquid chromatography (UHPLC), calibrated against a standard curve prepared with known concentrations (30, 60, 90, and 120 µM). The calibration curve exhibited excellent linearity, confirming the method's reliability for accurate quantification of **(Mor‐Cor)Ag(III)** in biological samples. Measured concentrations for the standards: 30 µM (39.637 µg mL^−1^), 60 µM (46.198 µg mL^−1^), 90 µM (88.694 µg mL^−1^), and 120 µM (125.471 µg mL^−1^)‐were in close agreement with expected values, validating the robustness and precision of the assay. Plasma samples collected at multiple time points following intravenous administration revealed stable systemic levels of **(Mor‐Cor)Ag(III)** during the initial post‐injection phase. At 30 min, the compound concentration averaged 37.2 µg mL^−1^ across technical replicates, indicating efficient bioavailability. Similar concentrations were observed at 1 h (≈36.7 µg mL^−1^), 2 h (≈36.0 µg mL^−1^), and 3 h (≈36.8 µg mL^−1^), suggesting sustained circulation with minimal early‐phase clearance. However, by 6 h post‐injection, the compound was below the detection limit, indicating a rapid terminal elimination phase (Figure S27A,B and Table S6, Supporting Information). Collectively, these data demonstrate that **(Mor‐Cor)Ag(III)** remains bioavailable in plasma for up to 3 h, followed by sharp systemic clearance.

## 3 Conclusions

With the aging global population, developing treatments for central nervous system (CNS) disorders like Alzheimer's is crucial. In this study, we synthesized a new Ag(III) corrole with a morpholino group **{** **(Mor‐Cor)Ag(III)}**, which demonstrated superior effectiveness in mitigating Aβ42 oligomer toxicity and reactive oxygen species (ROS) compared to a similar compound without the morpholino group. The FB corrole with a morpholino group {**H_3_(Mor‐Cor)}** was synthesized and characterized using various spectroscopic techniques. The silver metalation of **H_3_(Mor‐Cor)** yielded **(Mor‐Cor)Ag(III)**, which showed distinct spectroscopic changes compared to **H_3_(Mor‐Cor)**. Single crystal structure analysis of **(Mor‐Cor)Ag(III)** revealed a square planar geometry with specific Ag‐N bond distances and bite angles, consistent with previously reported **(Cor)Ag(III)** complexes. The crystallographic analysis showed various structural distortions and identified non‐covalent interactions forming a 2D porous supramolecular structure with solvent‐accessible voids. This study demonstrates that **(Mor‐Cor)Ag(III)** provides superior neuroprotection against Aβ42‐related neurotoxicity compared to **(Cor)Ag(III)**. While metallocorroles are known to protect neurons by decomposing ROS, the addition of a morpholine moiety to the metallocorrole enhances its ability to reduce ROS levels and provide better neuroprotection. The redox potentials of the silver(III) complexes suggest a potential mechanism for their activity, similar to iron(III) and manganese(III) corrole complexes. The morpholine moiety might enhance the compound's effectiveness through improved ROS reduction, molecular interactions, or altered pharmacological properties. Our results suggest it also facilitates crossing the blood‐brain barrier (BBB). Moreover, we demonstrate that in vivo administration of **(Mor‐Cor)Ag(III)** confers neuroprotection to both cortical and hippocampal neurons in 3×Tg‐AD mice following acute (24‐h) and chronic (7‐day) treatment regimens. Notably, treatment with **(Mor‐Cor)Ag(III)** significantly reduces Aβ42 levels in both brain regions, consistent with previous studies indicating that suppression of reactive oxygen species (ROS) can attenuate Aβ42 aggregation, thereby supporting the therapeutic relevance of **(Mor‐Cor)Ag(III)** in Alzheimer's disease.^[^ ^90^ ^]^ Furthermore, **(Mor‐Cor)Ag(III)** effectively diminishes neuronal hyperactivity, an early and critical pathological hallmark of AD. Given that elevated ROS levels are closely associated with neuronal hyperexcitability—and that reducing ROS has been shown to restore normal neuronal activity—**(Mor‐Cor)Ag(III)**’s ability to lower ROS levels likely underpins its capacity to normalize neuronal excitability.^[^ ^91^ ^]^ These findings collectively suggest that **(Mor‐Cor)Ag(III)** holds considerable promise as a therapeutic candidate for Alzheimer's disease (AD), owing to its capacity to counteract Aβ42 oligomer‐induced neurodegeneration. In light of the limited efficacy of currently approved AD treatments, the development of novel agents such as **(Mor‐Cor)Ag(III)** is of critical importance to advancing disease‐modifying therapeutic strategies.

## 4 Experimental Section

### Materials

The precursors, including pyrrole, *p*‐chloranil, Silver acetate Ag(OAc), Potassium iodide (KI), 4‐(2‐Chloroethyl)morpholine, 4‐cyanobenzaldehyde, and 4‐hydroxybenzaldehyde, were procured from Aldrich, USA. All other chemicals used were of reagent grade. Hexane and CH_2_Cl_2_ were distilled using KOH and CaH_2_, respectively. In spectroscopic and electrochemical studies, HPLC‐grade solvents were used. The precursors 4‐(2‐morpholinoethoxy)benzaldehyde and 4‐cyanophenyldipyrromethane were synthesized according to previously reported protocols.^[^ ^72^, ^73^ ^]^ 5,10,15‐Tris(4‐cyanophenyl)corrole, **H_3_(Cor)** and its silver(III) complex, **(Cor)Ag(III)**, were synthesized according to previously reported protocols.^[^ ^71^, ^72^ ^]^

### Physical Measurements

UV–vis spectral studies were performed on a Perkin‐Elmer LAMBDA‐750 spectrophotometer. The elemental analyses were carried out with a Euro EA elemental analyzer. Emission spectra were performed on an Edinburgh FLS920 spectrofluorometer equipped with a PMT980 for the visible and a Ge‐detector for emission in the NIR spectral region, using an optical cell of 1 cm path length. Emission quantum yields were calculated using coumarin (Φ_em_ = 0.54) as the reference in degassed CH_2_Cl_2_. FT‐IR spectra were recorded on a Perkin‐Elmer spectrophotometer with samples prepared as KBr pellets. The NMR measurements were carried out using a Bruker AVANCE 400 NMR spectrometer. Tetramethylsilane (TMS) is the internal standard. Electrospray mass spectra were recorded on a Bruker Micro TOF‐QII mass spectrometer. Cyclic voltammetry was measured using a CS350 electrochemical test system (China). A glassy carbon working electrode, a platinum wire as an auxiliary electrode, and an Ag‐AgCl reference electrode were used in a three‐electrode configuration. Tetrabutylammonium perchlorate (TBAP) was the supporting electrolyte (0.1 M), and the solution concentration was 10^−3^ M for the complex. The half‐wave potential E^0^_298_ was set equal to 0.5 (E_pa_ + E_pc_), where E_pa_ and E_pc_ are anodic and cathodic cyclic voltammetric peak potentials, respectively. The scan rate used was 100 mV s^−1^. The thermogravimetric analysis was performed using a Discovery TGA (TA Instruments‐Waters Lab) set up under nitrogen and oxygen atmospheres. The Brunauer‐Emmett‐Teller (BET) surface area of **(Mor‐Cor)Ag(III)** was measured using Quantachrome Instruments (Autosorb iQ) through an N_2_ adsorption‐desorption isotherm.

### Crystal Structure Determination

Single crystals of **(Mor‐Cor)Ag(III)** were grown by slow diffusion of solution of **(Mor‐Cor)Ag(III)** in CH_2_Cl_2_ with hexane, followed by slow evaporation under atmospheric conditions. The crystal data of **(Mor‐Cor)Ag(III)** was collected on a Rigaku Oxford diffractometer at 100 K. Selected data collection parameters, and other crystallographic results are summarized in Table S1, Supporting Information. All data were corrected for Lorentz polarization and absorption effects. The program package ShelxTL^[^ ^92^ ^]^ was used for structure solution and full‐matrix least‐squares refinement on F^2^. Hydrogen atoms were included in the refinement using the riding model. Contributions of H atoms for the water molecules were included but were not fixed. Disordered solvent molecules were taken out using the SQUEEZE^[^ ^93^ ^]^ command in PLATON. CCDC 2361178 contains the supplementary crystallographic data for **(Mor‐Cor)Ag(III)**. These data can be obtained free of charge via www.ccdc.cam.ac.uk/data_request/cif .

### Computational Methods

All computations were carried out using the Gaussian 06 software.^[^ ^94^ ^]^ Geometry optimizations for **H_3_** (**Mor‐Cor**) and **(Mor‐Cor)Ag(III)** were conducted at the B3LYP/6‐311G(d,p) level of theory. For the Ag atom, the LANL2DZ pseudopotential was employed.The TD‐DFT calculations were also performed at the B3LYP level with 6‐311G(d,p) basis sets, utilizing the Gaussian 06 software. A PCM solvent model was employed in the calculations, with DCM considered as the solvent.

### Synthesis

*For 4‐(2‐morpholinoethoxy)benzaldehyde*: 4‐(2‐morpholinoethoxy)benzaldehyde was prepared according to the published procedure.^[^ ^73^ ^]^ Anal. Calcd (found) for C_13_H_17_NO_3_: C, 66.36 (66.27); H, 7.28 (7.39); N, 5.95 (5.83). ^1^H NMR (400 MHz, CDCl_3_) δ 9.85 (s, 1H), 7.82 (d, *J* = 12 Hz, 2H), 6.99 (d, *J* = 8 Hz, 2H), 4.17 (t, *J* = 4 Hz, 2H), 3.71 (t, *J* = 4 Hz, 4H), 2.81 (t, *J* = 4 Hz, 2H), 2.56 (t, *J* = 4 Hz, 4H). ^13^C{^1^H} NMR (101 MHz, CDCl_3_) δ 190.92, 163.77, 132.06, 130.06, 114.88, 66.93, 66.21, 57.43, 54.14 (Figures S5 and S6, Supporting Information). Analytical data matches nicely with the earlier report.^[^ ^73^ ^]^

*For 4‐cyanophenyldipyrromethane*: 4‐cyanophenyldipyrromethane was prepared according to the published procedure.^[^ ^72^ ^]^ Anal. Calcd (found) for C_16_H_13_N_3_: C, 77.71 (77.64); H, 5.30 (5.49); N, 16.99 (16.87). ^1^H NMR (400 MHz, CDCl_3_) δ 8.03 (s, 2H), 7.62 (d, *J* = 8.0 Hz, 2H), 7.34 (d, *J* = 8.0 Hz, 2H), 6.76 (q, *J* = 2.3 Hz, 2H), 6.19 (d, *J* = 3.0 Hz, 2H), 5.88 (d, *J* = 3.3 Hz, 2H), 5.55 (s, 1H).^13^C{^1^H} NMR (101 MHz, CDCl_3_) δ 147.78, 132.51, 131.08, 129.29, 118.95, 118.02, 110.89, 108.82, 107.87, 44.09. Analytical data matches nicely with the earlier report.^[^ ^72^ ^]^

*Synthesis of [10‐{4‐(2‐morpholinoethoxy)phenyl}‐5,15‐bis‐(4‐cyanophenyl)corrole*, ***{H_3_(Mor‐Cor)}***: The free base corrole **H_3_** (**Mor‐Cor**), was synthesized following the previously reported Gryko methodology.^[^ ^72^ ^]^ Specifically, 4‐(2‐morpholinoethoxy)benzaldehyde (0.118 g, 0.5 mmol) and 4‐cyanophenyldipyrromethane (0.247 g, 1 mmol) were dissolved in a mixture of 50 mL of MeOH and 50 mL of water. Subsequently, 5 mL of HCl was added to the reaction mixture. The resulting mixture was stirred for 1 h. Following this, the mixture was extracted with CHCl_3_, and the organic layer was washed thrice with H_2_O, dried over anhydrous Na_2_SO_4_, filtered, and then diluted to 100 mL with CHCl_3_. Then, p‐chloranil (0.369 g, 1.5 mmol) was added to the solution, and the mixture was refluxed for 1 h. The solvent was then evaporated using a rotary evaporator, yielding a greenish‐black colored crude product. This crude product was subsequently purified using column chromatography on a silica gel (100–200 mesh) column, employing DCM and acetonitrile (20%) as the eluent.

Yield: 82 mg (0.116 mmol, 23%). Anal. Calcd (found) for C_45_H_35_N_7_O_2_ {**H_3_** (**Mor‐Cor**)}: C, 76.58 (76.48); H, 5.00 (5.13); N, 13.89 (13.98). UV–vis (CH_2_Cl_2_) λ_max_/nm (ε/M^−1^cm^−1^): 424 (104 000), 580 (17 000), 629 (12 000), 656 (11 000). ^1^H NMR (400 MHz, chloroform‐d) δ 8.95 (d, J = 4 Hz, 2H), 8.81 (d, J = 4 Hz, 2H), 8.63 (d, J = 4 Hz, 2H), 8.53 (d, J = 8 Hz, 2H), 8.46 (d, J = 4 Hz, 4H), 8.10 (d, J = 8 Hz, 4H), 8.06 (d, J = 8 Hz, 2H), 7.31 (d, J = 8 Hz, 2H), 4.41 (t, J = 4 Hz, 2H), 3.81 (t, J = 4 Hz, 4H), 3.01 (t, J = 4 Hz, 2H), 2.71 (t, J = 4 Hz, 4H) (Figure S7, Supporting Information). HRMS (ESI) *m/z*: [**M**+H]**^+^** Calcd for C_45_H_36_N_7_O_2_ 706.2930; Found 706.2998 (Figure S3, Supporting Information).

Synthesis of [10‐{4‐(2‐morpholinoethoxy)phenyl}‐5,15‐bis‐(4‐cyanophenyl)‐corrolatosilver(III), **(Mor‐Cor)Ag(III)**:

The silver(III)‐corrole complex; **(Mor‐Cor)Ag(III)** was prepared using a previously reported protocol.^[^ ^63^ ^]^ First, the free base corrole, **H_3_** (**Mor‐Cor**) (50 mg, 0.07 mmol), was dissolved in 30 mL of HPLC‐grade acetonitrile. Subsequently, 1 mL of triethylamine was added to the reaction mixture, and continuous stirring was maintained for 30 min. Following this, 150 mg of silver acetate Ag(OAc) was introduced into the reaction mixture, and continuous stirring at room temperature was carried out to facilitate complex formation. Excess silver acetate was removed by filtration, and the solvent was evaporated from the reaction mixture using a rotary evaporator. The resulting complex was further purified using column chromatography (silica gel 100–200 mesh, CH_2_Cl_2_/hexane). For recrystallization, a dichloromethane‐hexane solvent mixture (1:1, v/v) was utilized, yielding reddish‐brown crystals of the silver(III)‐corrole complex, **(Mor‐Cor)Ag(III)**.

Yield 15 mg (0.019 mmol, 27%). Anal. Calcd (found) for C_45_H_32_N_7_O_2_Ag {**(Mor‐Cor)Ag(III)**}: C, 66.67 (66.74); H, 3.98 (3.82); N, 12.09 (12.19). UV–vis (CH_2_Cl_2_) λ_max_/nm (ε/M^−1^cm^−1^): 427 (102 000), 588 (28 000). ^1^H NMR (400 MHz, chloroform‐d) δ 8.93 (d, J = 4 Hz, 2H), 8.82 (d, J = 4 Hz, 2H), 8.75 (d, J = 4 Hz, 2H), 8.50 (d, J = 8 Hz, 2H), 8.33 (d, J = 8 Hz, 4H), 8.13 (d, J = 8 Hz, 4H), 8.04 (d, J = 8 Hz, 2H), 7.35 (d, J = 8 Hz, 2H), 4.43 (t, J = 4 Hz, 2H), 3.86 (t, J = 4 Hz, 4H), 3.03 (t, J = 4 Hz, 2H), 2.76 (t, J = 4 Hz, 4H) (Figure S8, Supporting Information). ^13^C{^1^H} NMR (101 MHz, CDCl_3_) δ: 158.8, 145.1, 137.9, 135.6, 135.2, 133.6, 133.3, 131.6, 130.1, 129.5, 128.6, 126.5, 119.4, 119.3, 118.6, 115.6, 114.7, 113.8, 111.6, 67.1, 66.2, 57.9, 54.3. HRMS (ESI) *m/z*: [**M**+H]**^+^** Calcd for C_45_H_33_N_7_O_2_Ag 810.1747; Found 810.1754 (Figure S4, Supporting Information).

### Animal Studies

The experimental protocols were performed in accordance with CPCSEA regulations (Registration No. 1634/GO/ReRcBiBt/S/12/CPCSEA, DoR‐ 16.05.2023) and were approved by the Institutional Animal Ethics Committee (IAEC) at the National Institute of Science Education and Research (NISER), Bhubaneswar, Odisha, India (Ethical Approval No: NISER/SBS/AH‐322). Primary neuron–astrocyte co‐cultures were prepared from postnatal day 0–1 (P0–P1) Sprague‐Dawley rat pups. Blood–brain barrier penetration studies were performed using C57BL/6 mice. Therapeutic efficacy was assessed in the triple‐transgenic Alzheimer's disease mouse model (3×Tg‐AD; APPSwe, tauP301L; MMRRC ID: 034830‐JAX‐004807).

### Cell Culture

Primary dissociated cortical neuron‐astrocyte cultures were prepared from P0‐1 day old Sprague‐Dawley rat. The brains were isolated in ice cold calcium magnesium free Hanks’ Balanced Salt Solution (HBSS) containing 10 mM glucose and HEPES solution (Sigma). Cortical tissue was separated in HBSS and digested with 0.25% trypsin‐EDTA (Gibco) and 150 units/ml DNAse (Sigma) for 15 min at 37 °C. Trypsin was inactivated by using 10% foetal bovine serum (FBS)(Gibco). The cells were subsequently mechanically dissociated and centrifuged at 1000 rpm for 5 min at 4 °;C. The pellet was resuspended in Neurobasal media. The neuron astrocyte media consists of neurobasal‐A medium (Gibco) supplemented with 10% FBS (Gibco), 1% Glutamax (Gibco), 1% Anti‐anti (Gibco) and 2% B27 supplement (Gibco). The cells were plated on 0.1 mg/ml Poly‐D‐lysine coated coverslips. The cultures were transferred to a controlled environment where humidity levels were maintained with 5% CO_2_ at 37 °C. The culture medium was changed by replacing half of it at the fourth division, followed by subsequent changes every 3 days thereafter. Experiments were performed on day 11–12. The immortalized mouse hippocampal cell line (HT22) cells were cultured on surfaces pre‐coated with poly‐D‐lysine and for adherence and optimal growth conditions. Cells were used at passage numbers 3–6. The cells were incubated in Dulbecco's modified Eagle's medium/F12 (1:1) (Gibco) supplemented with 10% fetal bovine serum (Gibco) and 1% Anti‐anti (Gibco) for 24 h prior to any treatment to induce differentiation. Human embryonic kidney (HEK293) cells were cultured in Dulbecco's modified Eagle's medium (high glucose DMEM) (Sigma), supplemented with 10% fetal bovine serum (Gibco) and 1% antibiotic–antimycotic (Gibco), in a humidified incubator with 5% CO_2_ atmosphere. The HT22 cell lines and the primary neuron astrocyte cultures were regularly tested for mycoplasma contamination using MycoAlert Plus kit (Lonza) and tested negative in each instance.

### Preparation of Aβ Oligomers and Compounds

Beta‐Amyloid (1‐42) monomers (Anaspec) were used to obtain soluble oligomers with the final concentration of 5 µM by previously described protocol^[^ ^9^, ^95^, ^96^, ^97^, ^98^, ^99^, ^100^, ^101^, ^102^, ^103^, ^104^, ^105^, ^106^ ^]^1 ml of 1 mM solutions of **(Mor‐Cor)Ag(III)** and **(Cor)Ag(III)** compounds were prepared using DMSO (Sigma) as the solvent. To ensure a homogeneous solution, bath sonication was employed. Subsequently, 10^−^⁵ M solutions were prepared by diluting the 1 mM solutions of **(Mor‐Cor)Ag(III)** and **(Cor)Ag(III)**. For biological applications, 1 µM, 2 µM, and 6 µM solutions of each compound concentration were prepared in buffer solution by further diluting the 10^−5^ M solutions of **(Mor‐Cor)Ag(III)** and **(Cor)Ag(III)**.

### Immunocytochemistry

After 24 h of treatment cells were washed with 1× PBS one time. Then fixed with 4% paraformaldehyde for 30 min. After fixation three times washed with PBS. Next blocking and permeabilization solution (3% BSA and 0.3% Triton‐X100 in PBS) was added and incubated for 45 min and three times washed with PBST (0.1% Triton‐X100 in PBS). Primary antibodies of rabbit IgG MAP2 (1:150, cell signalling technology), mouse IgG GFAP (1:300, Sigma) in PBST were added to the cells and incubated overnight at 4 °C. Then cells were washed one time with PBST and twice with PBS. Cells were incubated with secondary antibodies (cell signalling technology) anti rabbit Alexa Fluor 488 (1:1000) and anti‐mouse Alexa Fluor 555 (1:1000) in PBS for 2 h in a dark place at room temperature. Then cells were washed with PBS three times. Cells were mounted using Prolong Gold with DAPI (Invitrogen). Image acquisition was done using Leica DM i8 confocal microscope in 63× objective. Neurite length was calculated using a simple neurite tracer (SNT) plugin in Fiji. Fragmented neurites were identified by structural discontinuities (breaks or swellings). Neurite length was measured from the nuclear center to the first fragmentation point, with intact segments serving as indicators of neuronal health. **Mean neurite length = (Σ neurite lengths) / (Total number of neurite segments or paths)**. To calculate the GFAP intensity per astrocyte, the mean GFAP fluorescence intensity of each image was measured and divided by the number of astrocyte nuclei. The number of astrocyte nuclei was counted manually.

### Cell Death Assay

Cell death was assessed through the application of propidium iodide (Invitrogen) staining. Cells were treated with the compounds and Aβ42 oligomers together for 24 h. Following treatment, cortical neuron astrocyte cultures underwent incubation at 37 °C for 1 h in HBSS with a 10 µM propidium iodide (PI). HT22 cell line was incubated with 10 µM PI for 15 min at 37 °C. Following this, the cultures were fixed in 4% paraformaldehyde, washed with 1× PBS both before and after fixation and finally mounted using Prolong Gold (Invitrogen). Images were taken on the Zeiss fluorescence microscope in 20× objective. Post‐acquisition analysis of PI‐staining was performed by counting PI‐stained nuclei from randomly selected areas of each coverslip.

### CellROX Deep Red Assay

After 24 h of treatment with Aβ42 oligomers, primary cortical neuron–astrocyte co‐cultures were incubated with 10 µM CellROX Deep Red (Invitrogen) in HBSS for 30 min at 37 °C. Then cells were washed with PBS three times followed by fixation with 4% paraformaldehyde. After fixation again three times washed with PBS and mounted with Prolong Gold (Invitrogen). Images were taken on the Zeiss fluorescence microscope in 20× objective. Random areas of each coverslip were imaged. To quantify cell ROX intensity, mean fluorescence intensity of each field acquired was measured. All fluorescence intensities were normalized by the number of nuclei in that field.

### Calcium Imaging

The 2× Fluo‐4 AM Direct calcium reagent (Invitrogen) was reconstituted in 10 mL of assay buffer containing 200 µL of 250 mM probenecid (to inhibit organic anion transporters). The solution was then diluted 1:1 with culture medium to yield a 1× working concentration. Treated cells were loaded with the 1× Fluo‐4 AM Direct solution in glass‐bottom dishes and incubated for 30 min at 37 °C, followed by a 30‐min equilibration at room temperature. Before imaging, the dye solution was replaced with fresh assay buffer. Time‐lapse fluorescence imaging was performed for 2 min at 33 fps using a Zeiss fluorescence microscope equipped with an Axiocam 702 monochromatic camera. Neuronal ROIs were manually selected in FIJI/ImageJ, and fluorescence traces (F) were extracted using Python scripts. Baseline (F_0_) was defined as the mean of the 10 lowest fluorescence values. Calcium transients (ΔF/F_0_ = (F−F_0_)/F_0_) were detected via automated peak detection (minimum prominence/height/distance criteria).

### Compound Administration in Mice

A total of 200 µg of **(Mor‐Cor)Ag(III)** was dissolved in 100 µL of Hank's Balanced Salt Solution (HBSS) containing 2% DMSO. C57 mice were anesthetized with a ketamine‐xylazine mixture. Tail hair was removed using a scalpel blade and the area was disinfected with an ethanol spray. To enhance vein visibility, the tail was immersed in warm water in a 15 mL centrifuge tube. A 25 G needle was inserted at a shallow angle nearly parallel to the vein, and 100 µL of the HBSS/**(Mor‐Cor)Ag(III)**/DMSO solution was slowly injected. This corresponds to ≈6–8 µg of compound per g of bodyweight. After the injection, the needle was carefully removed, and the animals were moved to a recovery area. The fluorescent compound similar in structure to **(Mor‐Cor)Ag(III)** and **(Cor)Ag(III)** named **H_3_(Mor‐Cor)** and **H_3_(Cor),** respectively (200 µg in 100 µL; emission 683nM), was administered via tail vein injection to C57 mice for 2 h. The preparation of compound solution and injection were as stated above. For assessing the effect of the compound in AD transgenic mice, **(Mor‐Cor)Ag(III)** (200 µg in 100 µL) was injected into 3–6 month old 3×Tg mice, and the same post‐injection protocol was followed. Acute treatment for 24 h and chronic treatment (daily injections for 7 days) was performed. Brain extraction and processing followed an identical protocol. The body weight of the 3×Tg mice was measured before and after compound injection and was found to be unchanged.

### Mass Spectrometry Analysis

Following a 2‐h post‐injection incubation period, the animals were re‐anesthetized. Transcardial perfusion was performed using Phosphate‐Buffered Saline (PBS). The entire brain was then isolated and immersed in 1 mL of 100% DMSO. The brain tissue was thoroughly triturated, and the resulting mixture was centrifuged at 20 000 rcf for 20 min. The supernatant from this centrifugation was collected for mass spectrometry analysis (Figure 8). The experiment was repeated three times and the spectrum has been shown in the supplementary figures.

### Cryosectioning and Immunohistochemistry

2 h’ post‐injection, transcardial perfusion was performed with 1× PBS followed by 4% paraformaldehyde (PFA), after which brains were extracted and fixed in 4% PFA for 24 h or 7 days. The fixed brains were then cryoprotected using a sucrose gradient (15%, 20%, and 30%) for 24 h at each concentration, embedded in cryo‐freezing medium (Leica), and sectioned into coronal slices at 30 µm thickness. For immunostaining, sections were permeabilized and blocked with 5% BSA and 0.5% Triton X‐100 for 45 min at room temperature. They were then incubated overnight at 4 °C with rabbit anti‐MAP2 (1:150, Cell Signaling Technology) or Chicken IgG MAP2 (1:1000, Invitrogen) and rabbit IgG Aβ42 (1:200, Invitrogen) antibody diluted in 5% BSA. After washing, Alexa 488‐conjugated anti‐rabbit IgG (1:1000, Cell Signaling Technology) was used as the secondary antibody. Sections were mounted using ProLong Gold (Invitrogen). Image acquisition for the fluorescent compound was performed using a Zeiss fluorescent microscope with a 20× objective, while imaging of the non‐fluorescent compound‐injected brain sections was conducted using a Leica DM i8 confocal microscope with a 63× objective. Zoomed images were captured using the Zeiss Elyra 7 super‐resolution microscope. Colocalization between MAP2 and the fluorescent compound was assessed using the Manders’ coefficient.

### Blood Clearance Assay of **(Mor‐Cor)Ag(III)**

Experiments were conducted in C57BL/6 mice (12 weeks old, 25–30 g). Animals received a tail vein injection of **(Mor‐Cor)Ag(III)** at a dose of 200 µg. Blood samples were collected at predetermined time points (0.5, 1, 2, 3, 6, 12, and 24 h) into 0.5 ml microcentrifuge tubes pretreated with EDTA to prevent coagulation. After allowing the tubes to remain undisturbed at room temperature, samples were centrifuged at 3000 × g for 10 min at 4 °C to isolate plasma. The plasma was carefully transferred under ice‐cold conditions and stored in small aliquots at −80 °C until analysis. For UHPLC analysis, 100 µL of plasma was mixed with ice‐cold acetonitrile (ACN) in a 1:3 ratio (plasma:ACN), vortexed for 2–3 min to precipitate proteins, and centrifuged at 16 600 × g for 5 min at 4 °C. The resulting supernatant was stored at −80 °C until further use. Standard samples were prepared by serially diluting the **(Mor‐Cor)Ag(III)** stock solution with blank plasma, maintaining equal volumes of plasma across all standards. The standard concentrations used were 30, 60, 90, and 120 µM. The UHPLC analysis was performed on a Shimadzu LC system comprising a CBM‐40lite system controller, LC‐40D XS pump, SIL‐40C XSi autosampler, CTO‐40S column oven, and SPD‐M40S photodiode array detector. The autosampler temperature was set to 10 °C. Separation was achieved on a Shim‐pack Scepter C18‐120 column (1.9 µm, 2.1 mm × 100 mm) maintained at 40 °C using a mobile phase of double‐distilled water (solvent A) and acetonitrile (solvent B) in an 11:89 (v/v) ratio. The flow rate was set at 0.3 mL min^−1^ with a total run time of 13 min per sample. An injection volume of 1 µL was used, and detection was performed at 427 nm.

### Statistical Analysis

All image analysis was done using Fiji.^[^ ^107^ ^]^ The data are presented as the mean ± SEM, based on a minimum of 3–4 independent experiments or biological replicates. Intensity was quantified from 5–7 randomly selected fields per coverslip, with values normalized to the number of nuclei per field. For experiments done on primary cell cultures, all data were collected from at least 4–5 animals/ experiment. In all experiments, groups were randomly assigned, and both image acquisition and data analysis were carried out by an experimenter blinded to the experimental conditions. Statistical analyses were done using GraphPad Prism 8.0.1 (GraphPad Software, San Diego, CA, USA). For comparisons between two groups, an unpaired *t*‐test was used. For multiple comparisons, ANOVA followed by a post hoc test, specifically Sidak's multiple comparison test, was used for all analyses. *p* values < 0.05 were regarded as significant.

## Conflict of Interest

The authors declare no conflict of interest.

## Author Contributions

A.T. and T.M. contributed equally as authors. S.G. and S.K. are corresponding authors and contributed equally to this work. Investigation, Methodology, Data curation, Formal analysis, writing – original draft preparation‐A.T., T.M., T.P., J.M., R.C., K.T., S.G., S.K. Conceptualization, Writing – review and editing, Funding acquisition, and Supervision‐ S.G. and S.K.

## Supporting information



Supporting Information

Supplemental Video 1

Supplemental Video 2

Supplemental Video 3

Supplemental Video 4

Supplemental DataFile

## Data Availability

The data that support the findings of this study are available in the supplementary material of this article.
