# Supplementary material for: A Blood‐Brain Barrier‐Penetrant Ag(III) Corrole Compound Rescues Alzheimer's Disease Pathology by Targeting Aβ42‐Induced Oxidative Stress
Source: Adv Sci (Weinh). 2026 Jan 8;13(8):e15462. doi: 10.1002/advs.202515462 (PMC12884804; doi:10.1002/advs.202515462)
Supplement: Supplementary file 1 — Supporting Information [file ADVS-13-e15462-s006.docx]

**SUPPORTING INFORMATION**

**A Blood-Brain Barrier-Penetrant Ag(III) Corrole Compound Rescues Alzheimer’s Disease Pathology by Targeting Aβ42-Induced Oxidative Stress**

Arup Tarai,^[a],[b],#^ Tuhina Mitra,^[c],#^ Tanmoy Pain,^[a]^ Jyotiprakash Mallick,^[a]^ Rwiddhi Chakraborty,^[a]^ Kallam Tejaswi,^[c]^ Swagata Ghatak,*^,[c]^ and Sanjib Kar*^,[a]^

*^[a]^School of Chemical Sciences, National Institute of Science Education and Research (NISER), Bhubaneswar – 752050, India, and Homi Bhabha National Institute, Training School Complex, Anushakti Nagar, Mumbai, 400 094, India. Email:* [*sanjib@niser.ac.in*](mailto:sanjib@niser.ac.in)

*^[b]^Department of Chemistry, Mahila Mahavidyalaya (MMV), Banaras Hindu University (BHU), Varanasi 221005, India.*

*^[c]^School of Biological Sciences, National Institute of Science Education and Research (NISER), Bhubaneswar – 752050, India, and Homi Bhabha National Institute, Training School Complex, Anushakti Nagar, Mumbai, 400 094, India. Email:* [*swagata@niser.ac.in*](mailto:swagata@niser.ac.in)

*^#^These authors have contributed equally.*

**Table S1** Crystallographic data for **(Mor-Cor)Ag(III)**.

**Table S2** Some important hydrogen bond parameters of **(Mor-Cor)Ag(III)**.

**Table S3** Most important photophysical data of compounds; **H_3_(Mor-Cor)** and **(Mor-Cor)Ag(III)** in CH_2_Cl_2_ solution at 298 K.

**Table S4** TD-DFT Calculated Electronic Transitions for **H_3_(Mor-Cor)**.

**Table S5** TD-DFT Calculated Electronic Transitions for **(Mor-Cor)Ag(III)**.

**Table S6** UHPLC quantification of **(Mor-Cor)Ag(III)** in plasma using a standard calibration curve. Consistent concentrations were detected up to 3 hours; no signal was observed at 6 hours, indicating clearance.

**Appendix 1 Optimized Cartesian Co-ordinates of H_3_(Mor-Cor).**

**Appendix 2 Optimized Cartesian Co-ordinates of (Mor-Cor)Ag(III).**

**Figure S1** FT IR spectra of **H_3_(Mor-Cor)**.

**Figure S2** FT IR spectra of **(Mor-Cor)Ag(III)**.

**Figure S3** ESI-MS spectrum of **H_3_(Mor-Cor)** in CH_3_CN shows the measured spectrum with an isotopic distribution pattern.

**Figure S4** ESI-MS spectrum of **(Mor-Cor)Ag(III)** in CH_3_CN shows the measured spectrum with an isotopic distribution pattern.

**Figure S5** ^1^H NMR (CDCl_3_, 400 MHz) spectrum of 4-(2-morpholinoethoxy)benzaldehyde.

**Figure S6** ^13^C{^1^H} NMR (CDCl_3_, 101 MHz) spectrum of 4-(2-morpholinoethoxy)benzaldehyde.

**Figure S7** ^1^H NMR (CDCl_3_, 400 MHz) spectrum of **H_3_(Mor-Cor)**.

**Figure S8** ^1^H NMR (CDCl_3_, 400 MHz) spectrum of **(Mor-Cor)Ag(III)**.

**Figure S9** ^13^C{^1^H} NMR (CDCl_3_, 101 MHz) spectrum of **(Mor-Cor)Ag(III)**.

**Figure S10** (a) DFT-optimized geometry of **H_3_(Mor-Cor)** using the 6-311G (d, p) basis set and (b) linear display of non-planar distortions for **H_3_(Mor-Cor)**.

**Figure S11** (a) DFT-optimized geometry of **(Mor-Cor)Ag(III)** using the 6-311G (d, p) basis set and (b) linear display of non-planar distortions for **(Mor-Cor)Ag(III)**.

**Figure S12** Selected bond distances of **(Mor-Cor)Ag(III)** from (a) Crystal structure and (b) DFT optimized structure.

**Figure S13** Chain-like supramolecular assembly in **(Mor-Cor)Ag(III)**.

**Figure S14** C-H^…^π interactions in **(Mor-Cor)Ag(III)**.

**Figure S15** TGA thermogram of **(Mor-Cor)Ag(III)**.

**Figure S16** TD-DFT-based electronic absorption spectra of **H_3_(Mor-Cor)**.

**Figure S17** Selected frontier MOs with orbital energies of **H_3_(Mor-Cor)**.

**Figure S18** TD-DFT-based electronic absorption spectra of **(Mor-Cor)Ag(III)**.

**Figure S19** Selected frontier MOs with orbital energies of **(Mor-Cor)Ag(III)**.

**Figure S20** Aβ42 exposed HT-22 hippocampal cells manifest decreased cell death on treatment with **(Mor-Cor)Ag(III)** at various concentrations (A) Quantification of cell death to determine toxicity of the compound; (B) Quantification of cell death after applying Aβ42 with different concentration of compounds. Data are mean ± SEM. Statistical significance analysed by Kruskal Walis test with posthoc test, Dunn’s multiple comparison test. Sample size listed above bar graphs (*p < 0.05; **p < 0.01; ***p < 0.001;  **** p < 0.0001) acquired from 3 independent experiments.

**Figure S21** Aβ42- exposed neurons and astrocytes show decreased pathological characteristics on treatment with **(Mor-Cor)Ag(III)**. Representative images of neurons expressing MAP2 (green) and astrocytes expresing GFAP (orange) in 20X magnification.

**Figure S22** No Significant Effect of Compound-Only Treatment on Total Neurite Length (A) Representative images showing no change in total neurite length in the compound-only control group. (B) Quantification of total neurite length, confirming the absence of changes in the compound-only control condition. Data are presented as mean ± SEM. Statistical significance was analyzed using ANOVA followed by Sidak’s multiple comparisons test between selected pairs (P > 0.05).

**Figure S23** No Significant Change in GFAP Intensity Following Compound-Only Treatment. (A) Representative images showing no change in GFAP intensity in the compound-only control group. (B) Quantification of GFAP intensity confirming the absence of changes in the compound-only control condition. Data are mean ± SEM. Statistical significance analyzed by ANOVA with Sidak’s multiple comparison test between selected pairs (P > 0.05).

**Figure S24** Biological replicates of ESI-MS spectra confirming brain accumulation of **(Mor-Cor)Ag(III).** Spectra were obtained from brain samples isolated after intravenous tail vein injection of **(Mor-Cor)Ag(III)**, followed by extraction in CH₃CN. Each spectrum shows the selected mass region corresponding to the compound’s expected mass, validating its presence in multiple animals. Replicates shown in A and B.

**Figure S25 (a)**The ESI-MS spectrum of the sample in CH_3_CN displays the measured spectrum of the full region, observed after intravenous administration of **(Mor-Cor)Ag(III)** into the mouse *via* tail vein injection and subsequent brain isolation.

**Figure S25 (b)**The ESI-MS spectrum of the sample in CH_3_CN displays the measured spectrum of the selected region, observed after intravenous administration of **(Mor-Cor)Ag(III)** into the mouse *via* tail vein injection and subsequent brain isolation.

**Figure S26 (a)**The ESI-MS spectrum of the sample in CH_3_CN displays the measured spectrum for the entire region, observed without administering **(Mor-Cor)Ag(III)** to the mouse.

**Figure S26 (b)**The ESI-MS spectrum of the sample in CH_3_CN displays the measured spectrum for the selected region, observed without administering **(Mor-Cor)Ag(III)** to the mouse.

**Figure S27** Plasma pharmacokinetics of **(Mor-Cor)Ag(III)** following tail vein injection. Quantitative UHPLC analysis of plasma samples collected at various time points (0.5 to 6 hours) post-injection shows that **(Mor-Cor)Ag(III)** remains stable in circulation up to 3 hours, with concentrations ranging from ~36–37 µg/mL. A sharp decline is observed at 6 hours, indicating rapid clearance of the compound from systemic circulation.

**Video S1** Representative live-cell calcium imaging videos showing calcium transients in control neurons exhibiting regular calcium activity. Time stamps are shown in seconds. Scale bars: 100 μm.

**Video S2**  Representative live-cell calcium imaging videos showing calcium transients in Aβ42-treated neurons displaying increased calcium transients and hyperexcitability. Time stamps are shown in seconds. Scale bars: 100 μm.

**Video S3**  Representative live-cell calcium imaging videos showing calcium transients in neurons co-treated with Aβ42 and **(Mor-Cor)Ag(III)** manifesting reduction in the frequency of calcium events. Time stamps are shown in seconds. Scale bars: 100 μm.

**Video S4** Representative live-cell calcium imaging videos showing calcium transients in neurons treated with Aβ42 + **(Cor)Ag(III)** resulting in higher calcium activity compared to the Aβ42 + **(Mor-Cor)Ag(III).** Time stamps are shown in seconds. Scale bars: 100 μm.

**Table S1** Crystallographic data for **(Mor-Cor)Ag(III)**.

| Compound | **(Mor-Cor)Ag(III)** |
| --- | --- |
| Chemical formula | C_45_H_32_AgN_7_O_2_ |
| Formula mass | 810.65 |
| Crystal system | Triclinic |
| Space group | *P* -1 |
| Radiation | MoKα |
| a (Å) | 11.2981(3) |
| b (Å) | 12.7773(3) |
| c (Å) | 16.6302(5) |
| α (º) | 75.514(2) |
| β (º) | 88.335(2) |
| γ (º) | 72.988(2) |
| V (Å^3^) | 2220.17(11) |
| Z | 2 |
| T (K) | 295 |
| D_calcd_ (g cm^-3^) | 1.189 |
| Measured reflections | 49701 |
| e data (R_int_) | 9935 (0.048) |
| Parameters | 496 |
| Restraints | 0 |
| µ (mm^-1^) | 0.071 |
| 2*Ɵ* range (º) | 3.405-26.732 |
| R_1_ (I > 2σ (I)) | 0.0386 |
| wR_2_ (All data) | 0.1493 |
| GooF | 1.155 |
| Δρ_max_, Δρ_min_ (e Å^-3^) | 1.20, -0.34 |

*
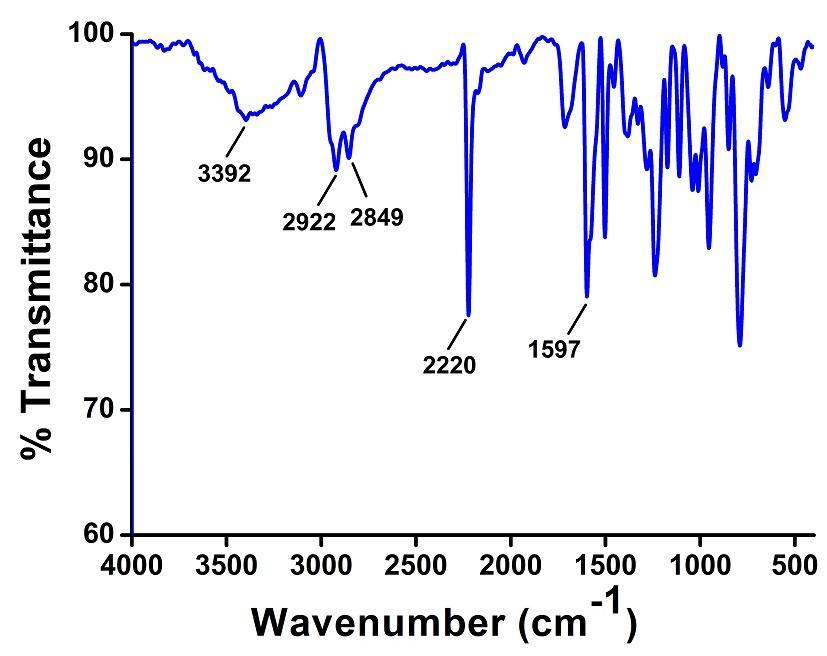
*

**Figure S1** FT IR spectra of **H_3_(Mor-Cor)**.


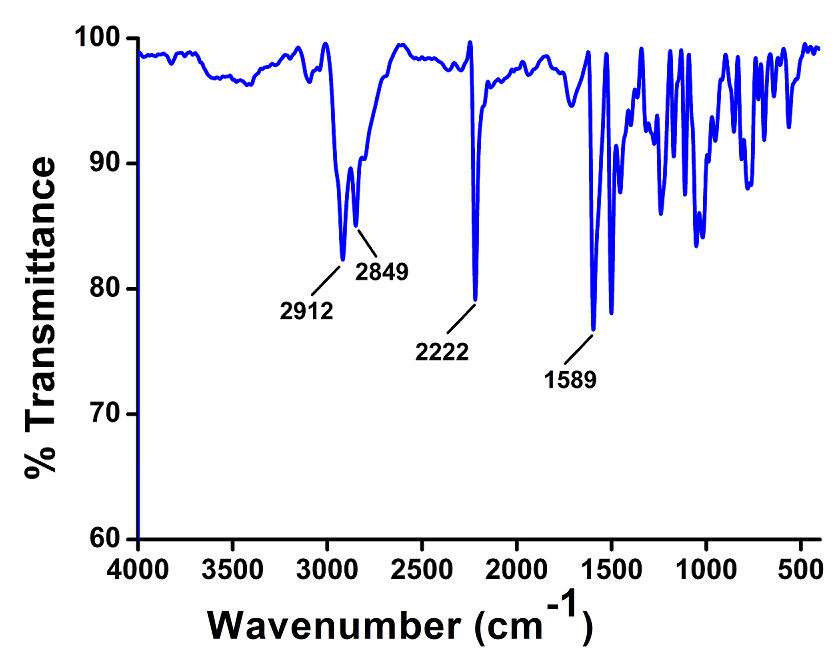


**Figure S2** FT IR spectra of **(Mor-Cor)Ag(III)**.


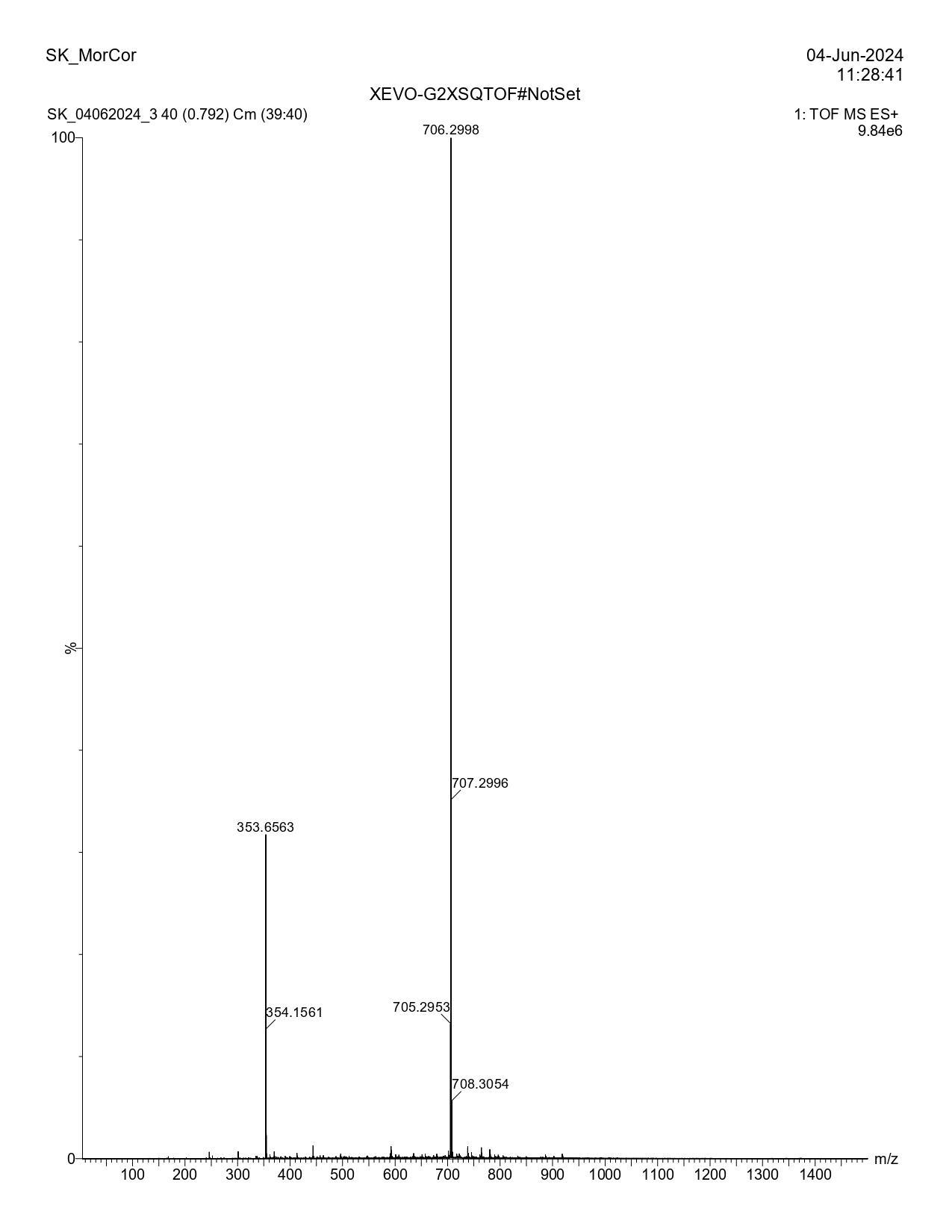


(a)


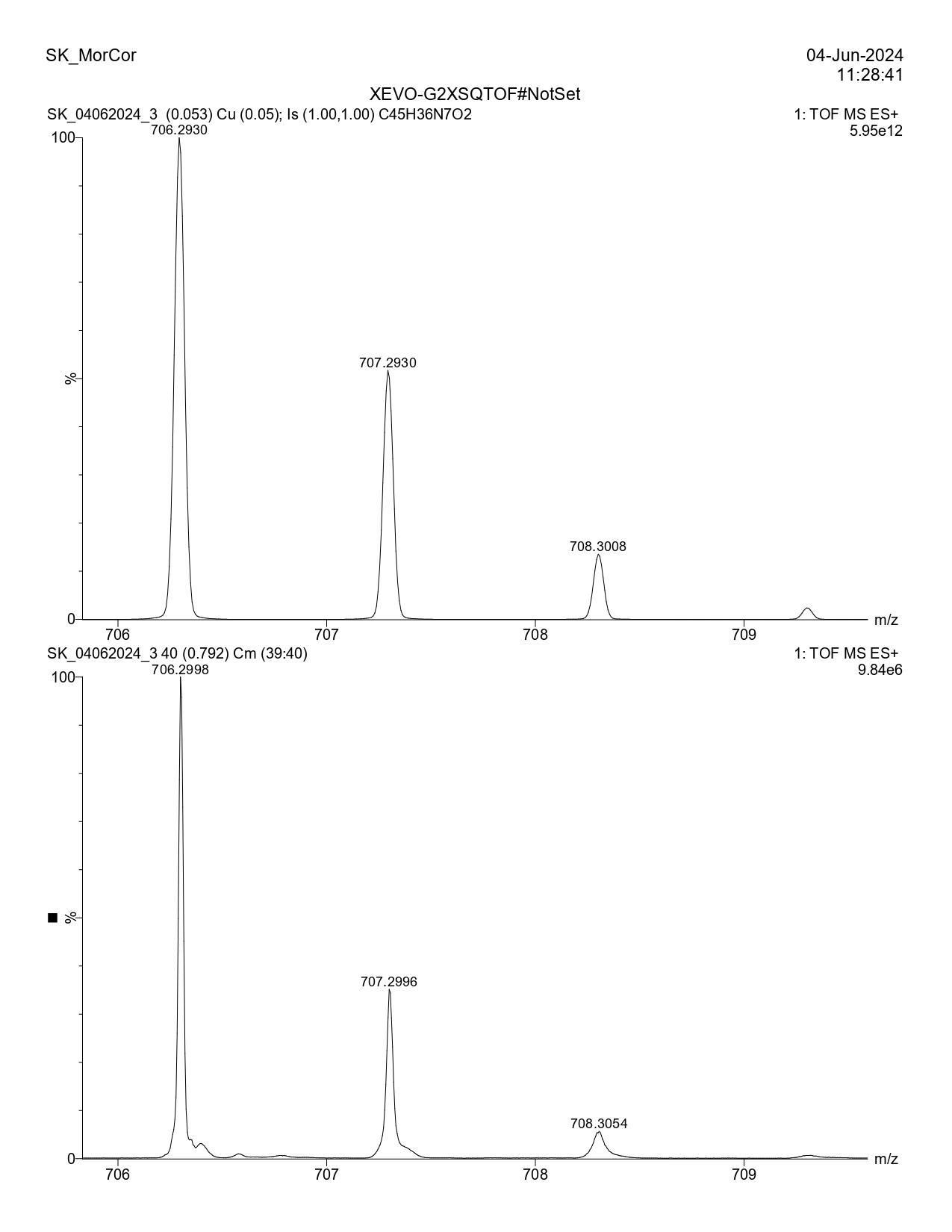


(b)

**Figure S3** ESI-MS spectrum of **H_3_(Mor-Cor)** in CH_3_CN shows the measured spectrum with (a) full region and (b) an isotopic distribution pattern.


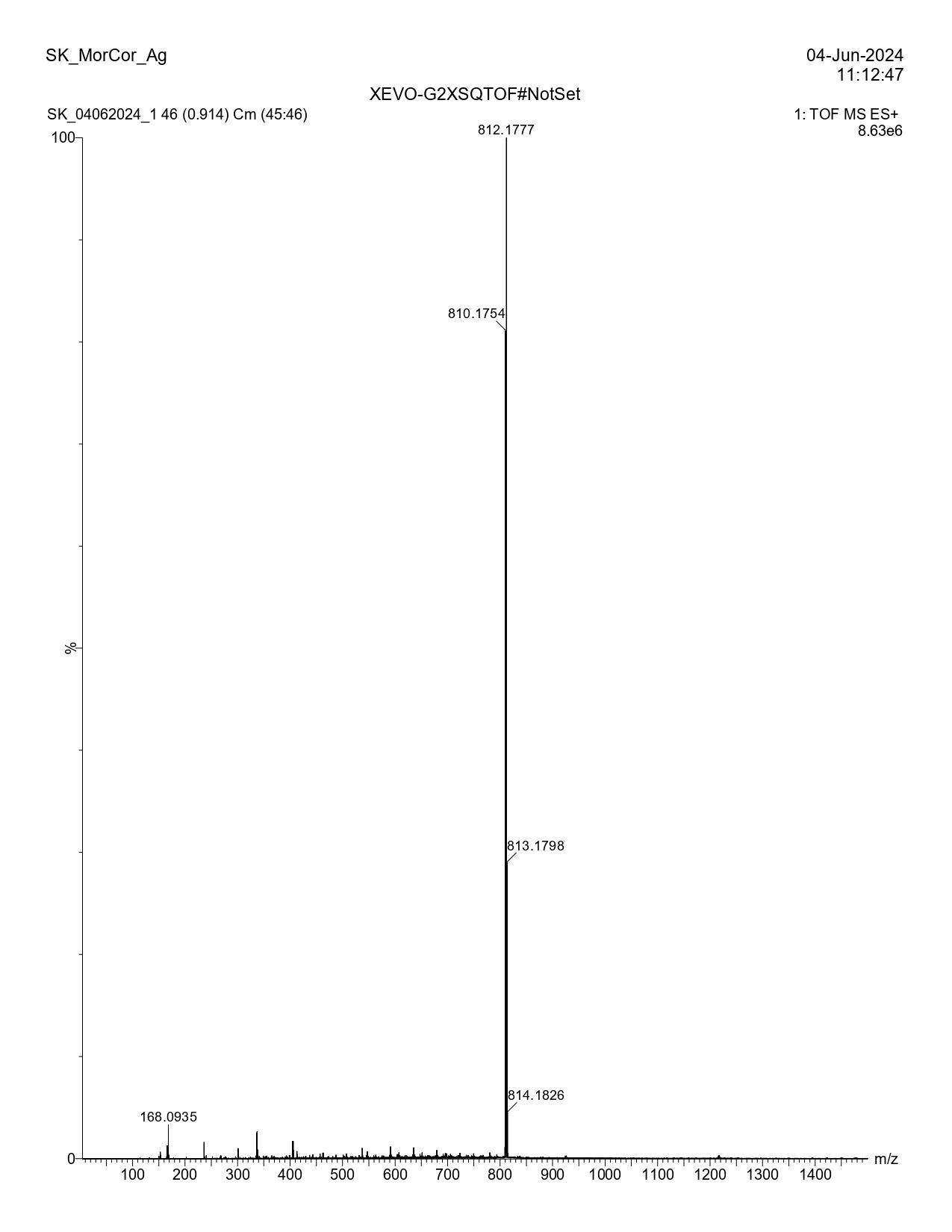


(a)


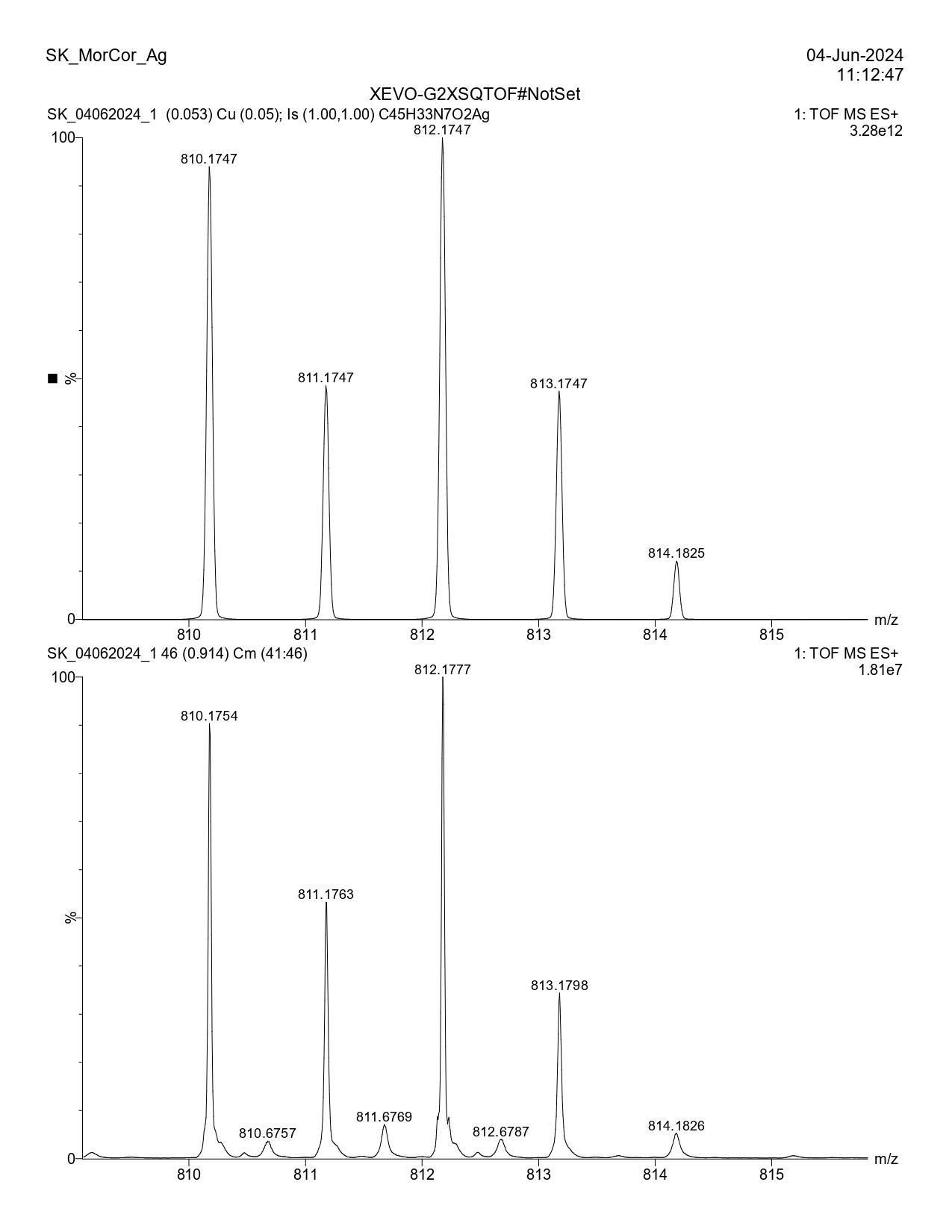


(b)

**Figure S4** ESI-MS spectrum of **(Mor-Cor)Ag(III)** in CH_3_CN shows the measured spectrum with (a) full region and (b) an isotopic distribution pattern.


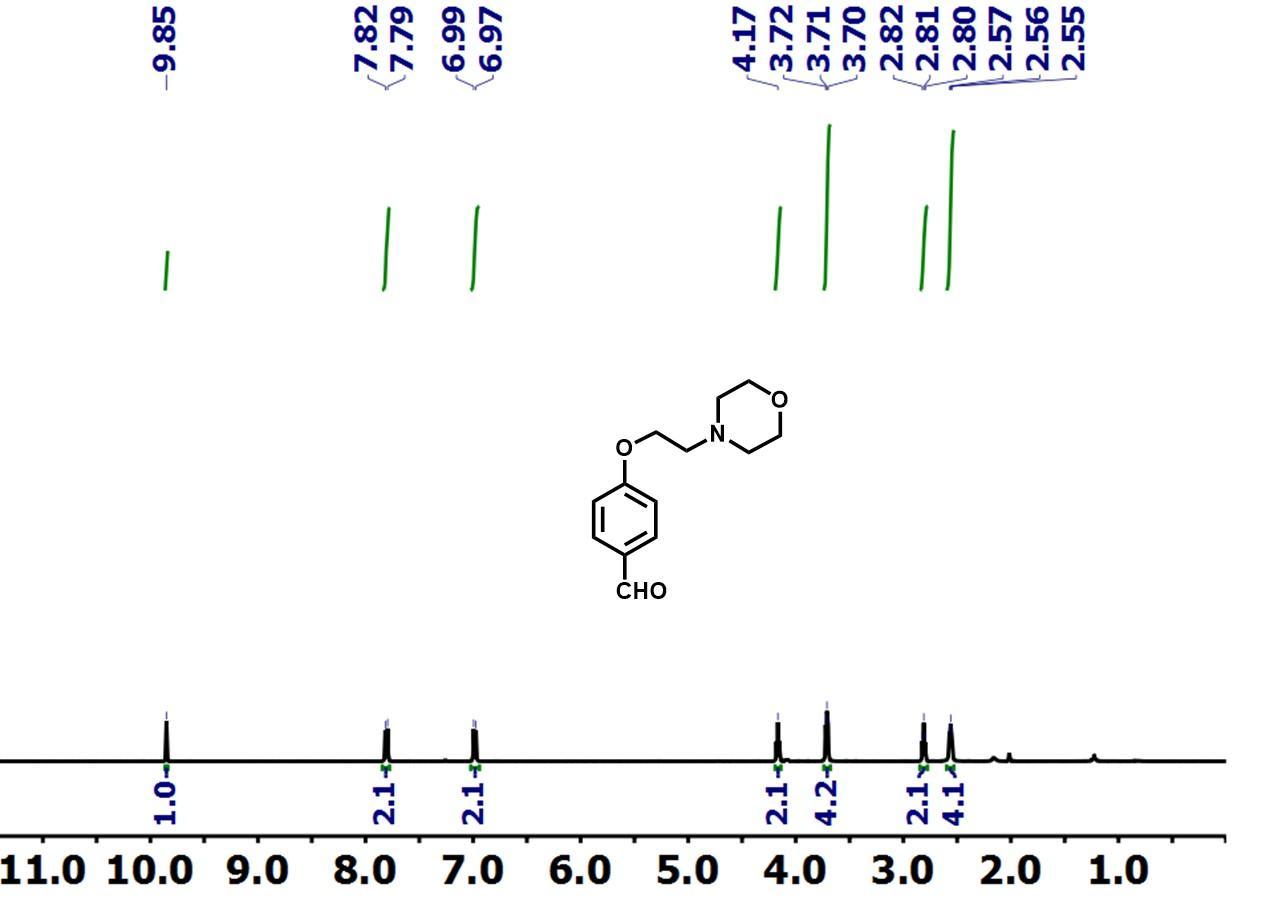


**Figure S5** ^1^H NMR (CDCl_3_, 400 MHz) spectrum of 4-(2-morpholinoethoxy)benzaldehyde.


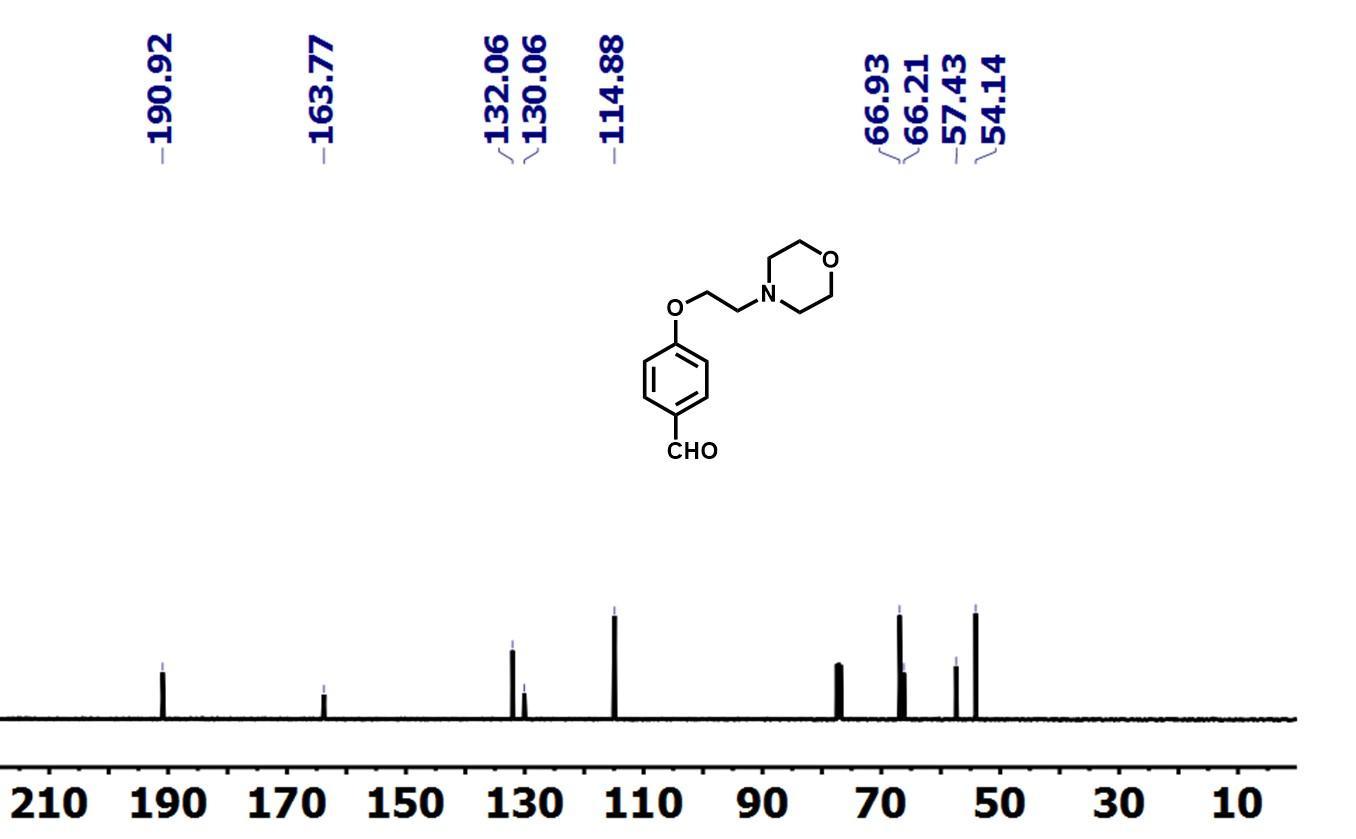


**Figure S6** ^13^C{^1^H} NMR (CDCl_3_, 101 MHz) spectrum of 4-(2-morpholinoethoxy)benzaldehyde.


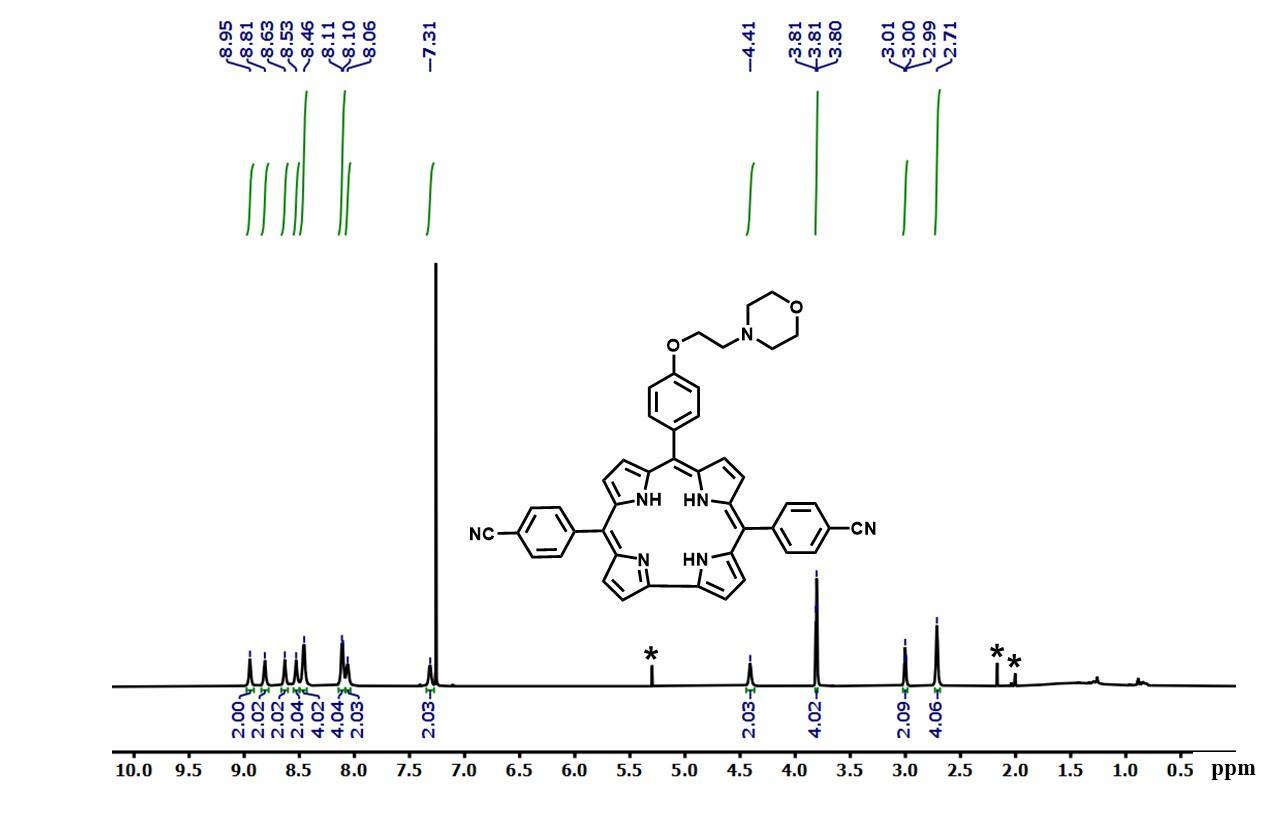


**Figure S7** ^1^H NMR (CDCl_3_, 400 MHz) spectrum of **H_3_(Mor-Cor)**.


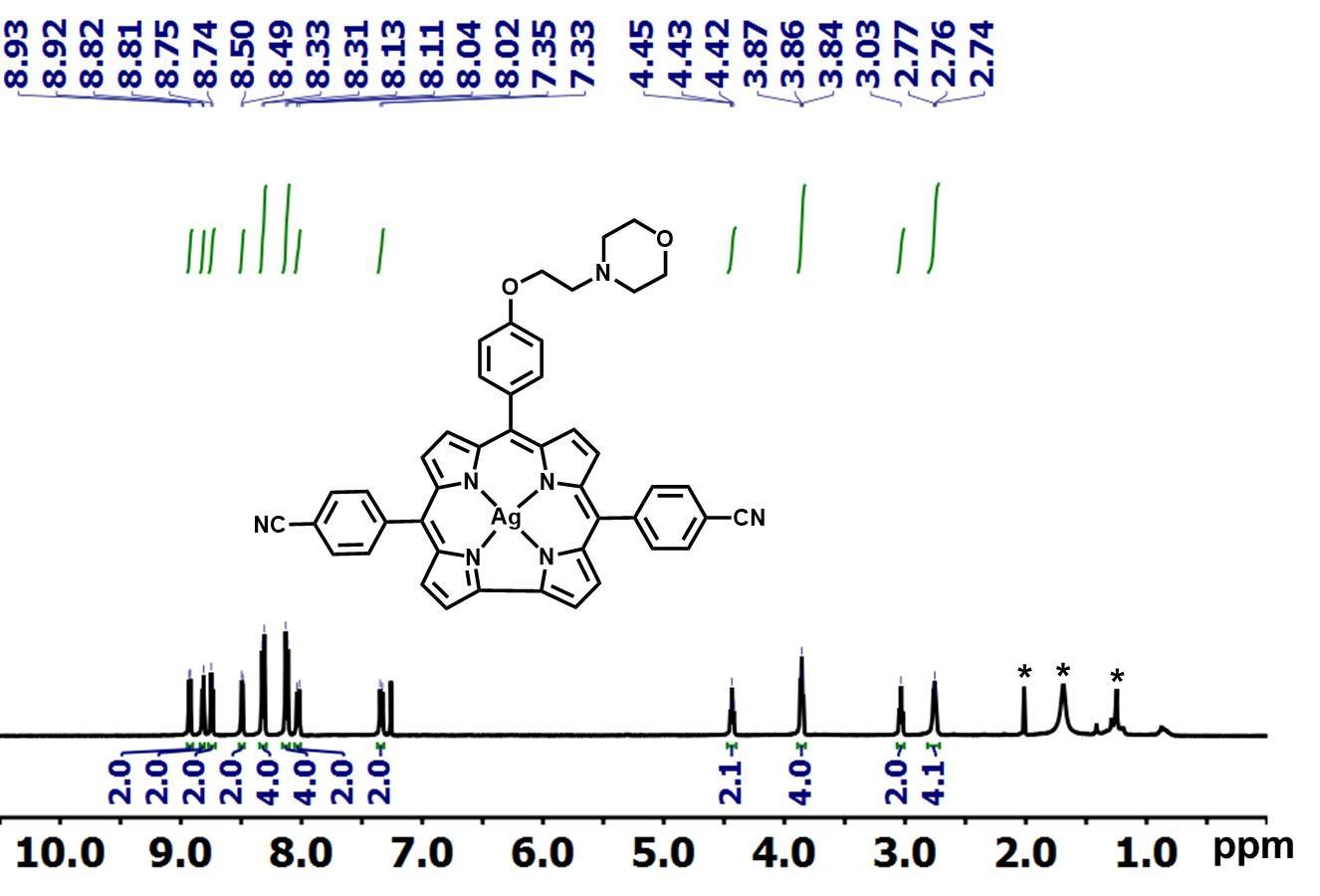


**Figure S8** ^1^H NMR (CDCl_3_, 400 MHz) spectrum of **(Mor-Cor)Ag(III)**.


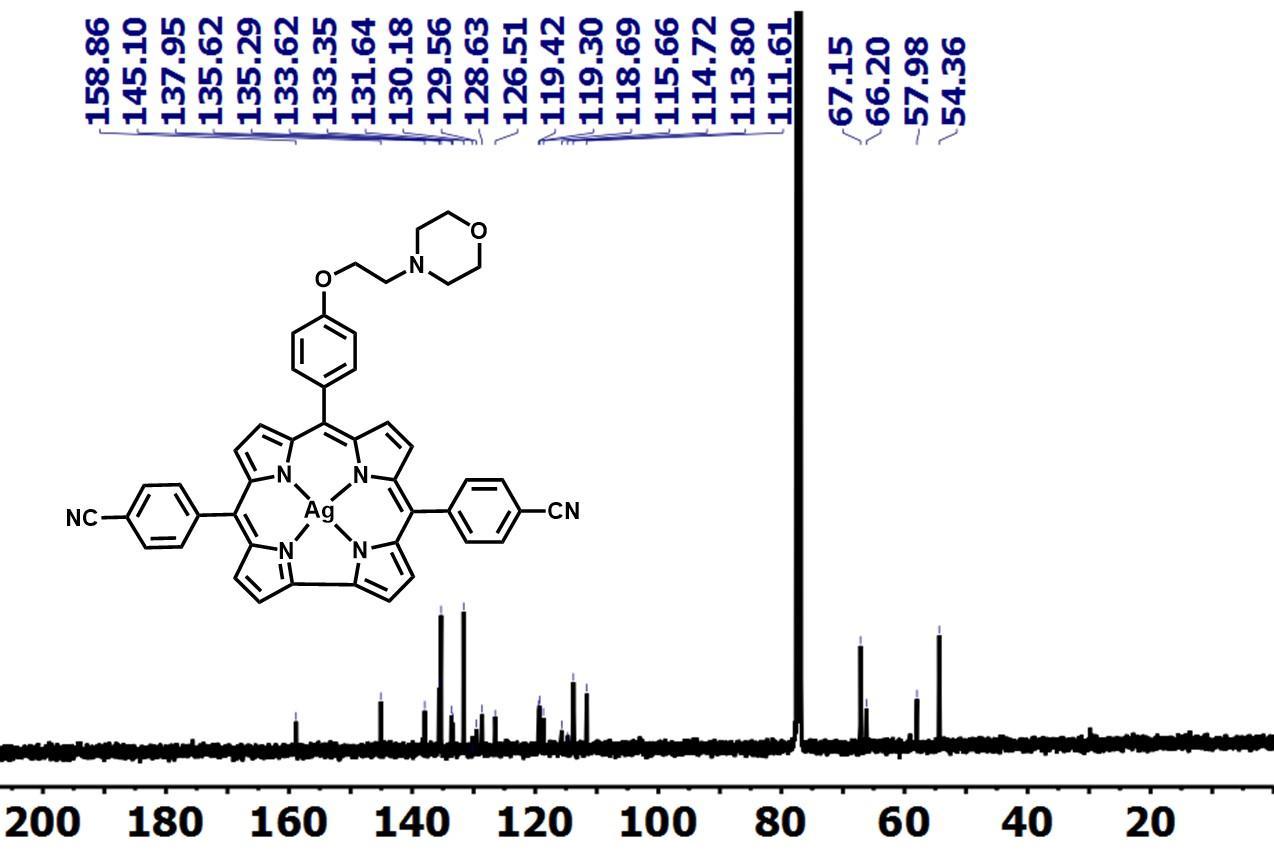


**Figure S9** ^13^C{^1^H} NMR (CDCl_3_, 101 MHz) spectrum of **(Mor-Cor)Ag(III)**.


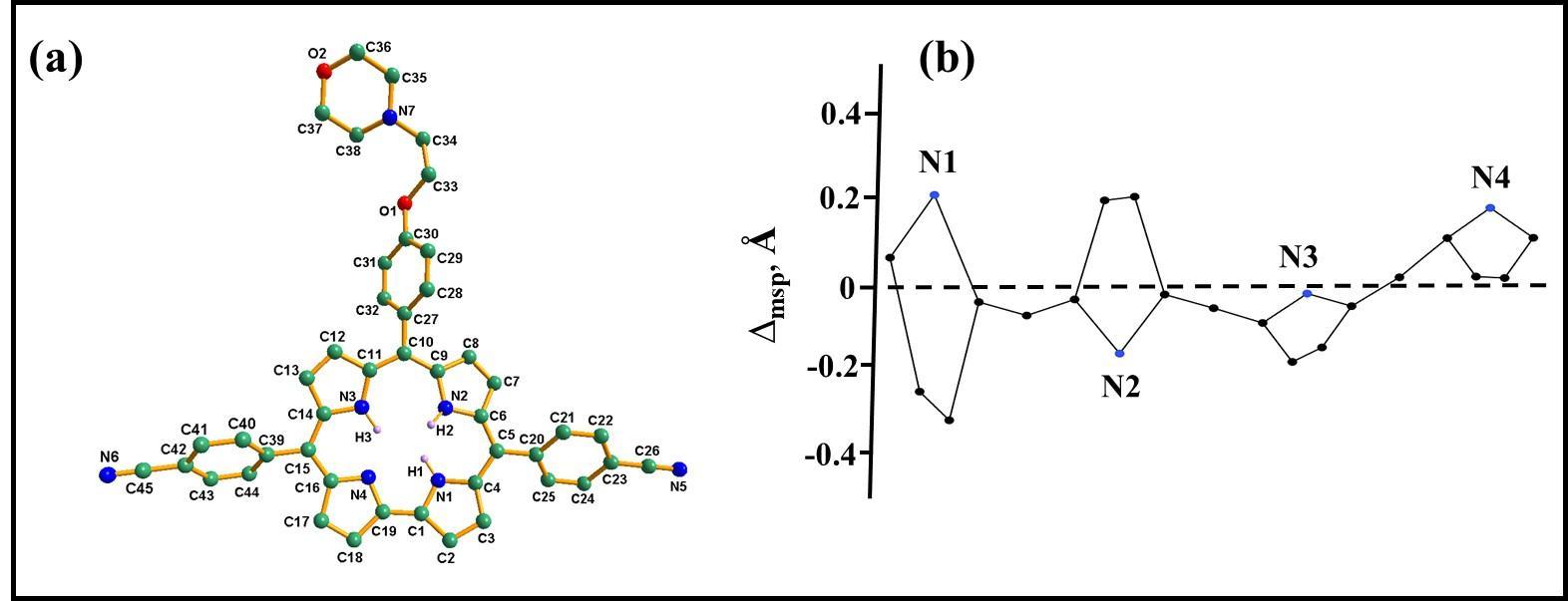


**Figure S10** (a) DFT-optimized geometry of **H_3_(Mor-Cor)** using the 6-311G (d, p) basis set and (b) linear display of non-planar distortions for **H_3_(Mor-Cor)**.


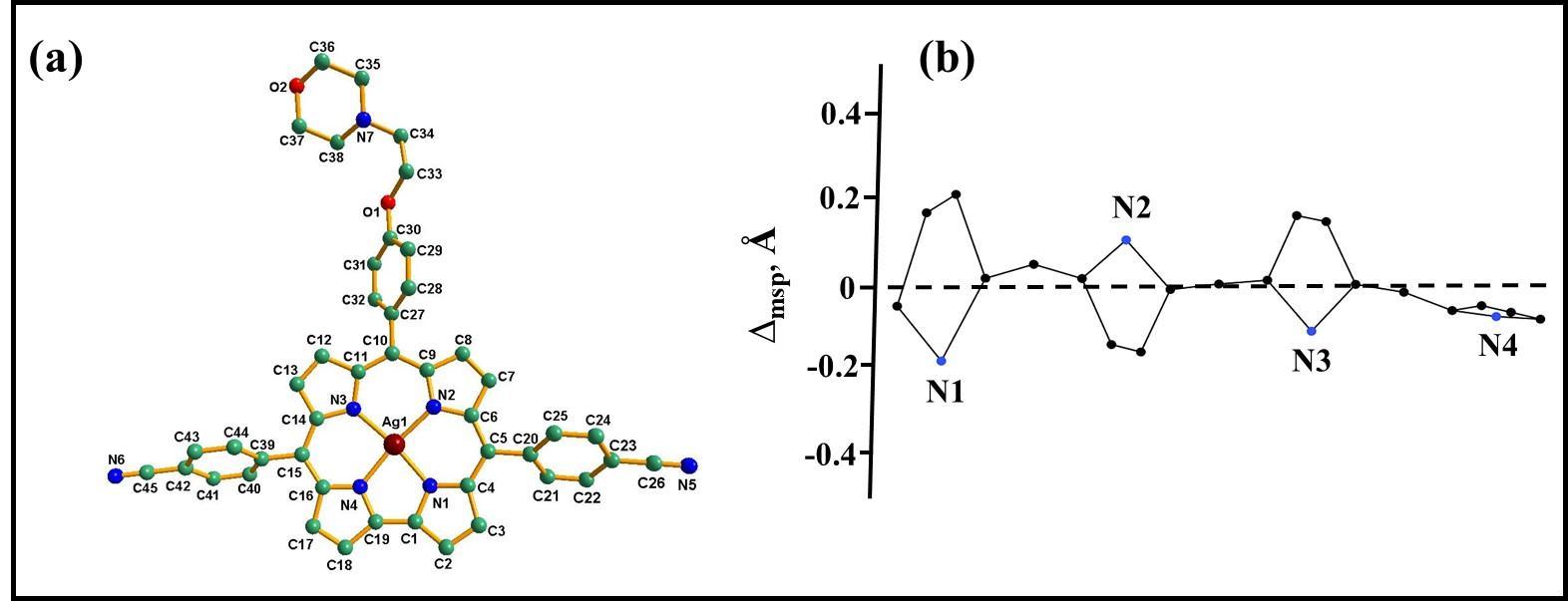


**Figure S11** (a) DFT-optimized geometry of **(Mor-Cor)Ag(III)** using the 6-311G (d, p) basis set and (b) linear display of non-planar distortions for **(Mor-Cor)Ag(III)**.

**(a) (b)**

**Figure S12** Selected bond distances of **(Mor-Cor)Ag(III)** from (a) Crystal structure and (b) DFT optimized structure.


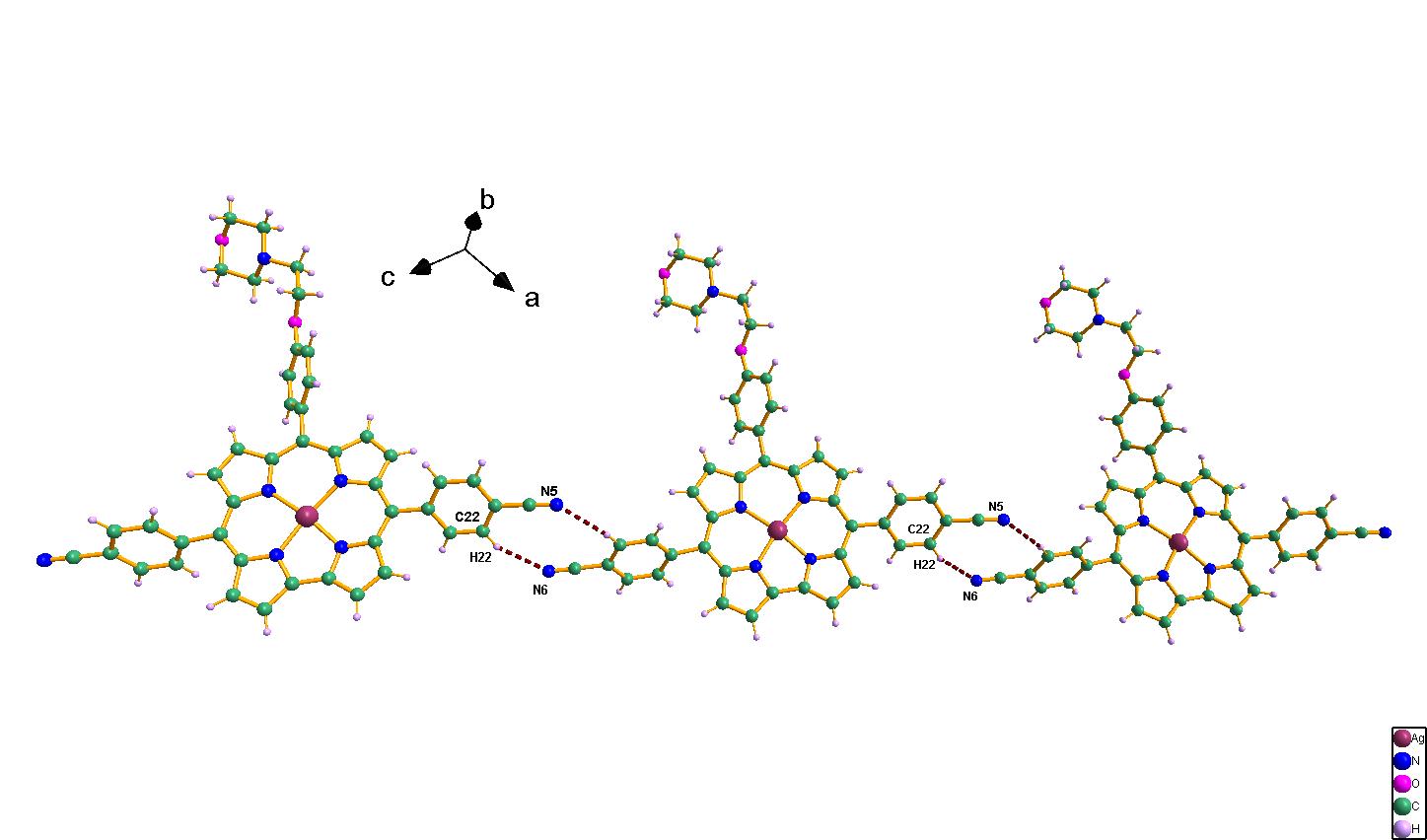


**Figure S13** Chain-like supramolecular assembly in **(Mor-Cor)Ag(III)**.


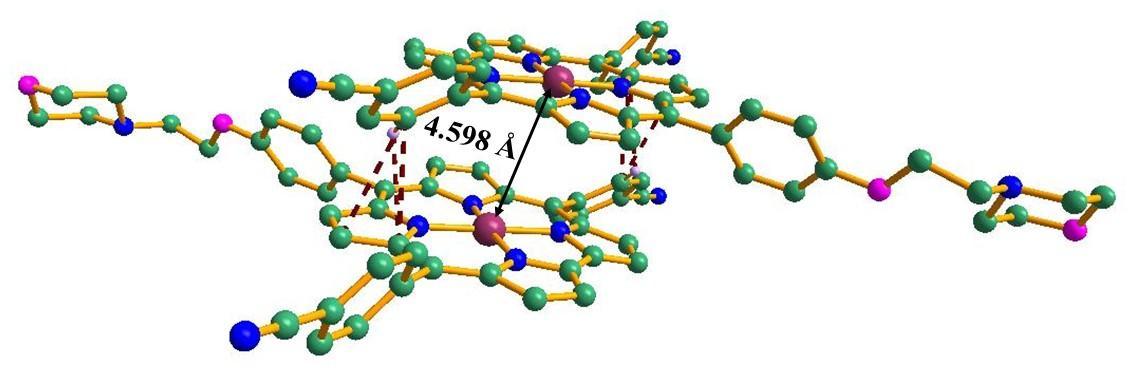


**Figure S14** C-H^…^π interactions in **(Mor-Cor)Ag(III)**.


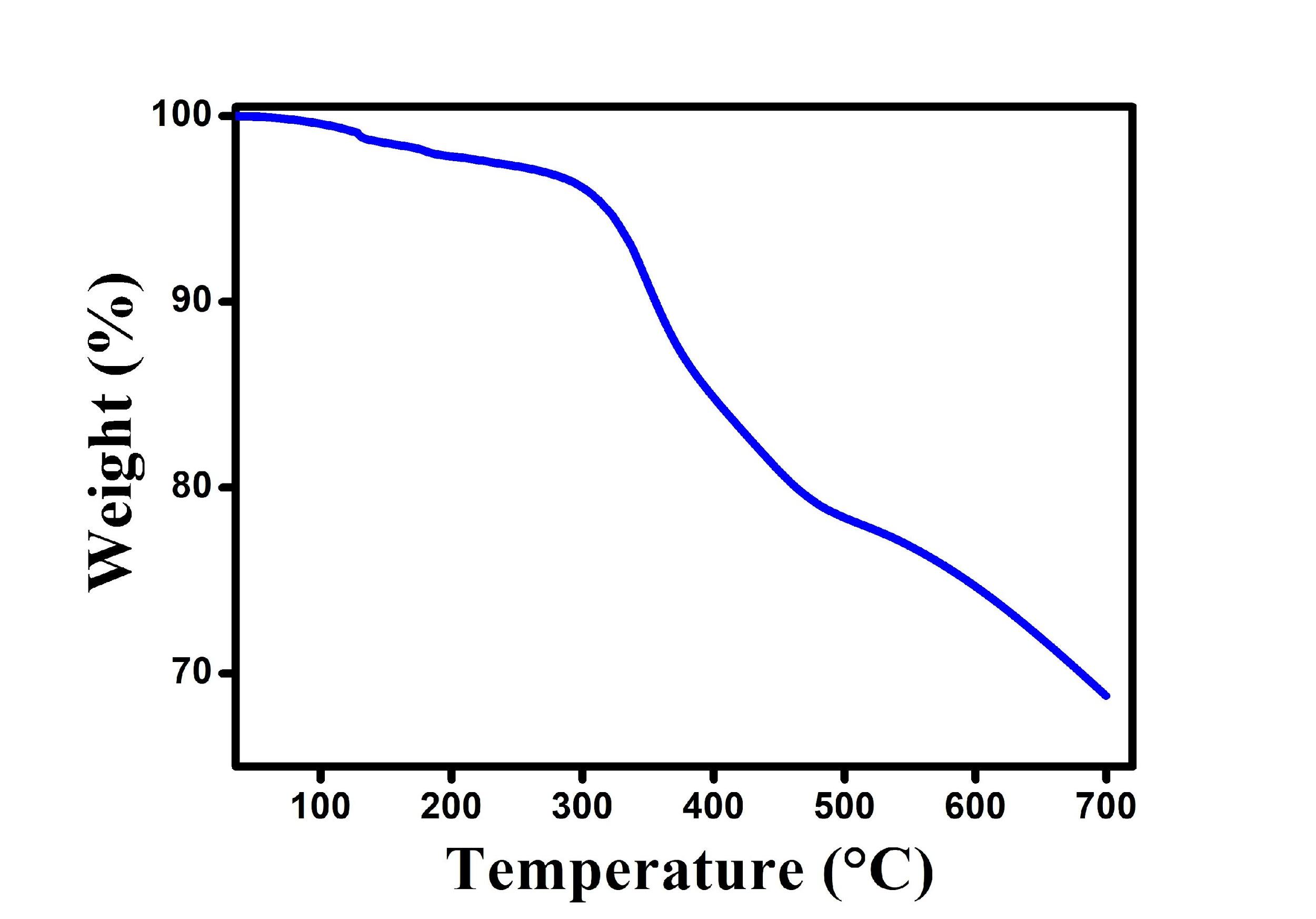


**Figure S15** TGA thermogram of **(Mor-Cor)Ag(III)**.

**Table S2** Some important hydrogen bond parameters of **(Mor-Cor)Ag(III)**.

| Compound | D-H···A | ^d^D-H (Å) | ^d^H···A (Å) | ^d^D···A (Å) | ∠D-H···A (^o^) |
| --- | --- | --- | --- | --- | --- |
| **(Mor-Cor)Ag(III)** | C(41)-H(41)…N(5)  C(24)-H(24)…N(6)  C(33)-H(33B)…N(3)  C(33)-H(33B)…C(11) | 0.93  0.93  0.97  0.97 | 2.549  2.638  2.706  2.810 | 3.436  3.519  3.512  3.716 | 159.46  158.31  140.87  149.36 |

**Table S3** Most important photophysical data of compounds; **H_3_(Mor-Cor)** and **(Mor-Cor)Ag(III)** in CH_2_Cl_2_ solution at 298 K.

| Compounds | UV-Vis data ^a^ | | Emission data^a, b^ | | | Electrochemical data ^a, c^ | |
| --- | --- | --- | --- | --- | --- | --- | --- |
|  | λ_abs_ / nm | ε /10^5^ M^-1^ cm^-1^ | λ_ex_ (nm) | λ_em_  (nm) | Φ_em_^a^ | Oxidation  E^0^, V  (∆E_p_, mV) | Reduction  E^0^, V  (∆E_p_, mV) |
| **H_3_(Mor-Cor)** | 424, 580, 629, 656 | 1.04, 0.17,  0.12, 0.11 | 424 | 683 | 0.12 | - | - |
| **(Mor-Cor)Ag(III)** | 427, 588 | 1.02, 0.28 | 427 | - | - | +0.77 | -0.83 |
| ^a^In CH_2_Cl_2_^. b^Emission quantum yields were calculated using coumarin (*Φ*_em_= 0.54) as the reference. ^c^The potentials are vs. Ag/AgCl | | | | | | | |

**
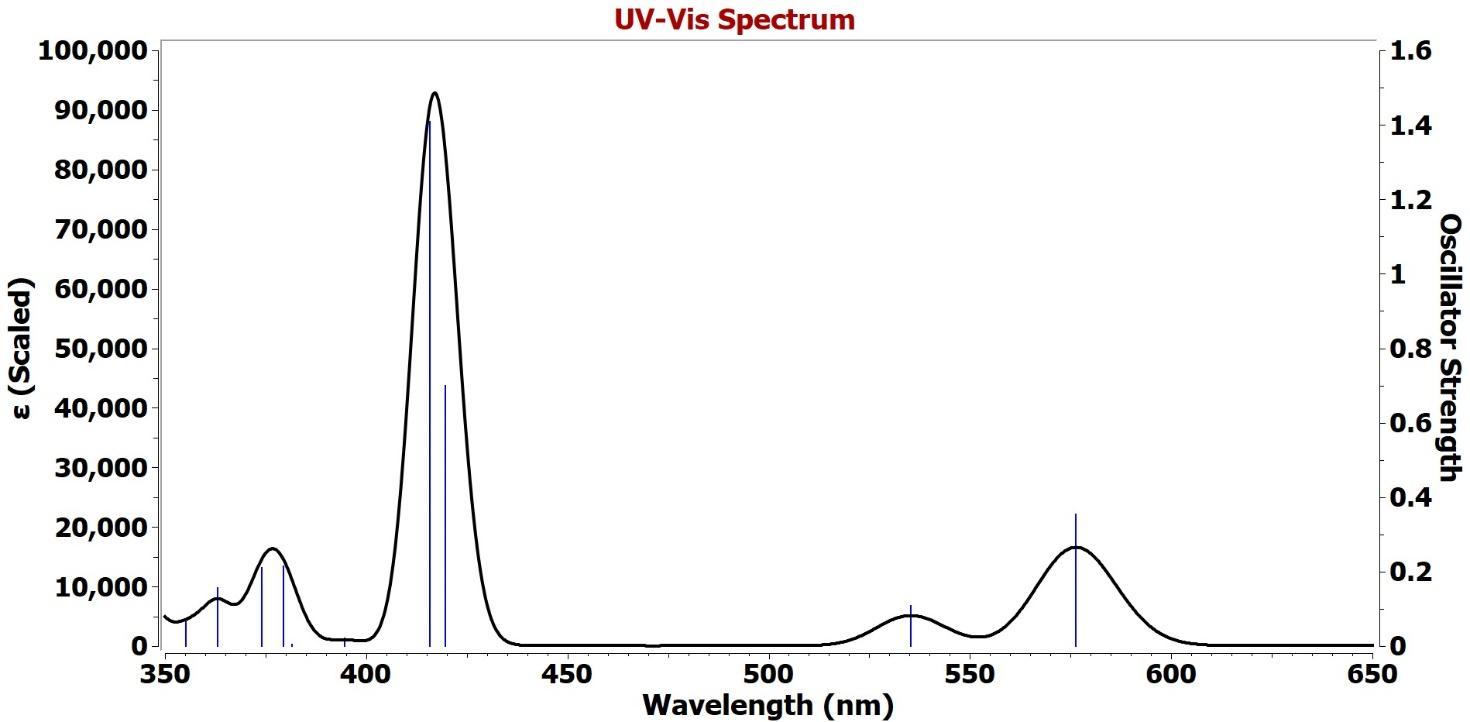
**

**Figure S16** TD-DFT-based electronic absorption spectra of **H_3_(Mor-Cor)**.

**Table S4** TD-DFT Calculated Electronic Transitions for **H_3_(Mor-Cor)**.

| **State** | **Energy**  **(cm^-1^)** | **Wavelength**  **(nm)** | **Oscillator Strength** | **Orbital contributions^a^** |
| --- | --- | --- | --- | --- |
| S1 | 17350.71 | 576.34 | 0.3544 | HOMOLUMO (65%)  HOMO-1LUMO+1 (18%) |
| S2 | 18679.12 | 535.35 | 0.1086 | HOMO-1LUMO (41%)  HOMOLUMO+1 (42%)  HOMOLUMO (12%) |
| S3 | 23831.42 | 419.61 | 0.6986 | HOMOLUMO+1 (32%)  HOMOLUMO+2 (16%) HOMOLUMO+3 (14%)  HOMO-1LUMO (26%) |
| S4 | 24046.77 | 415.85 | 1.4089 | HOMO-1LUMO+1 (47%)  HOMOLUMO+2 (17%)  HOMOLUMO+3 (19%) |
| S5 | 25336.46 | 394.68 | 0.0207 | HOMO-3LUMO (12%)  HOMO-2LUMO (85%) |
| S6 | 26210.78 | 381.52 | 0.0047 | HOMO-3LUMO (76%)  HOMO-2LUMO (14%) |


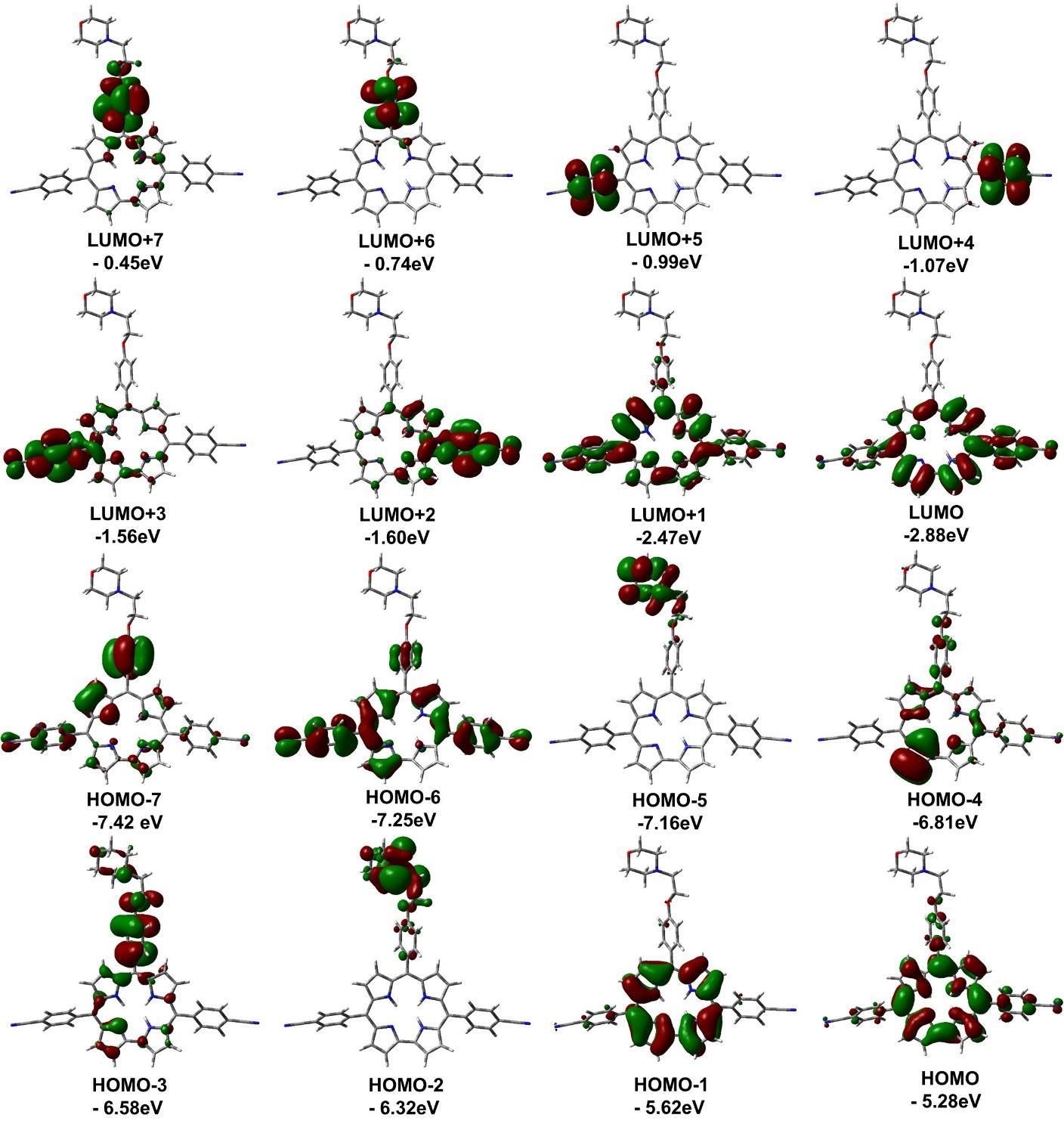


**Figure S17** Selected frontier MOs with orbital energies of **H_3_(Mor-Cor)**.

**
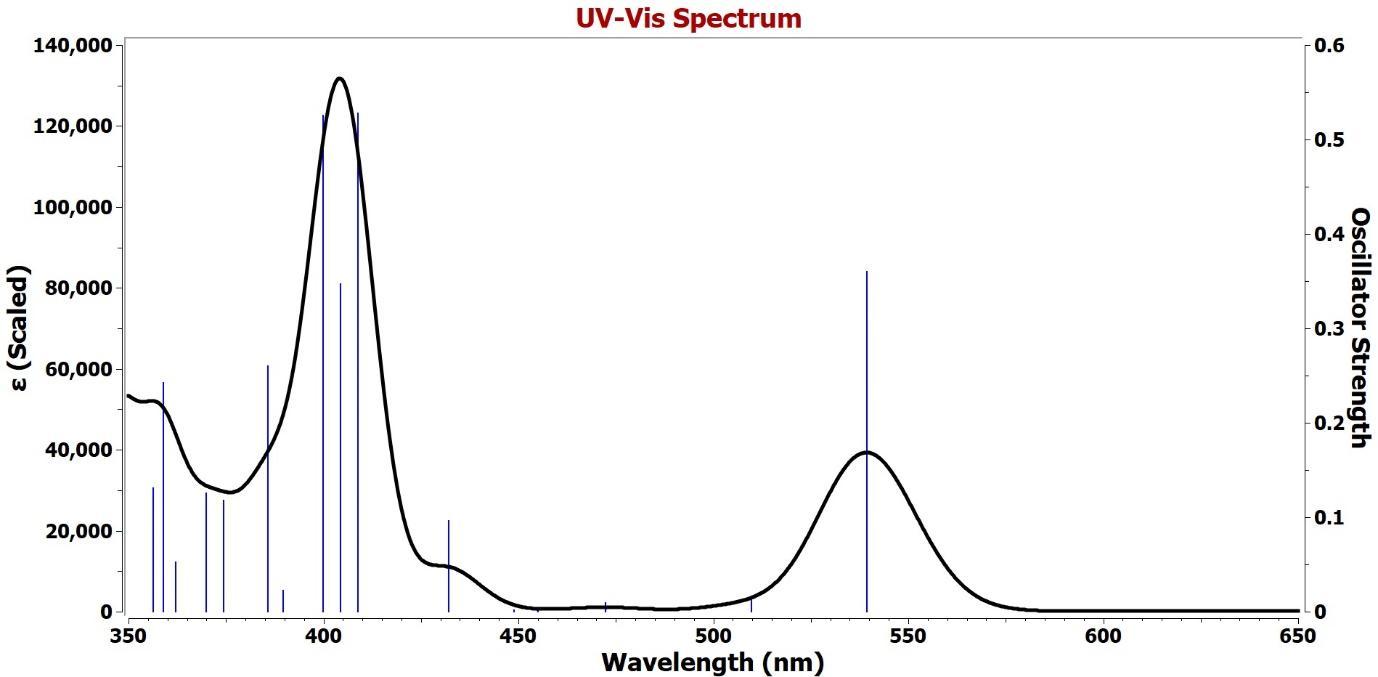
**

**Figure S18** TD-DFT-based electronic absorption spectra of **(Mor-Cor)Ag(III)**.

**Table S5** TD-DFT Calculated Electronic Transitions for **(Mor-Cor)Ag(III)**.

| **State** | **Energy**  **(cm^-1^)** | **Wavelength**  **(nm)** | **Oscillator Strength** | **Orbital contributions^a^** |
| --- | --- | --- | --- | --- |
| S1 | 18540.39 | 539.36 | 0.3598 | HOMO-1LUMO+2 (15%)  HOMOLUMO+1 (80%) |
| S2 | 19616.34 | 509.77 | 0.0163 | HOMO-1LUMO+1 (57%)  HOMOLUMO+2 (39%) |
| S3 | 21170.58 | 472.35 | 0.009 | HOMO-3LUMO (37%)  HOMO-2LUMO (60%) |
| S4 | 21978.76 | 454.98 | 0.0005 | HOMO-3LUMO (51%)  HOMO-1LUMO+2 (40%) |
| S5 | 22279.60 | 448.84 | 0.0017 | HOMO-4LUMO (92%) |
| S6 | 23143.43 | 432.08 | 0.0963 | HOMO-5LUMO (77%) |
| S7 | 24462.96 | 408.78 | 0.5257 | HOMO-1LUMO+1 (15%)  HOMOLUMO+2 (26%)  HOMOLUMO+3 (20%) |
| S8 | 24727.51 | 404.40 | 0.3469 | HOMO-7LUMO (43%)  HOMO-1LUMO+2 (14%)  HOMOLUMO+4 (11%) |
| S9 | 25008.19 | 399.86 | 0.5257 | HOMO-7LUMO (28%)  HOMO-1LUMO+2 (26%)  HOMOLUMO+3 (18%)  HOMOLUMO+4 (13%) |
| S10 | 25667.96 | 389.59 | 0.0223 | HOMO-2LUMO+1 (72%)  HOMOLUMO+3 (14%) |

**
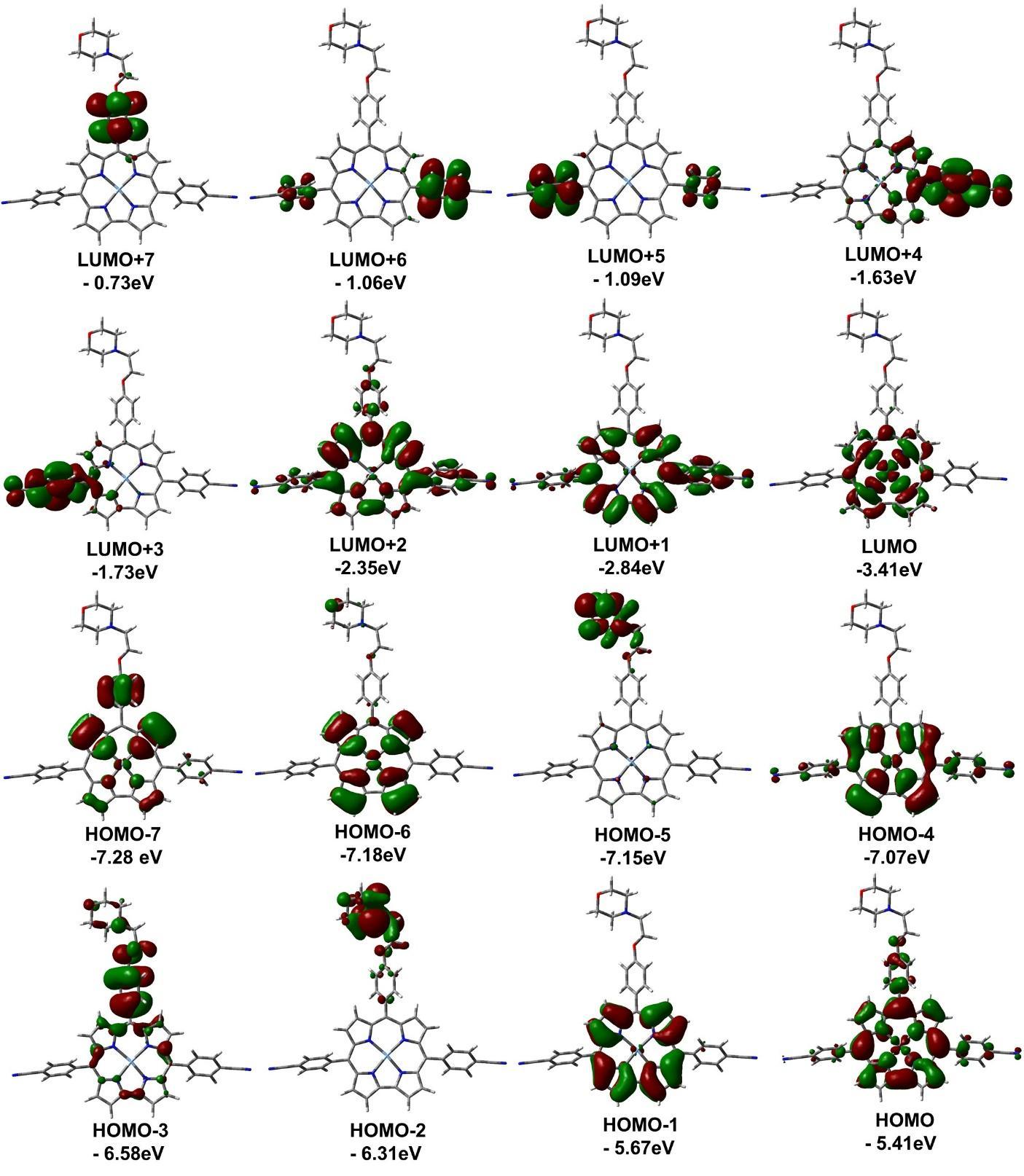
**

**Figure S19** Selected frontier MOs with orbital energies of **(Mor-Cor)Ag(III)**.


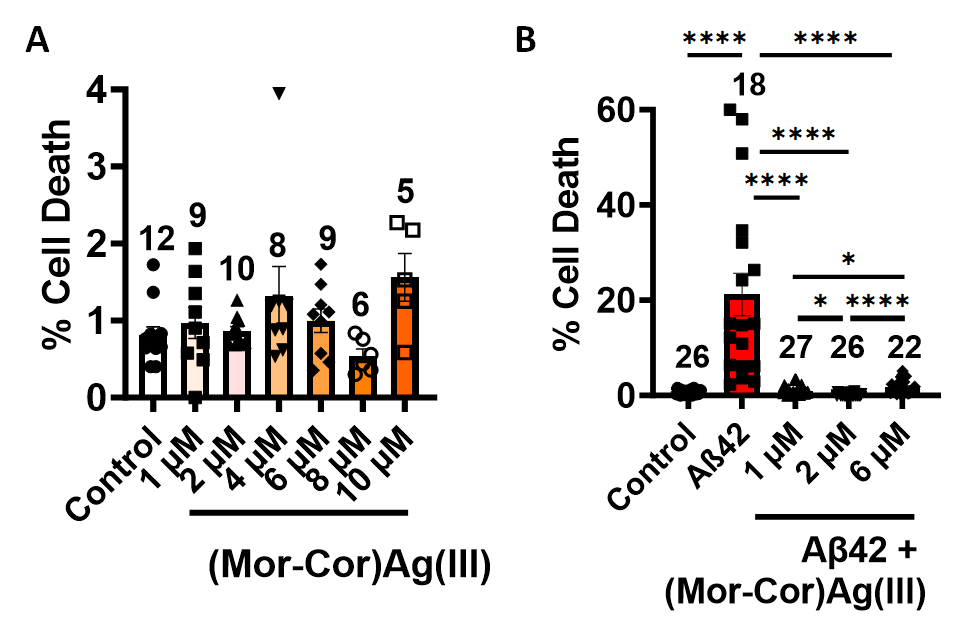


**Figure S20** Aβ42 exposed HT-22 hippocampal cells manifest decreased cell death on treatment with **(Mor-Cor)Ag(III)** at various concentrations (A) Quantification of cell death to determine toxicity of the compound. (B) Quantification of cell death after applying Aβ42 with different concentrations of compounds. Data are mean ± SEM. Statistical significance analysed by Kruskal Walis test with posthoc test, Dunn’s multiple comparison test. Sample size listed above bar graphs (*p < 0.05; **p < 0.01; ***p < 0.001;  **** p < 0.0001) acquired from 3 independent experiments.


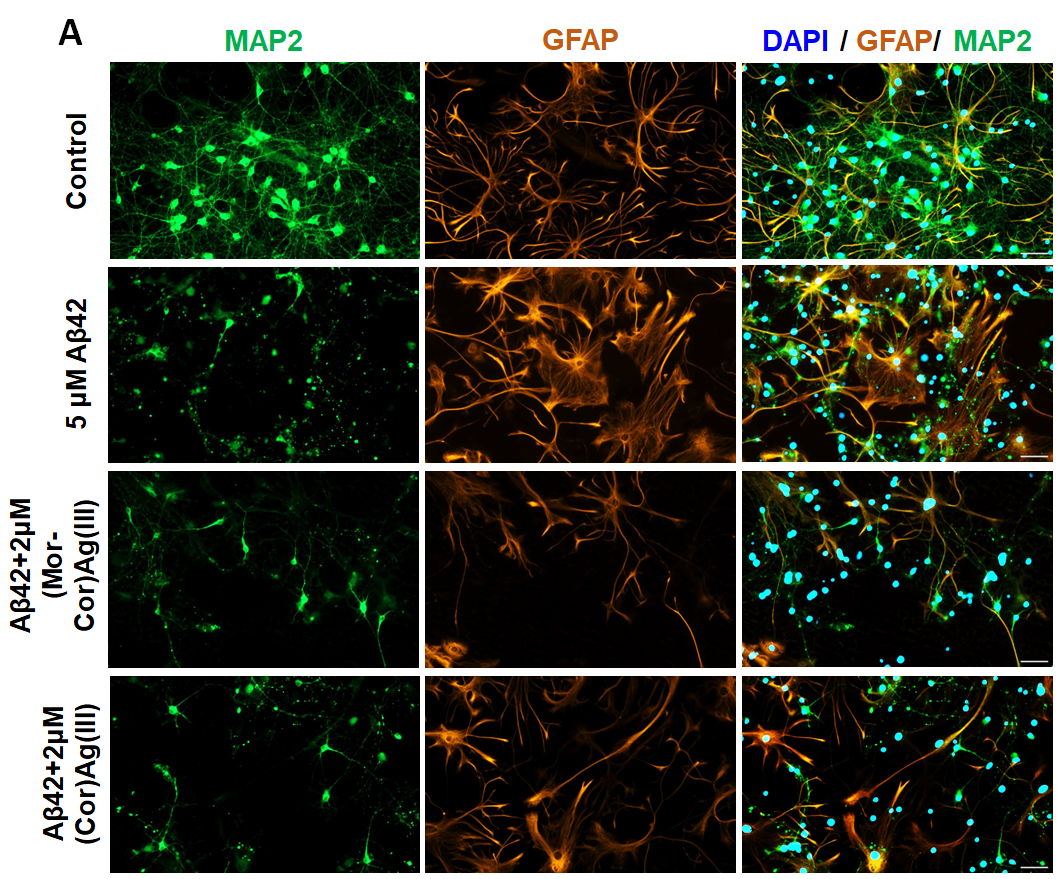


**Figure S21** Aβ42- exposed neurons and astrocytes show decreased pathological characteristics on treatment with **(Mor-Cor)Ag(III)**. Representative images of neurons expressing MAP2 (green) and astrocytes expressing GFAP (orange) in 20X magnification.


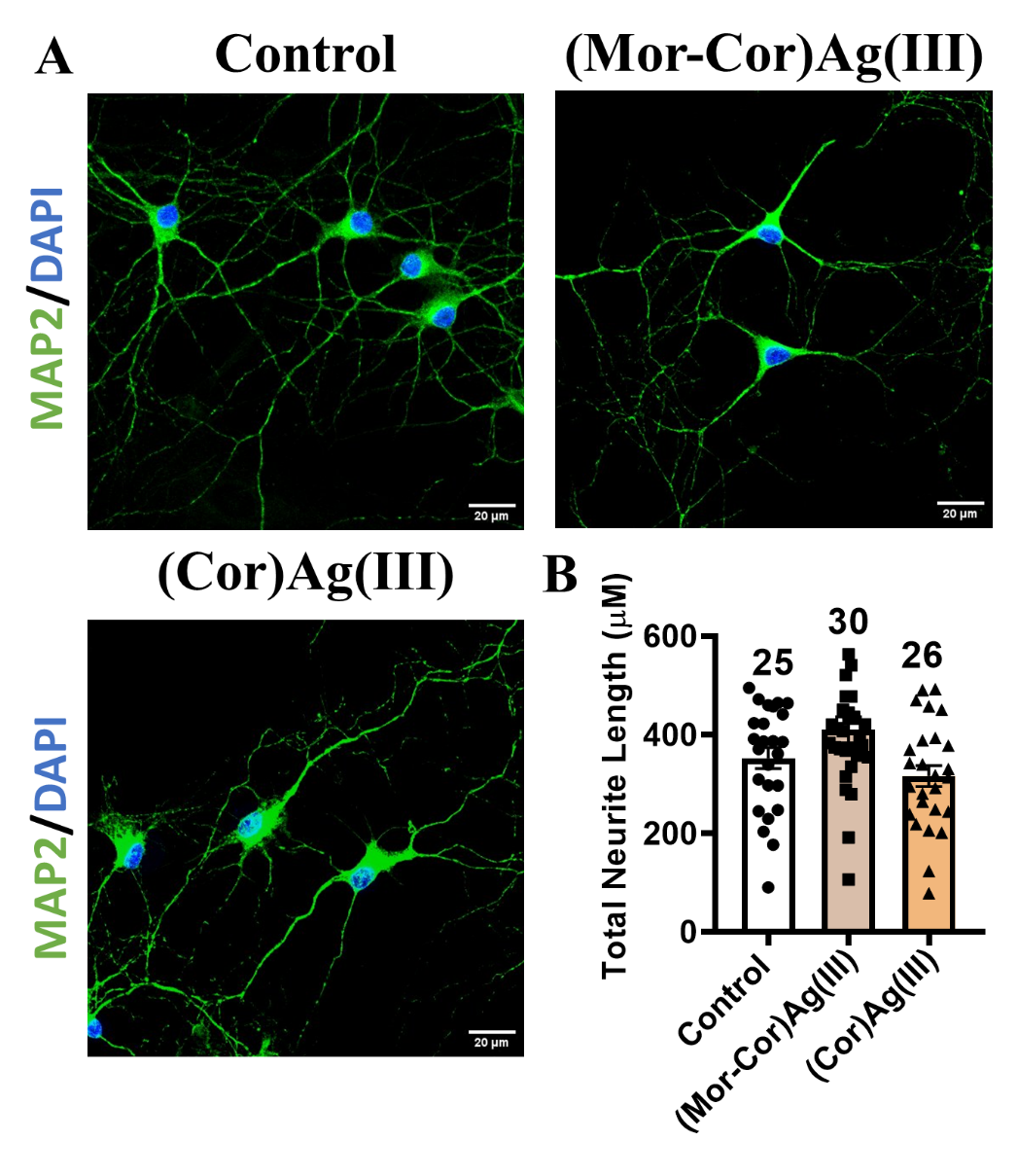


**Figure S22** No significant effect of compound-only treatment on total neurite length (A) Representative images showing no change in total neurite length in the compound-only control group. (B) Quantification of total neurite length, confirming the absence of changes in the compound-only control condition. Data are presented as mean ± SEM. Statistical significance was analyzed using ANOVA followed by Sidak’s multiple comparisons test between selected pairs (P > 0.05).


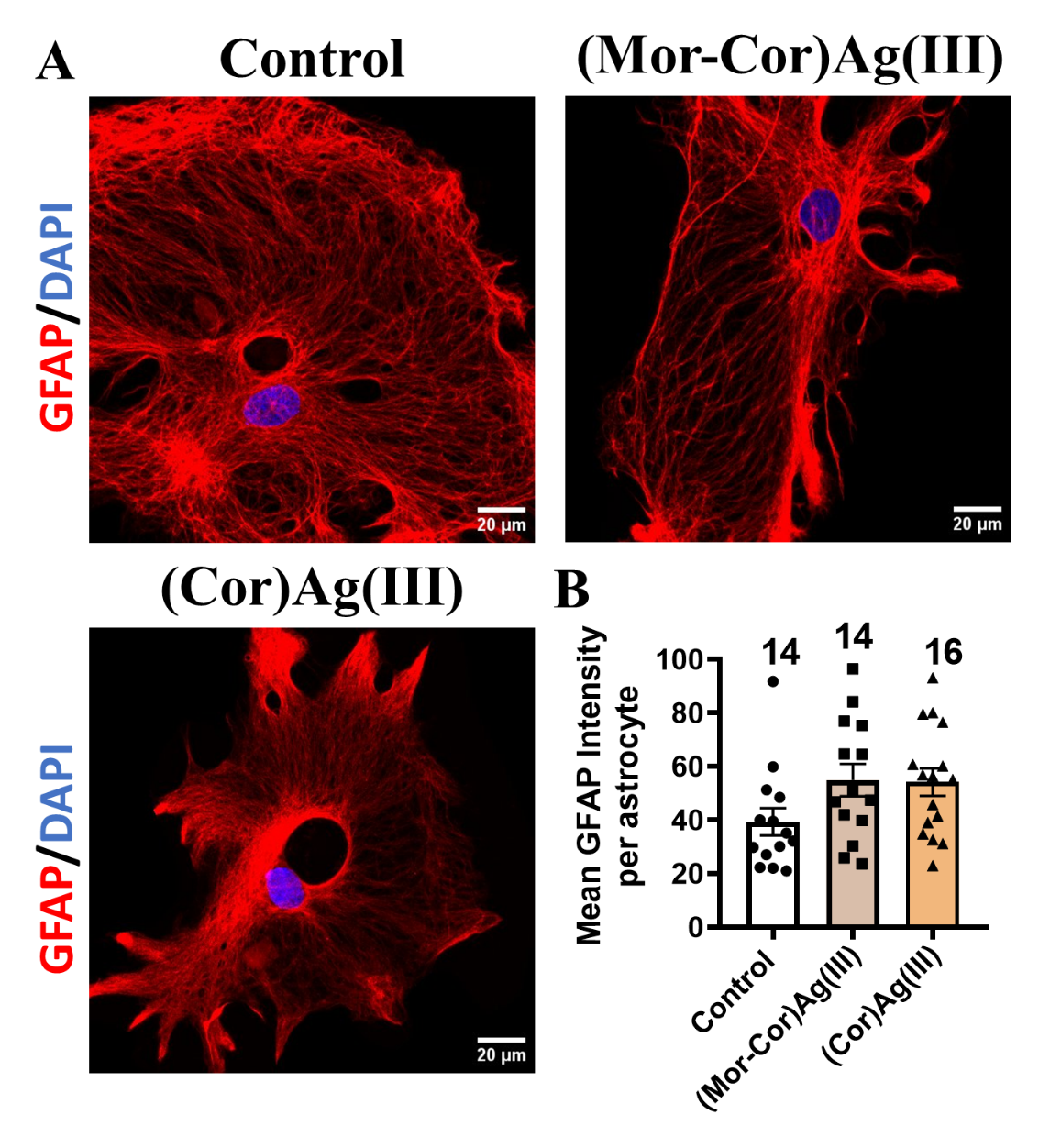


**Figure S23** No significant change in GFAP intensity following compound-only treatment. (A) Representative images showing no change in GFAP intensity in the compound-only control group. (B) Quantification of GFAP intensity confirming the absence of changes in the compound-only control condition. Data are mean ± SEM. Statistical significance analyzed by ANOVA with Sidak’s multiple comparison test between selected pairs (P > 0.05).


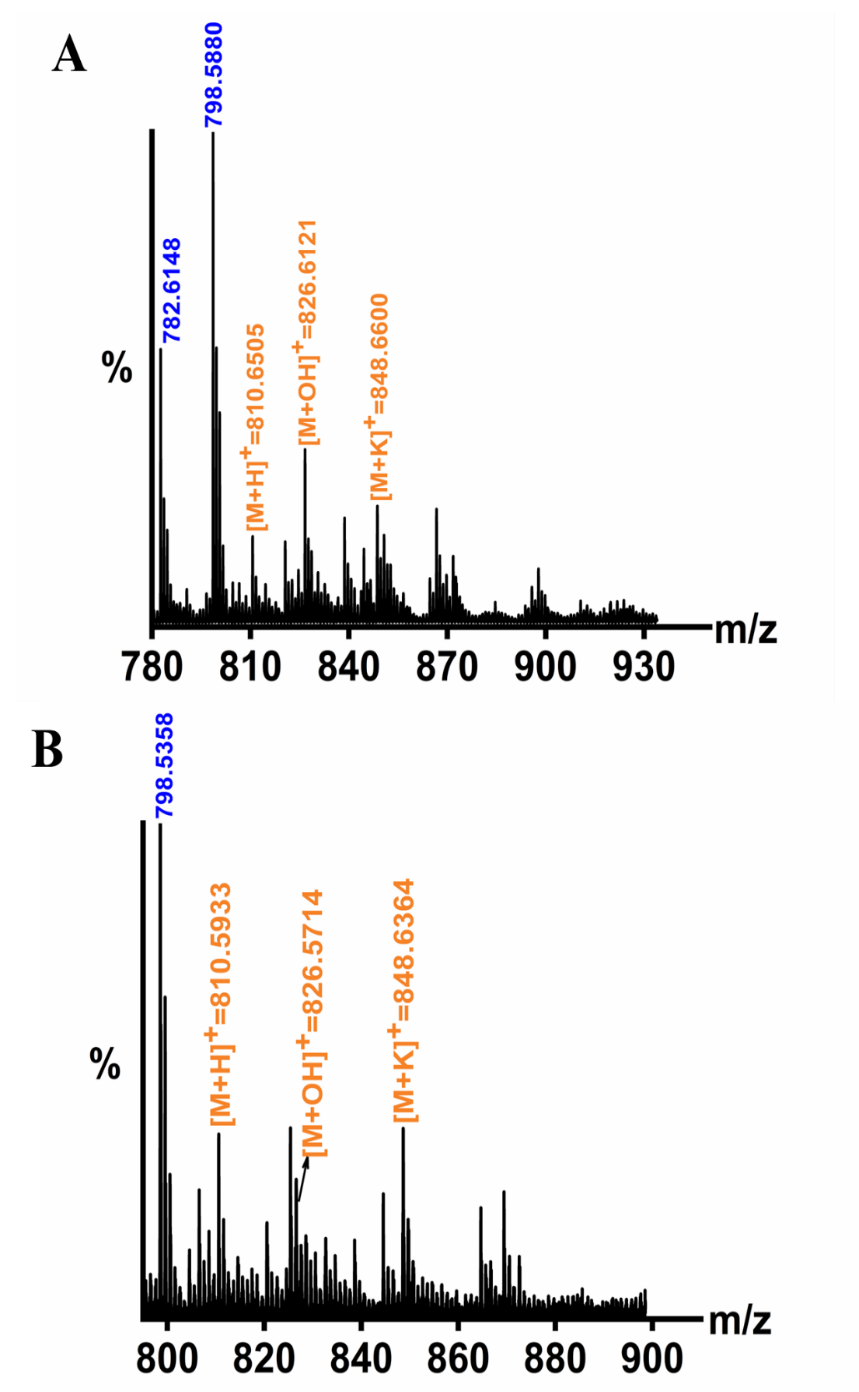


**Figure S24** Biological replicates of ESI-MS spectra confirming brain accumulation of **(Mor-Cor)Ag(III).** Spectra were obtained from brain samples isolated after intravenous tail vein injection of **(Mor-Cor)Ag(III)**, followed by extraction in CH_3_CN. Each spectrum shows the selected mass region corresponding to the compound’s expected mass, validating its presence in multiple animals. Replicates shown in A and B.


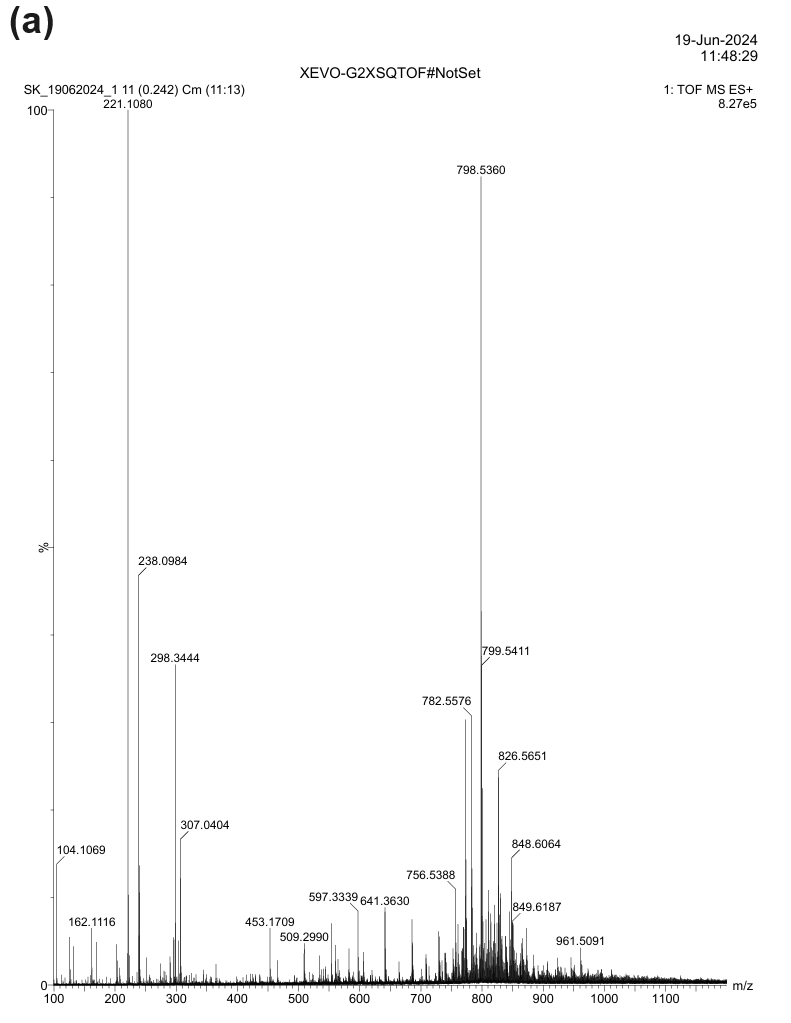


**Figure S25(a)** The ESI-MS spectrum of the sample in CH_3_CN displays the measured spectrum of the full region, observed after intravenous administration of **(Mor-Cor)Ag(III)** into the mouse *via* tail vein injection and subsequent brain isolation.


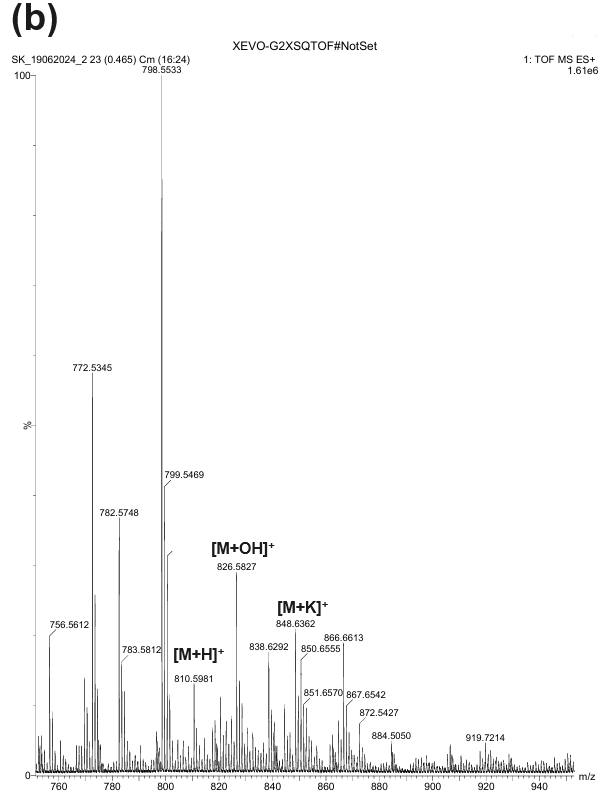


**Figure S25(b)** The ESI-MS spectrum of the sample in CH_3_CN displays the measured spectrum of the selected region, observed after intravenous administration of **(Mor-Cor)Ag(III)** into the mouse *via* tail vein injection and subsequent brain isolation.


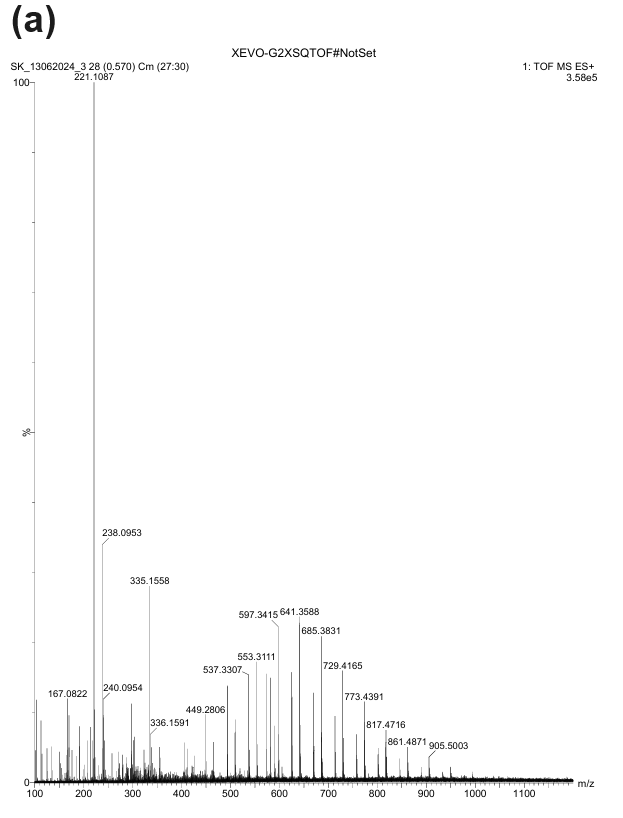


**Figure S26(a)** The ESI-MS spectrum of the sample in CH_3_CN displays the measured spectrum for the entire region, observed without administering **(Mor-Cor)Ag(III)** to the mouse.


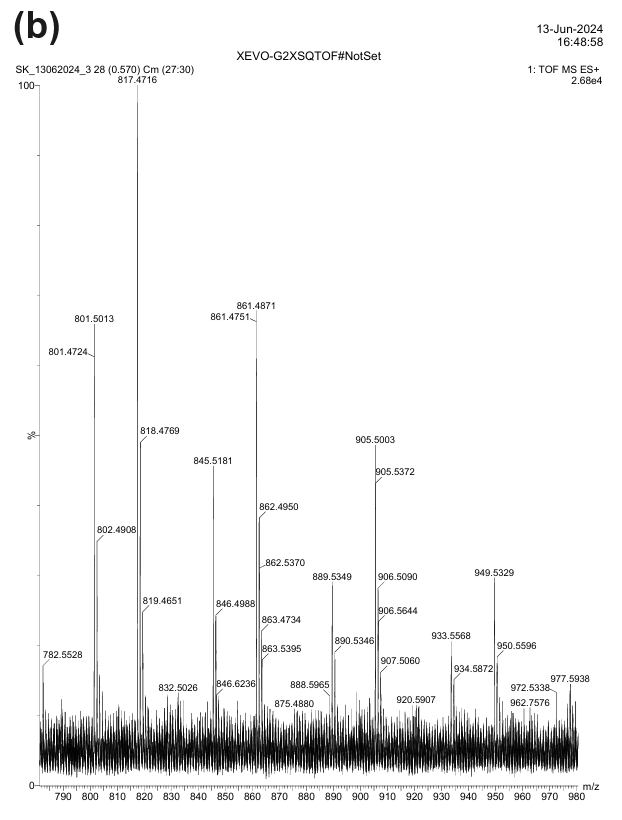


**Figure S26(b)** The ESI-MS spectrum of the sample in CH_3_CN displays the measured spectrum for the selected region, observed without administering **(Mor-Cor)Ag(III)** to the mouse.


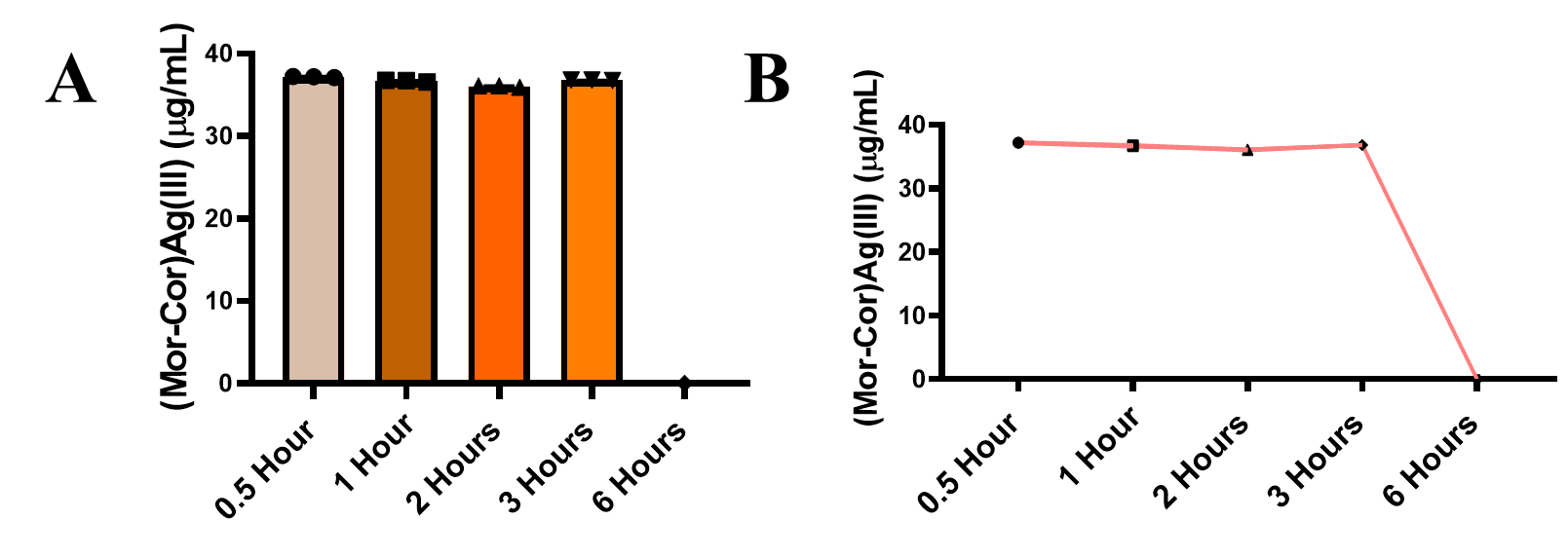


**Figure S27** Plasma pharmacokinetics of **(Mor-Cor)Ag(III)** following tail vein injection. Quantitative UHPLC analysis of plasma samples collected at various time points (0.5 to 6 hours) post-injection shows that **(Mor-Cor)Ag(III)** remains stable in circulation up to 3 hours, with concentrations ranging from ~36–37 µg/mL. A sharp decline is observed at 6 hours, indicating rapid clearance of the compound from systemic circulation.

**Table S6** UHPLC quantification of **(Mor-Cor)Ag(III)** in plasma using a standard calibration curve. Consistent concentrations were detected up to 3 hours; no signal was observed at 6 hours, indicating clearance.


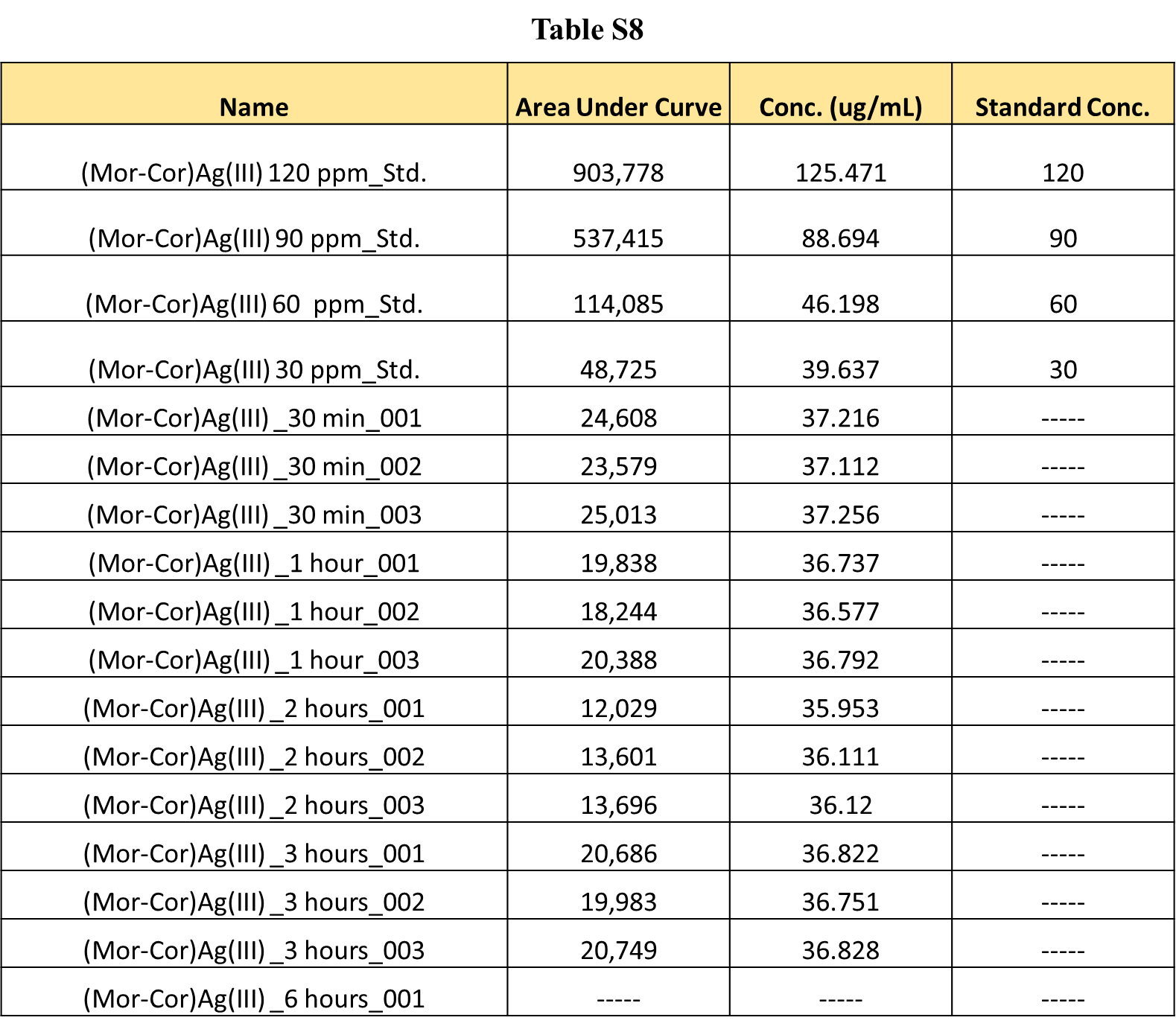


**Appendix 1 Optimized Cartesian Co-ordinates of H_3_(Mor-Cor).**

Cartesian coordinates of the optimized structure (in Å). **H_3_(Mor-Cor)** was optimized at the B3LYP level of theory and 6-311G (d, p) basis set.

**H_3_(Mor-Cor)** (E = -2270.0551hartrees)

N 4.41783 -0.47474 0.41514

O -5.95362 -1.76075 0.53648

N 3.64655 2.01165 0.33232

N 1.06168 1.63599 0.09159

N 1.83374 -1.30511 -0.02027

C 3.83520 -2.80688 0.05715

C 4.78178 -1.74524 0.05651

C -1.91731 -0.72091 0.20983

N -8.94304 -1.70320 -0.30599

C -0.17934 1.04518 0.04468

C 5.39063 0.44738 0.14505

C 2.44829 -2.55753 0.14410

O -11.09745 0.12623 0.10957

C -1.11244 2.12417 -0.08404

H -2.17913 2.00197 -0.17322

C 4.97294 1.80691 0.18803

C -0.47365 -0.33869 0.12933

C 4.32193 -4.19923 -0.03796

C -2.71334 -0.33046 1.29961

H -2.27124 0.25450 2.09797

C 0.45573 -1.39284 0.18146

C 3.41887 3.34848 0.18871

C 1.39647 -3.44721 0.50597

H 1.54069 -4.47907 0.78352

C 5.42494 -4.62790 0.72256

H 5.89924 -3.93374 1.40506

C 6.49492 -0.28775 -0.34382

H 7.42911 0.13364 -0.68098

C 2.10555 3.86229 0.07409

C 0.97374 3.01311 0.02545

C -0.41718 3.31172 -0.08185

H -0.83810 4.30178 -0.14748

C 1.90678 5.32934 -0.04394

C 6.12423 -1.62991 -0.40229

H 6.71714 -2.44069 -0.79467

C 3.71371 -5.12998 -0.89939

H 2.88292 -4.81537 -1.51851

C -3.86147 -1.85771 -0.72625

H -4.28853 -2.44127 -1.53019

C 0.20911 -2.75439 0.52952

H -0.75435 -3.14049 0.81943

C 5.89762 -5.92827 0.63421

H 6.74097 -6.24514 1.23530

C -4.63599 -1.45919 0.36653

C -2.51798 -1.48782 -0.79133

H -1.92975 -1.79541 -1.64854

C -4.04826 -0.69200 1.38102

H -4.65702 -0.40153 2.22875

C 4.17731 -6.43327 -0.99301

H 3.70349 -7.13478 -1.66856

C 5.27587 -6.84612 -0.22502

C -10.34183 -2.12576 -0.42023

H -10.72281 -2.54119 0.53143

H -10.41860 -2.90688 -1.18328

N 1.23610 10.67258 -0.44811

C 4.68823 4.02605 -0.00997

H 4.82910 5.08442 -0.16729

C 1.22162 5.88935 -1.13403

H 0.84364 5.24019 -1.91423

C 1.04752 7.26161 -1.24304

H 0.52594 7.68129 -2.09438

C 5.65729 3.05702 -0.01635

H 6.71920 3.19359 -0.15826

C -6.60464 -2.59158 -0.43082

H -6.08306 -3.55532 -0.48423

H -6.58000 -2.11541 -1.41709

C -8.83627 -0.59945 0.65694

H -7.80224 -0.25764 0.70167

H -9.12576 -0.93156 1.67160

C 1.55913 8.11466 -0.25485

C -8.05127 -2.82146 -0.01662

H -8.40509 -3.68034 -0.59543

H -8.07550 -3.11950 1.04686

C 2.41840 6.19565 0.93543

H 2.93939 5.77989 1.78912

N 6.14682 -9.27393 -0.39546

C 1.38079 9.52951 -0.36169

C 5.75799 -8.18842 -0.31917

C -11.21037 -0.93852 -0.82405

H -10.91285 -0.59453 -1.82624

H -12.26416 -1.22221 -0.84693

C 2.24861 7.56934 0.83727

H 2.64034 8.22548 1.60470

C -9.74419 0.54808 0.23013

H -9.73414 1.34353 0.97732

H -9.39221 0.95769 -0.72867

H 1.99386 1.25315 0.27006

H 2.23913 -0.59945 -0.61806

H 3.56337 -0.21429 0.87721

**___________________________________________________________________**

**Appendix 2** **Optimized Cartesian Co-ordinates of (Mor-Cor)Ag(III).**

Cartesian coordinates of the optimized structure (in Å). **(Mor-Cor)Ag(III)** was optimized at the B3LYP level of theory and 6-311G (d, p) basis set. The LANL2DZ pseudopotential was used for the Ag atom.

**(Mor-Cor)Ag(III)** (E = -2413.9801 hartrees)

Ag -2.42121 0.37767 -0.07807

N -4.19828 -0.48478 -0.16711

O 6.30942 -1.71137 -0.58736

N -3.57843 1.96790 0.05026

N -0.78412 1.50025 -0.20208

N -1.47948 -1.36745 0.04811

C -3.52041 -2.81881 -0.00051

C -4.50981 -1.79817 0.03140

C 2.24860 -0.76695 -0.27018

N 9.28299 -1.64642 0.30662

C 0.48072 0.97247 -0.12295

C -5.26840 0.34805 0.04793

C -2.11947 -2.58455 -0.09306

O 11.40690 0.24434 0.03290

C 1.37173 2.09193 0.04568

H 2.44323 2.02042 0.14164

C -4.92577 1.74098 0.03390

C 0.80324 -0.40959 -0.18900

C -3.98839 -4.22861 0.05747

C 3.05531 -0.29901 -1.32187

H 2.61451 0.32083 -2.09404

C -0.12941 -1.48168 -0.18718

C -3.25630 3.28819 0.01050

C -1.08115 -3.54007 -0.38544

H -1.24153 -4.59164 -0.56391

C -4.96278 -4.69933 -0.83756

H -5.35549 -4.03041 -1.59331

C -6.38038 -0.50173 0.30834

H -7.39376 -0.17973 0.49525

C -1.90719 3.72694 0.00151

C -0.77253 2.87085 -0.03240

C 0.61735 3.23523 0.09006

H 0.98565 4.24221 0.20528

C -1.67293 5.20029 0.06740

C -5.92213 -1.81242 0.30151

H -6.51026 -2.69304 0.50806

C -3.47941 -5.12054 1.01533

H -2.74198 -4.76986 1.72677

C 4.20016 -1.91855 0.63255

H 4.62647 -2.53694 1.41041

C 0.11707 -2.87683 -0.44361

H 1.08044 -3.30138 -0.67626

C -5.41064 -6.01155 -0.78463

H -6.15370 -6.36508 -1.48872

C 4.98400 -1.44445 -0.42317

C 2.84923 -1.57956 0.69601

H 2.25526 -1.94384 1.52633

C 4.39746 -0.63154 -1.40197

H 5.01319 -0.28295 -2.22227

C -3.92295 -6.43349 1.07970

H -3.52990 -7.10800 1.83030

C -4.89355 -6.89102 0.17710

C 10.68782 -2.04813 0.42156

H 11.09276 -2.40179 -0.54505

H 10.76759 -2.86946 1.14081

N -0.89284 10.53451 0.30156

C -4.52132 3.97829 0.02179

H -4.64969 5.04932 -0.00058

C -1.10896 5.79236 1.20612

H -0.84104 5.17014 2.05152

C -0.90524 7.16416 1.27151

H -0.47428 7.61233 2.15823

C -5.53559 3.03159 0.02912

H -6.59647 3.23116 0.03909

C 6.96113 -2.58459 0.34161

H 6.45749 -3.55917 0.33070

H 6.91166 -2.16654 1.35296

C 9.17006 -0.49221 -0.59442

H 8.13031 -0.16807 -0.63793

H 9.48281 -0.76074 -1.62093

C -1.26777 7.97849 0.18954

C 8.41832 -2.76317 -0.06056

H 8.77985 -3.64672 0.47472

H 8.46527 -2.99971 -1.13861

C -2.03446 6.02471 -1.00703

H -2.46409 5.57987 -1.89657

N -5.72245 -9.33754 0.28640

C -1.06042 9.39255 0.25151

C -5.35238 -8.24410 0.23746

C 11.52569 -0.86932 0.90680

H 11.20488 -0.58848 1.92142

H 12.58443 -1.13377 0.93215

C -1.83611 7.39805 -0.95258

H -2.11266 8.02521 -1.79104

C 10.04765 0.64640 -0.08774

H 10.03406 1.48250 -0.78911

H 9.67158 0.99433 0.88617

**________________________________________________________________**
